# Unexpected diversity in Central European Vespoidea (Hymenoptera, Mutillidae, Myrmosidae, Sapygidae, Scoliidae, Tiphiidae, Thynnidae, Vespidae), with description of two species of *Smicromyrme* Thomson, 1870

**DOI:** 10.3897/zookeys.1062.70763

**Published:** 2021-10-14

**Authors:** Christian Schmid-Egger, Stefan Schmidt

**Affiliations:** 1 Fischerstr. 1, 10317 Berlin, Germany Unafiliated Berlin Germany; 2 SNSB-Zoologische Staatssammlung München, Munich, Germany SNSB-Zoologische Staatssammlung München Munich Germany

**Keywords:** Aculeata, DNA barcoding, Germany, German Barcode of Life (GBOL), new species, taxonomy

## Abstract

The present study presents DNA barcoding results for 134 species of Central European Vespoidea, families Mutillidae, Myrmosidae, Sapygidae, Scoliidae, Tiphiidae, Thynnidae, and Vespidae, including DNA barcodes for 100 of the 114 German species. DNA barcoding resulted in unexpected diversity in several families, each with two or more genetic clusters identified by Barcode Index Numbers (BINs). *Smicromyrmeburgeri* Schmid-Egger, **sp. nov.** and *S.langobardensis* Schmid-Egger, **sp. nov.** are described as new from Germany and Italy, respectively. A neotype is designated for *Smicromyrmerufipes* (Fabricius, 1878). The results of DNA barcoding are discussed in respect to detecting cryptic species and refining species limits.

## Introduction

This study represents the first attempt to provide a comprehensive DNA barcode library for wasps of the superfamily Vespoidea for Central Europe, including the families Mutillidae, Myrmosidae, Sapygidae, Scoliidae, Tiphiidae, Thynnidae, and Vespidae. The library was compiled as part of several DNA barcoding projects at the Zoologische Staatssammlung München (ZSM), including the project “Barcoding Fauna Bavarica” and the “German Barcode of Life” projects I and II. DNA barcoding at the ZSM started in 2009 and aimed at assembling DNA barcodes for all Bavarian and German animals (Hendrich et al. 2010, 2014; Hausmann et al. 2011, 2013). Aculeate wasps were already dealt with in earlier data releases, including the bees (Schmidt et al. 2015), apoid wasps (Schmid-Egger et al. 2018), and paper wasps of the genus *Polistes* (Schmid-Egger et al. 2017). The present study covers the Central European Vespoidea excluding the Pompilidae, with a focus on the German fauna, with 114 species currently listed for the country (Table 1; Schmid-Egger 2011). The Pompilidae will be dealt with in a separate data release. In addition to species from neighbouring countries, species from other countries of the Western Palearctic region were included to aid in clarifying the taxonomic status of taxonomically problematic species.

**Tab﻿le 1.** Total number of Vespoidea species included in the present dataset, number of species recorded in Germany, number of German species included in the present data, and German species for which no barcode sequence could be obtained.

Family	Total species	Species recorded in Germany	German species in this work	German species without DNA barcode
Mutillidae	12	11	9	Physetopodaephippium*, *Ronisiabrutia*
Myrmosidae	3	2	2	
Sapygidae	4	4	4	
Scoliidae	11	2	2	
Thynnidae	4	2	2	
Tiphiidae	4	4	4	
Vespidae	96	89	80	*Allodynerusfloricola*, *Ancistrocerusscoticus*, *Odyneruspoecilus*, *O.simillimus*, *Parodontodynerusephippium*, *Pseudepiponaherrichii*, *Stenodynerusdentisquama*, *S.orenburgensis*, *Symmorphusfuscipes*
Totals	134	114	103	

*According to A.S. Lelej (pers. comm.), the name *Physetopodaephippium* is not available and requires replacement.

Pilgrim et al. (2008) proposed a new arrangement of families and subfamilies within the Vespoidea, based on the analysis of molecular data, with several families raised to superfamily level. Here we follow the classification by Aguiar et al. (2013), with all families subsumed under the superfamily Vespoidea s. l., except for the subfamily Myrmosinae, which is treated as a family instead as a subfamily of the Mutillidae, as proposed by Pilgrim et al. (2008). This arrangement at family and subfamily level also reflects the current classification in the Barcode of Life Database (http://www.boldsystems.org).

## Materials and methods

### Sampling

The main source of material includes specimens from the collections of the Zoologische Staatssammlung München (**ZSM**) and from the private collection of Christian Schmid-Egger (**CSE**). If no suitable specimens of German species were available from German territory, specimens collected from neighbouring countries were used, in particular from northern Italy and from the Czech Republic.

For the identification of Vespidae, Neumeyer (2019) was used, for other families Amiet (2008). Mutillidae and Myrmosidae from Central Europe were covered by Lelej and Schmid-Egger (2005), and Thynnidae from Europe and the Palaearctic region by Boni Bartalucci (2004, 2012). A checklist and a red list of German aculeate wasps with all species reported from Germany was provided by Schmid-Egger (2011). Scoliidae were identified using Osten (2000), Sapygidae using Kurzenko and Gusenleitner (1994), and Agnoli (2005) for Methochinae.

For DNA extraction, a single leg was removed from each specimen and sent to the Canadian Centre for DNA Barcoding (**CCDB**) in Guelph, Canada for DNA extraction and barcode sequencing. A complete list of voucher specimens included in the current release is given in Suppl. material 1.

### DNA sequencing

DNA extraction, PCR amplification, and sequencing were conducted at the Canadian Centre for DNA Barcoding (CCDB) using their standardised high-throughput protocols (Ivanova et al. 2006). The 658 bp target region, starting from the 5’ end of the mitochondrial cytochrome *c* oxidase I (COI) gene, includes the DNA barcode region of the animal kingdom (Hebert et al. 2003). Specimens that were successfully sequenced are listed in Suppl. material 1, with country of origin, collection date, sequence lengths and the number of unresolved bases. DNA barcoding statistics with mean and maximum intraspecific distances, distance to nearest species, BIN, country of origin and number of barcoded species are listed in Suppl. material 2. All specimen data are accessible in BOLD as a single citable dataset (https://doi.org/10.5883/DS-GBVEOCE). The data include collecting locality, geographic coordinates, elevation, collector, one or more digital images, identifier, and voucher depository. Sequence data can be obtained through BOLD and include a detailed LIMS report, primer information, and access to trace files.

### Data analysis

Sequence divergence statistics were calculated using the Kimura two parameter model of sequence evolution (Kimura 1980). Sequences were assigned a Barcode Index Number (BIN) by the BOLD system. BINs represent globally unique identifiers for clusters of sequences that correspond closely to biological species (Ratnasingham and Hebert 2013). For aculeate wasps, BINs largely support the taxonomy based on traditional approaches using morphology (Schmidt et al. 2015; Schmid-Egger et al. 2018). For BIN assignment, a minimum sequence length of 500 bp is required, and sequences between 300 and 500 bp can join an existing BIN but will not create new or split existing BINs.

BINs provide an interim taxonomic system that allows defining species as Molecular Taxonomic Units (MOTUs) prior to detailed taxonomic studies including morphology. Sequences were aligned using the BOLD Aligner (amino acid-based hidden Markov models). The analyses are based on sequences with a minimum length of 500 bp and < 1% ambiguous bases. Genetic distances and summary statistics were calculated using analytical tools in BOLD and are given as mean and maximum pairwise distances for intraspecific variation, and as minimum pairwise distances for interspecific variations.

## Results

For the present study, DNA barcode sequences of 868 specimens of Vespoidea were analysed (Suppl. material 1). Of those sequences, 823 had a length of at least 500 bp and less than 1% ambiguous bases and were used for further analyses. Forty-three sequences had a length between 400 and 499 bp, and two sequences between 300 and 399 bp. The sequences of the full dataset represent 134 species, including barcodes of 103 species of German Vespoidea of the families Mutillidae, Myrmosidae, Sapygidae, Scoliidae, Tiphiidae, Thynnidae and Vespidae, representing 90% of the German fauna of these seven families (Table 1). Sequences and distances are presented as a neighbor-joining tree in Suppl. material 3, with BIN assignments indicated by different branch colours.

### Taxonomic treatments

#### Family Mutillidae

##### *Dasylabrismaura* (Linnaeus, 1758)

*Dasylabrismaura* occurs with two subspecies in Central Europe, *D.m.maura* and *D.m.clausa* (Lepeletier, 1845). *Dasylabrism.maura* is restricted in its distribution to Germany and the eastern part of Central and Southern Europe (westwards to Italy). The south-western subspecies, *D.mauraclausa*, occurs from south-western Switzerland to Portugal. Only three of 13 specimens analysed by DNA barcoding yielded sequences, with only one sequence longer than 500 bp, suggesting a primer mismatch issue. The three specimens that yielded usable sequences, two males from Brandenburg in eastern German and a female from the southern Alps in Italy, belong, based on their morphology, to *D.m.maura*.

##### *Physetopodahalensis* (Fabricius, 1787)

In Germany, males of *Physetopodahalensis* occur with two colour variants, including a completely black form and a form with the mesosoma partly red. The colour forms exhibit minor morphological differences but were treated as conspecific (Petersen 1988). In Germany, the red form is restricted in its distribution to eastern Germany. The black form occurs mainly in western parts of Germany, in the state of Baden-Württemberg, but overlaps with the red form in Central and Eastern Europe. Here both forms are regarded to represent valid species, but the taxonomy of this species needs further investigation.

Only four of 17 voucher specimens yielded DNA sequences, presumably, as in the previous species, caused by primer mismatch. The dataset includes sequences of four all-black males. No specimens with red mesosoma were available for analysis. The four specimens with sequence data include three specimens from south-western Germany and one from the Czech Republic. The latter specimen has been assigned a different BIN by the BOLD system. The BIN divergence and the maximum intraspecific diversity of 2.9% indicate that more than one species is subsumed under the name *P.halensis* in the black form, requiring further investigation into the taxonomy of this species, in particular in respect to the taxonomic status of the colour variants.

##### Genus *Smicromyrme* Thomson, 1870

The sequences of *Smicromyrmerufipes* (Fabricius, 1878) from Germany and the southern Alps were assigned to three different BINs. Closer examination indicated the presence of different species that are also characterised by morphological differences. Males of *Smicromyrmerufipes* occur with two colour variants (Petersen 1988), both of which apparently are present in *S.rufipes* s. str., whereas all examined males of the two new species have a partly red mesosoma.

According to Petersen (1988), the type material of *S.rufipes* is lost. He did not designate a neotype, because “for this common species is considered unnecessary”. Considering the results of our barcode analysis, and to fix the type status of the species, we designate a neotype for *S.rufipes* and selected a specimen with a full barcode sequence, as detailed below.

###### Smicromyrme (Smicromyrme) rufipes

Taxon classificationAnimaliaHymenopteraMutillidae

7FE403BC-1AD3-5B51-A254-C9424C24B6B2

Figures 1–7 , 23

Mutilla rufipes Fabricius, 1877: 313 “Habitat Halae Saxonum Dom. Hybner”.

####### Type material.

lost (Petersen 1988).

####### Neotype.

(here designated) Germany • female; Brandenburg, Bad Freienwalde, Gabower Hänge; 52.826°N, 14.080°E; 15 Aug. 2001; Schmid-Egger leg.; coll. ZSM, BC ZSM HYM 10552.

####### Additional material examined.

Apart from the material shown in the list of specimens analysed by DNA barcoding (Suppl. material 1), an additional 78 females from several locations across Germany were examined morphologically, including the German states of Brandenburg, Berlin, Hamburg, Baden-Württemberg, Rhineland-Palatinate, Sachsen-Anhalt, Mecklenburg-Vorpommern.

####### Remarks.

To allow accurate identification of the taxon, a female specimen with full barcode sequence was selected as a neotype. The species was originally described from Halle in Sachsen-Anhalt, about 200 km south-west of the locality from where the neotype was collected. The species agrees with the descriptions of Petersen (1988) and Lelej and Schmid-Egger (2005). For diagnosis and identification see the key to males and females below but note that males cannot be distinguished by morphology from *S.burgeri* sp. nov.

####### Male colour variation.

The males of *S.rufipes* occur in two colour variants without transitional forms (Petersen 1988). We examined 88 males from eastern Germany and Hamburg, which we expected to belong to *S.rufipes*, because no records of *S.burgeri* sp. nov. females are known from these areas. Of those, 52 (59%) are all black and 36 (41%) have at least collare, mesoscutum, and scutellum red. The collare is medially black, and the metanotum and upper mesopleuron are partly red in a few specimens. An additional 46 males of the red form from south-western Germany were also examined, with three specimens each belonging to *S.rufipes* and *S.burgeri* sp. nov., based on their barcode sequences showing that specimens from south Germany cannot be identified to species level. Five specimens from this area without DNA sequences were all black. Considering the distribution of collected females, most males are suspected to belong to *S.rufipes*, and the male black form is much rarer in southern Germany compared to northern and eastern Germany.

**Figures 1–7.**
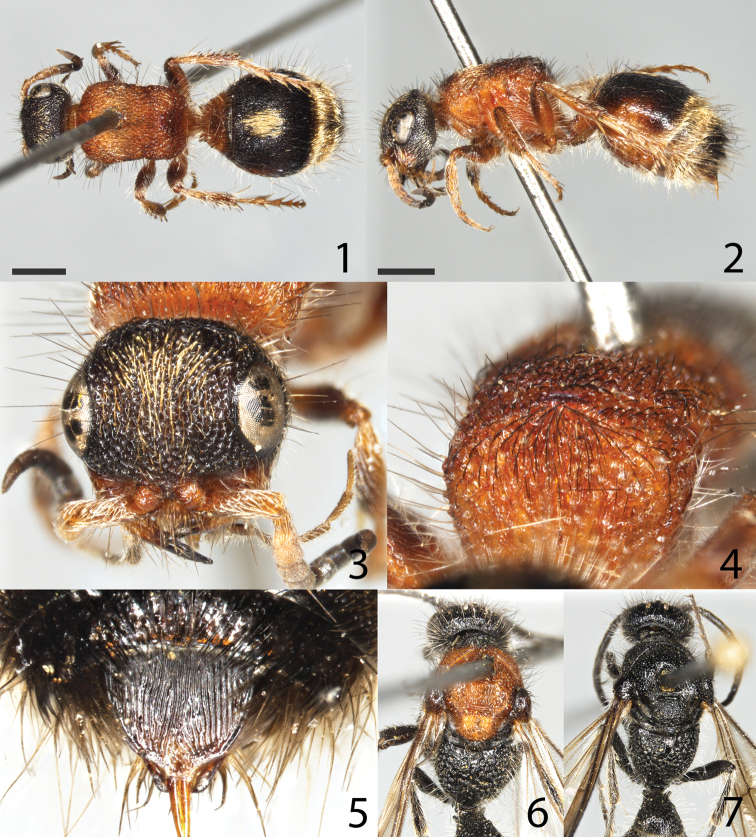
*Smicromyrmerufipes***1–5** female neotype **1** habitus in dorsal view **2** habitus in lateral view **3** head frontal **4** propodeum **5** tergite S6 **6, 7** male **6** male with red mesosoma in dorsal view **7** male with black mesosoma in dorsal view. Scale bars: 1 mm for all images.

####### Distribution.

According to Petersen (1988), *S.rufipes* is widespread in central and northern Europe, eastwards to China and Japan, and also occurring in northern Spain, France, northern and central Italy, Croatia (Krk island) and Serbia (near Belgrade). Specimens mentioned in Petersen (1988) from northern and central Italy, Croatia and Serbia may in fact belong to *S.lombardensis* sp. nov., and specimens from France and Spain to *S.burgeri* sp. nov.

###### Smicromyrme (Smicromyrme) burgeri

Taxon classificationAnimaliaHymenopteraMutillidae

Schmid-Egger sp. nov.

8F1FF506-3E19-5AE7-A55F-0A1D8D317E97

http://zoobank.org/A0DDE87B-2890-46C0-9E8C-3D22188EFBB1

Figures 8–13 , 24

####### Material.

***Holotype*** Germany • female; Bavaria, Nürnberg, E Zirndorf NSG Hainberg; 49.43°N, 10.99°E; 23 Jun. 2008; Schmid-Egger leg.; coll. ZSM, BC ZSM HYM 10590. ***Paratypes*** Germany – **Bavaria** • 3 females; same collecting data as holotype, leg. Schmid-Egger leg.; BC ZSM HYM 10590, BC ZSM HYM 10591, BC ZSM HYM 10592. – **Hesse** • 1 male; Nauheim; 49.94°N, 8.44°E; 16 Jul. 2008; G. Reder leg.; BC ZSM HYM 08149 • 1 male; Bavaria, Nürnberg, Tennenloher Forst; 49.57°N, 11.04°E; 22 Jun. 2008; Schmid-Egger leg.; BC ZSM HYM 06577. – **Rhineland-Palatinate** • 1 female; Birkenheide; 49.49°N, 8.27°E; 2 Jul. 2008; G. Reder leg., BC ZSM HYM 08195 • 1 male; Monsheim; 49.63°N, 8.21°E; 5 Jul. 2008, G. Reder leg.; BC ZSM HYM 08148. France • 1 male; Alpes Martimes, Col de Cayolle, 1.5 km S; 44.244°N, 6.756°E; 1890 m a.s.l.; 14 Jul. 2010; Schmid-Egger leg.; BC ZSM HYM 10617 (all in coll. CSE and ZSM).

####### Additional material examined.

Specimens without barcode sequence, excluded from paratype series: Germany – **Rhineland-Palatinate** • 1 female; Hagenbach; 49.02°N, 8.20°E; 13 Jun. 2009; G. Reder leg., • 1 female; Wachenheim; 49.44°N, 8.17°E; 9 Aug. 1996, Schmid-Egger leg.; • 1 female; Ingelheim; 49.99°N, 8.06°E; Schmid-Egger leg.; – **Baden-Württemberg** • 1 female; Grißheim; 47.87°N, 7.57°E; 27 Jul. 1997; Schmid-Egger leg., • 1 female; Kronau; 49.22°N, 8.62°E; 17 Jul. 1989; Schmid-Egger leg.; • 1 female; Müllheim Schwärze; 47.81°N, 7.68°E; 26 Jul. 1992; France • 1 female; northern Vosges, Niederbronn-les-Bains; 48.95°N, 7.63°E; 2 Aug. 1991; Schmid-Egger leg. (all in coll CSE).

####### Diagnosis.

*Smicromyrmeburgeri* sp. nov. resembles *S.rufipes* but the female has the frons with short dark setae, whereas *S.rufipes* has the frons with a distinct patch of long, golden setae. The golden setae may be shorter in small specimens of *S.rufipes* but they are always distinctly visible and allow a reliable identification. Females from traps with worn setation cannot be reliably identified. For the separation of *S.burgeri* sp. nov. from *S.langobardensis* sp. nov., see under this species.

####### Description.

***Holotype* female.** Body length 5.5 mm. Colour. Black with the following parts red: clypeus, antennal base, antennomeres 1–4 (antennal apex black), mandible apart from black apex, mesosoma apart from black spot on pronotum medially, first tergite laterally, legs (tibia with some dark above). Morphology. Body with long erect setae, longest setae as long as fore tibia. Setae on dorsal side of body dark, lateral setae, and setae on underside of body white. Frons and mesosoma above with a few black adpressed setae. The following parts with spot or band of silver adpressed setae: tergite II mediobasally with a large, subcircular spot, laterally each with a longitudinal spot, apically with band. Tergite III completely covered with such pilosity. Propodeum with lamella, as large as width of middle flagellomeres. Pygidial area with longitudinal striae, reaching apex, apically somewhat divergent.

**Figures 8–13.**
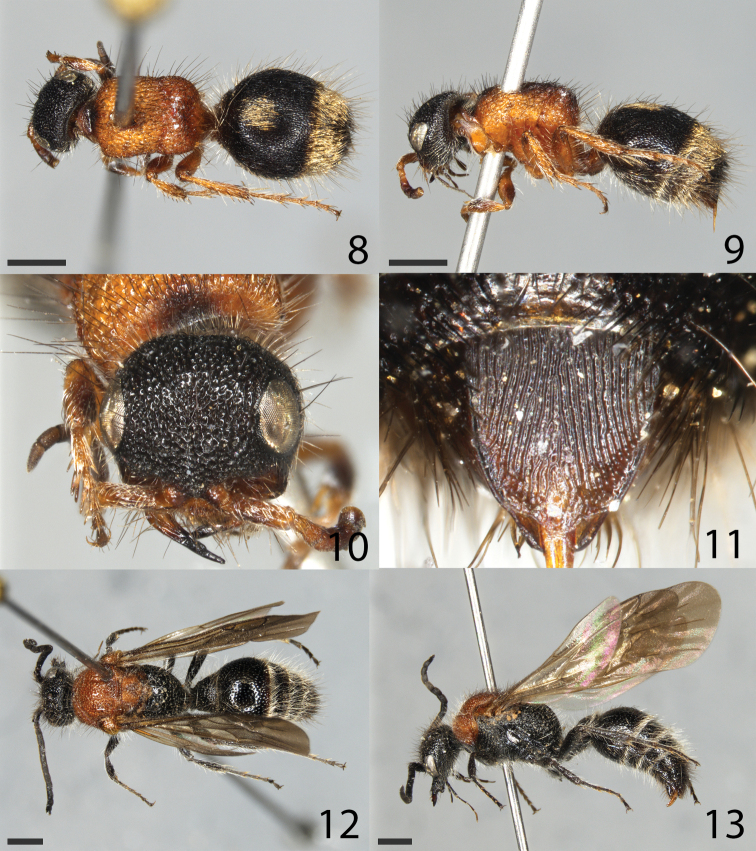
*Smicromyrmeburgeri* sp. nov. **8–11** female holotype **8** habitus in dorsal view **9** habitus in lateral view **10** head in frontal view **11** tergite S6 **12, 13** male paratype **12** habitus in dorsal view **13** habitus in lateral view. Scale bars: 1 mm for all images.

####### Variation.

Female paratypes morphologically similar to the holotype, body length 4.0–5.5 mm.

####### Description of male paratypes.

Body length 7.0–9.0 mm. **Colour.** Black with the following parts red: collare, mesoscutum, scutellum, tegula. The male from France has the collare medially and the tegula black. Males agree in morphology with *S.rufipes* (see key and description in Lelej and Schmid-Egger (2005) and Petersen (1988)). Genitalia see Fig. 24.

####### Distribution.

*Smicromyrmeburgeri* sp. nov. is known from the upper Rhine valley in Germany, an area from near the Swiss border in the south and the Frankfurt area in the north. Other records include sand dunes near Nuremberg in northern Bavaria. In addition, a male from the southern Alps in France was examined. The species has a typical southwest-submediterranean distribution with expansion to south-western Germany. The species is expected to occur elsewhere in southern France and northern Spain. Some records of *S.rufipes* mentioned by Petersen (1988) from southern France and Spain may refer to this species.

####### Etymology.

The species is named after Frank Burger as a specialist for aculeate wasps and bees. He supported the research on Mutillidae by CSE during the initial phase of the project.

###### Smicromyrme (Smicromyrme) langobardensis

Taxon classificationAnimaliaHymenopteraMutillidae

Schmid-Egger sp. nov.

3E9C3962-233A-5228-A1AC-16FF6DDD8A81

http://zoobank.org/025825E4-77FF-4F91-9AEF-D3DE134FFC74

Figures 14–26 , 25

####### Material.

***Holotype*** Italy • male; Lombardia, Valtellina, 10 km E Sondro, Ponte in V.; 46.17°N, 9.96°E; 500 m a.s.l.; 9 Jul. 2006; Schmid-Egger leg.; coll. ZSM, BC ZSM HYM 10620. ***Paratypes*** Italy • 1 male; same collecting data as holotype; BC ZSM HYM 10618 • 1 male; same collecting data as holotype; • 1 female; same locality as holotype; BC ZSM HYM 17467 (all in coll. CSE).

####### Additional material examined.

Female specimens without barcode sequences, excluded as paratypes: 2 females, Italy, Valle d’Aosta, Pondel, 45.67°N, 7.22°E, 7.vii.1995, 25.vii.1999 and Valle d’Aosta, St. Pierre, 45.71°N, 7.23°E, 1.viii.1997, Schmid-Egger leg. (coll. CSE). The females differ from the paratype female in some characters and lack DNA barcode sequences and are therefore not considered in the description (frons and propodeum posteriorly more like *S.rufipes*).

####### Diagnosis.

*Smicromyrmelangobardensis* sp. nov. agrees in most characters with *S.rufipes* and *S.burgeri* sp. nov. All examined males belong to the red form, as described under *S.rufipes* and *S.burgeri* sp. nov., with the exception that the metanotum is always red, whereas it is usually black in the other species. The male is characterised by shape and length of setae of the volsella (lateral view, Fig. 25): basal setae are not longer than medial setae and apically only weakly curved. In *S.rufipes* and *S.burgeri* sp. nov., basal and some of the medial setae are long and bent backwards over the remaining setae (Figs 23, 24). Female frons with some golden adpressed setae, but golden pilosity much sparser than in *S.rufipes*. Lower half of backside of propodeum shiny without punctation or microsculpture in holotype, but always with some punctation or striation in *S.rufipes* and *S.burgeri*.

**Figures 14–18.**
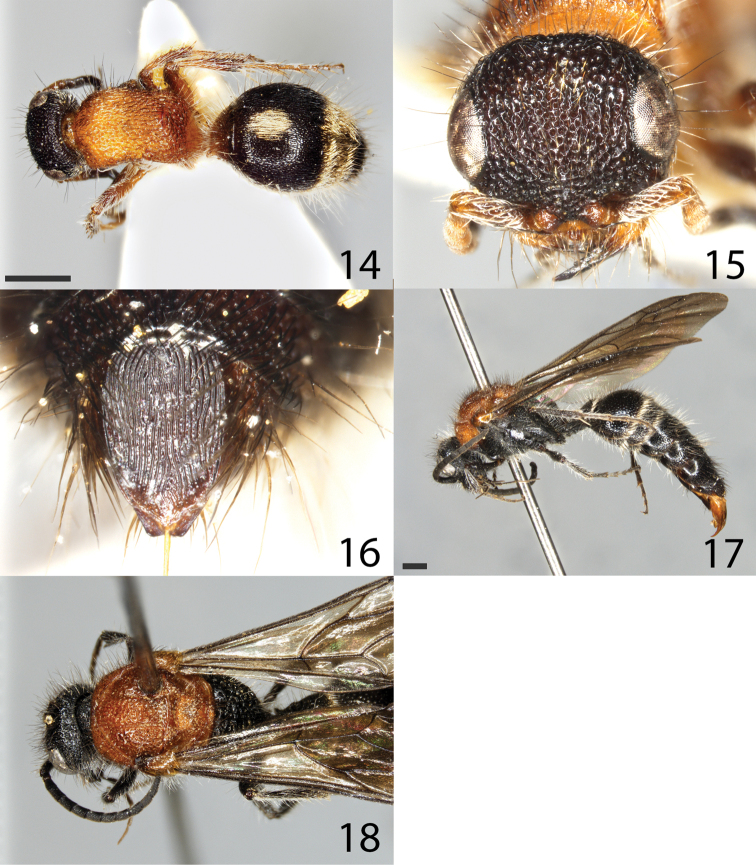
*Smicromyrmelangobardensis* sp. nov. **14–16** female paratype **14** habitus in dorsal view **15** head in frontal view **16** tergite S6 **17, 18** male holotype **17** habitus in lateral view **18** mesosoma in dorsal view. Scale bar: 1 mm for all images.

####### Description.

***Holotype* male.** Body length 12.0 mm. **Colour.** Black with the following parts red: pronotum (pronotal base black), mesoscutum, scutellum, metanotum, tegula. Body with long white erect setae, setae of frons (apart vertex), scutellum and tergites VI and VII black. Morphology. Clypeus in basal half with keel, apically flat with two tubercles in lower third and two tubercles near apical margin. Mandible distinctly curved, with inner tooth near apex. Otherwise like *S.rufipes*. Genitalia see Fig. 25.

####### Variation in male *paratypes*.

Body length 8.0–12.0 mm. Colour of paratypes agrees with holotype except one male with darker red on mesosoma, and only pronotum laterally bright red as in remaining males.

**Figures 19–22.**
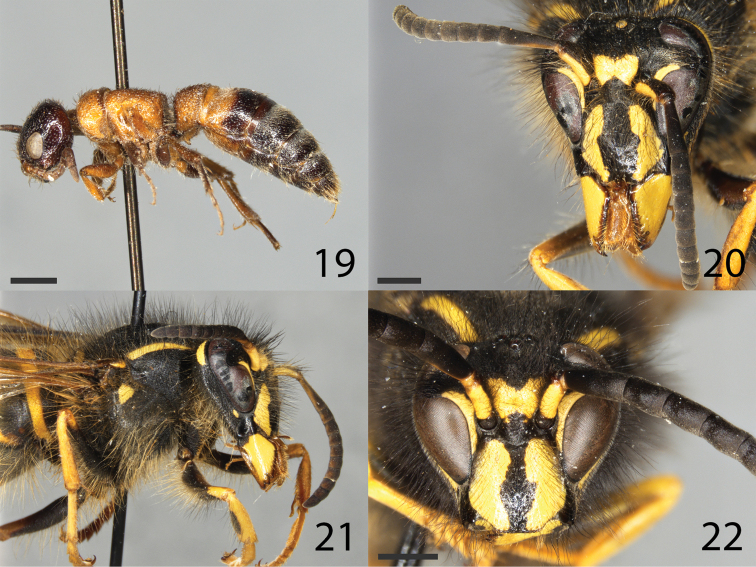
**19** habitus of *Paramyrmosabrunnipes* in lateral view **20–22***Dolichovespulapacifica*. **20, 21** female queen **20** head in frontal view **21** head and mesosoma in lateral view **22** male: head in frontal view. Scale bar: 1 mm for all images.

**Figures 23–26.**
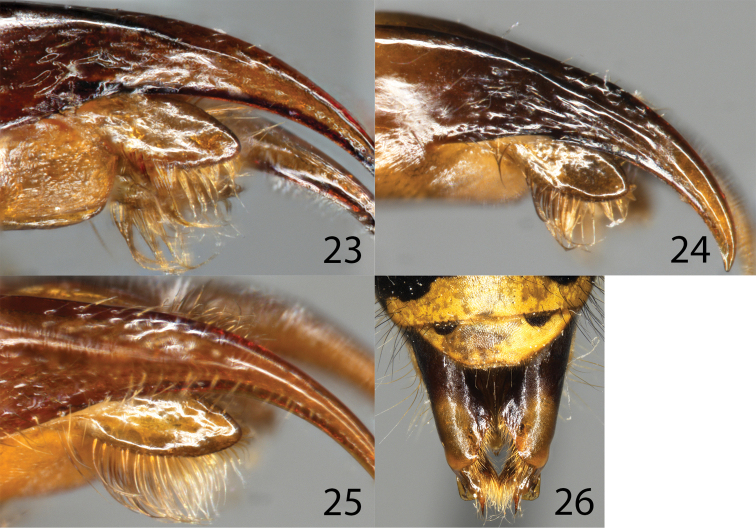
Genitalia, males **23***Smicromyrmerufipes***24***Smicromyrmeburgeri* sp. nov. **25***Smicromyrmelangobardensis* sp. nov. **26***Dolichovespulapacifica*.

####### Description of female paratype.

Body length 5.0–5.5 mm. Agrees in all aspects with females of *S.burgeri* sp. nov. and *S.rufipes* apart from colour and extend of frontal setae. Frons bears 8–10 golden adpressed and isolated setae in *S.langobardensis* sp. nov., not forming a dense patch as in *S.rufipes*.

####### Distribution.

The species is only known from two localities in the Italian Alps, near the border to Switzerland. Records of *S.rufipes* from northern and central Italy and the Balkans (Petersen 1988) may also belong to this species.

####### Etymology.

The species is named after the type locality, the province Langobardia in northern Italy.

### Identification key to species of the *Smicromyrmerufipes* species group

All species treated here key out as *S.rufipes* in the keys of Lelej and Schmid-Egger (2005) and Amiet (2008).

**Males**

**Table:** 

1	Basal setae of volsella in lateral view weakly curved backwards, not much longer than medial setae (Fig. 25), metanotum red (Figs 17, 18), northern Italy (only known by red form)	***Smicromyrmelangobardensis* sp. nov.**
–	Basal or some of the medial setae long and bent backwards over the remaining setae Figs 23, 24. Metanotum usually black. Central Europe north of the Alpes, France (with red and black forms, the latter probably includes only *S.rufipes*)	***Smicromyrmeburgeri* sp. nov. and *S.rufipes***

**Females**

**Table:** 

1	Frons with a few black appressed setae only, without golden setae, Fig. 10. (Lower half of backside of propodeum with some punctures). SW Germany, SE France	***Smicromyrmeburgeri* sp. nov.**
–	Frons with long golden appressed setae, Figs 3, 15	**2**
2	Central Europe north of the Alps. Frons with large spot of golden setae, Fig. 3 (can be worn or reduced in small specimens). Lower half of backside of propodeum with rugae. Central Europe	***Smicromyrmerufipes***
–	Northern Italy. Frons with few (6–8) long golden adpressed setae, Fig. 15. Lower half of backside of propodeum smooth, without punctures or rugae. Northern Italy	***Smicromyrmelangobardensis* sp. nov.**

#### Family Myrmosidae

The family Myrmosidae was formerly treated as a subfamily of the Mutillidae. In Germany two species occur, *Myrmosaatra* and *Paramyrmosabrunnipes* (Lepeletier, 1845) (Fig. 19). The latter has only recently been discovered in Germany, with a specimen collected in 1951 in Hamburg (CS-E, pers. obs.). For the present study, a more recently collected specimen from France was available for barcoding. Additionally, barcodes of two males of *Krombeinellathoracica* (Fabricius, 1793) from Croatia and from Sicily were obtained, each of which was assigned a different BIN, indicating that they may belong to different species, but the few specimens at hand do not allow a thorough taxonomic treatment of this taxon.

##### *Myrmosaatra* Panzer, 1801

*Myrmosaatra* is a widespread and common species in Germany. To our surprise, the barcoded specimens exhibited BIN divergence (see Supp. 2 and 3). Most specimens belong to one of two BINs, with specimens collected in Germany except the upper Rhine valley, and one additional specimen from Slovakia. Specimens of the second BIN were collected in the upper Rhine valley. The area is known for its fauna including south-western Mediterranean species that extend into the south-western parts of Germany. Other examples include *Smicromyrmeburgeri* sp. nov. and the widespread species *Smicromyrmerufipes*. This distribution pattern and a maximum intraspecific distance of 8.8% in *M.atra* suggest the presence of more than one species. However, examination of male morphology did not yield any characters that would allow separation of the two BINs, and no females with barcodes were available from south-western Germany for morphological analysis.

Males from southwestern Germany are, on average, slightly smaller than specimens from other parts of Germany, and the propodeum has a shiny and smooth area laterally, whereas it is sculptured in specimens from other parts of Germany. However, this character varies in specimens that belong to populations of the widespread BIN, with small males also lacking the shiny area on the propodeum. We regard it as probable that *M.atra* consists of two species, but because of the apparent absence of morphological characters and with relatively few barcoded specimens at hand, we refrain from formally describing a new species.

#### Family Sapygidae

DNA barcoding results of all four German species agree with species as traditionally defined using morphological characters.

#### Family Scoliidae

The family Scoliidae is most diverse in the Mediterranean area and occurs with only two species in Germany. Preliminary barcoding results indicated a greater than expected genetic diversity which led us to analyse additional species from Southern Europe.

##### *Campsomeriellathoracica* (Fabricius, 1787)

*Campsomeriellathoracica* is widely distributed in North Africa, southern Europe, and south-western Asia. The species occurs in two geographical male colour forms that are, although questionably (Osten 2000), regarded as subspecies, with a western grey form *C.thoracicasenilis* (Fabricius, 1793), occurring from Tunisia to Morocco, and an eastern red form *C.t.thoracica* in other parts of the distribution area. Females do not exhibit any differences in colour or morphology. It came therefore to a surprise that the species exhibited BIN divergence, with a maximum intraspecific distance of 5.3% and separation into three different BINs.

Specimens from Morocco and Tunisia, agreeing in colour with the *C.thoracicasenilis*, were assigned a different BIN than specimens from Egypt that represented *C.t.thoracica*, with a genetic distance of 2.2% between the two BINs. A third BIN was assigned to specimens from the United Arab Emirates. The red-coloured metasoma of males from the UAE suggested that they belong to *C.t.thoracica* but showed a genetic distance of 4.3% to its nearest neighbour in BOLD, an unidentified species of *Campsomeriella*, and probably represents a different species.

##### *Megascoliamaculata* (Drury, 1773)

*Megascoliamaculata* is widespread in the Mediterranean region and southern Central Europe. It is the largest scoliid wasp and, for that matter, the largest Hymenopteran species in the Western Palaearctic region. Traditionally three subspecies have been recognized (Osten 2000). *Megascoliam.flavifrons* (Fabricius, 1775) occurs in the western Mediterranean area (including Italy) and is characterised by black abdominal setae. In the eastern part of the Mediterranean area and Central and Eastern Europe (including Hungary) *M.m.maculata* is present with red setae at the apex of the metasoma. The nominal subspecies also occurs in a small area in southwest France (Landes region) within the distribution area of the *M.maculataflavifrons*. A third subspecies *M.maculatabarbara* (Micha, 1927) is found in Cyprus.

DNA barcoding of specimens representing all three subspecies did not show any genetic sub-clustering, and in contrast, they mix completely. DNA barcoding therefore does not provide support for their subspecies status and considering the partly overlapping distribution, the current subspecies represents geographically based colour variation and should be regarded as such.

##### *Micromeriellahyalina* (Klug, 1832)

*Micromeriellahyalina* occurs from the Canary Islands and Morocco to Central Asia. Osten (2000) lists the nominal subspecies from North Africa and Israel, and an eastern subspecies *M.hyalinaangulata* (Morawitz, 1888) from Israel to Central Asia. All analysed specimens belong to *M.h.hyalinata*, with pale setae on the metasoma. Specimens from the United Arab Emirates also belong to the nominal subspecies but exhibited BIN divergence, with three different BINs, that possibly represent distinct species.

#### Family Tiphiidae

The family Tiphiidae occurs with four species in Germany, with one of them, *T.femorata* (F.), showing a high level of unexpected BIN divergence.

##### *Tiphiafemorata* (Fabricius, 1775)

*Tiphiafemorata* is a widespread and, like other species of the subfamily Tiphiinae, a parasitoid of soil-dwelling beetle larvae (Coleoptera: Scarabaeoidea, Brothers and Finnamore 1993). The species is morphologically variable, indicated by the presence of several synonyms, but has never been thoroughly revised. Considering the long taxonomic history of this species in Europe, the BIN divergence that was discovered, with four BINs in Germany and a fifth BIN in south-western France, came as a surprise and indicates the presence of several species that were previously overlooked by taxonomists. However, morphological examination of the putative species did not reveal any consistent differences between representatives of each BIN. Probably *T.femorata* consists of several species, but because of the apparent absence of morphological characters and with relatively few barcoded specimens we refrain from formally describing new species.

#### Family Vespidae

In Germany, the Vespidae are represented by four subfamilies, including the Eumeninae, Masarinae, Vespinae, and Polistinae. A recent study of the latter subfamily revealed substantial BIN divergence in some species (Schmid-Egger et al. 2017). In the present release we included representatives of all German *Polistes* species to provide a complete dataset of German Vespidae. In addition, the Asian Hornet *Vespavelutina* Lepeletier, 1836 was included, a species that has recently been introduced into Germany (Witt 2015), and the Oriental Hornet *Vespaorientalis* Linnaeus, 1771 from south-eastern Europe. The dataset also includes DNA barcodes of the rare northern species *Dolichovespulapacifica* (Birula, 1930) (Figs 20–22, 26). The species occurs in Northern Europe, southwards to central Sweden, and in northern Russia east to the Far East. With these additions included, the present dataset comprises all Western Palaearctic species of social wasps (Vespinae and Polistinae).

The subfamily Masarinae is represented by two Central European species, *Celonitesabbreviatus* (Villers, 1798), and *C.rugiceps* Bischoff, 1928, the latter of which is regarded to be extremely rare or even extinct in Germany (Mauss and Prosi 2013). BIN divergence was detected between Central European *C.abbreviatus* and specimens from northwest Italy, and thus shows a similar pattern as *Smicromryrmerufipes* from Central Europe and *S.langobardensis* sp. nov. from the southwestern Italian Alps. Two additional species from the southern French Alps were also included, *Ceramiustuberculifer* de Saussure, 1854, and *Celonitesmayeti* Richards, 1962.

The results of DNA barcoding largely support the previously defined taxonomy of the family (Neumeyer 2019), except in species of Eumeninae and in the genus *Dolichovespula*, as detailed below.

##### *Ancistrocerusrenimacula* (Lepeletier, 1841) and *Ancistrocerusauctus* (Fabricius, 1793)

Both species are widespread in southern Europe, but rare in Germany. In the past they were regarded as conspecific or as subspecies, until Neumeyer (2019) demonstrated their status as distinct species. Results from DNA barcoding confirm their species status, with both species exhibiting a distinct barcode gap and a nearest neighbour distance of 9.1%, similar to the average distance in other species of genus *Ancistrocerus*.

##### *Eumenescoarctatus* (Linnaeus, 1758)

*Eumenescoarctatus* is a common species in Europe except from the northern parts. The species is traditionally regarded to consist of two subspecies, the mainly Central European *E.c.coarctatus* and *E.coarctatuslunulatus* Fabricius, 1804 from southern Europe, with differences in colour patterns between the two taxa (see Neumeyer, 2019, who treats *E.coarctatuslunulatus* as a synonym of *E.coarctatus*).

DNA barcoding revealed BIN divergence between specimens from Cyprus showing the colour pattern of *E.coarctatuslunulatus*, specimens from Germany with the colour pattern of the nominal subspecies, and specimens from northern Italy and France with a transitional colour pattern. The barcoding results suggest the presence of several species under *E.coarctatus* and, in addition, suggest that *E.coarctatuslunulatus* in fact represents a distinct species, demonstrating the need for further taxonomic study of this species complex.

##### *Eumenesdubius* Saussure, 1852

*Eumenesdubius* has not been recorded from Central Europe, but it is widespread in southern Europe. The species exhibits an intraspecific distance of 5.7%, with specimens from Cyprus and Spain assigned to two different BINs, suggesting that at least two species are subsumed under *E.dubius*.

##### *Dolichovespulasylvestris* (Scopoli, 1763)

The species is common and widely distributed in Germany and was represented by three different BINs, each with a distinctive distribution pattern. All specimens from Germany were assigned to the same BIN, whereas two specimens from southern France and a specimen from north-eastern Italy belong to separate BINs, a pattern that was also observed in species of *Smicromyrme*. No morphological characters could be detected that would support the notion of more than one species under *D.sylvestris*.

##### *Leptochilusalpestris* (Saussure, 1855)

*Leptochilusalpestris* includes two BINs with specimens from Italy and southern France. No German specimens of this rare species were available for DNA sequencing. The species is only known from a few local populations in xerothermic areas in south-western Germany (Baden-Württemberg).

#### Family Thynnidae

The small and poorly known wasp family Thynnidae occurs with two species in Germany, including the widespread *Methochaarticulata* (Latreille, 1792), a parasitoid of the larvae of *Cicindela* beetles, and *Meriatripunctata* (Rossi, 1790) that is regarded to be extinct in Germany (Schmid-Egger et al. 2012). Some southern European species of the genus *Meria* Illiger, 1807 showed BIN divergence and are in need of a taxonomic revision.

## Discussion

### DNA barcoding of German Vespoidea

The present study presents DNA barcodes for 134 species of Vespoidea, including 103 (90%) of the 114 species that were recorded from Germany. The release represents, after the bees (Schmidt et al. 2015) and the apoid wasps (Schmid-Egger et al. 2018), the third major DNA barcoding release of German aculeate wasps. Each contribution covered a similar percentage of the German species, with the difference that the species numbers of bees and apoid wasps are, with 584 and 273 species, respectively, considerably larger than the Vespoidea. All three barcode releases together allow the reliable identification of 843 German species of bees and wasps, comprising of about 82% of the German fauna of aculeate wasps and bees. Therefore, virtually any bee or wasp that is collected on German territory can by now be identified using DNA barcoding (barcode releases of the remaining families Pompilidae and Chrysididae, with similar percentages of barcoded species, are currently in preparation). Species that are still missing in our dataset are either extremely rare or even extinct in Germany. Obtaining barcoding sequences of these species will depend on the availability of specimens from countries other than Germany or barcoding of, often historic, specimens from museum collections.

None of the barcoded vespoid species exhibited BIN sharing, and barcodes allow their unambiguous identification. BIN divergence was detected in 15 species (11%), suggesting either large intraspecific variation or presence of undetected species that were not recognised by taxonomists before. In species with BIN diversity, new taxa were formally described if genetic diversity was associated with diagnostic morphological differences. Undoubtedly, DNA barcoding expedited the discovery of the new taxa that would have been very unlikely by a traditional, morphology-based approach.

### Geographic distribution patterns

Several species of Vespoidea show a distribution pattern that appears to be a result of the separation of populations during periods of glaciation, when the species retracted from Central Europe to regions not covered by ice, and their re-invasion during warmer periods (Hewitt 1999; Schmidt 2018). If populations came into contact again during post-glacial warming, differences that accumulated during isolation might prevent them from intermixing, albeit morphologically still very similar (de Lattin 1967). The Upper Rhine Valley in south-western Germany is known as an area with populations of xerothermic species that origin from the Mediterranean area and represent a northern outpost (Westrich 2018; Schmid-Egger 2020; Schmitt 2020). DNA barcoding revealed that several species of Vespoidea seem to reflect this distribution pattern, with one species that is widespread and often common in Central Europe north of the Alpes, and another, morphologically similar species that is restricted in its distribution to the Upper Rhine Valley and sometimes other parts of south-western Germany. Examples include several taxonomically problematic species of the genera *Tiphia*, *Myrmosa*, and *Smicromyrme*. In addition, a third species of each species complex often occurs in northern Italy, northwards into the southern Alps, with its western limits demarcated by the south-western Alpine arc along the border between Italy and France, as in the case of *Smicromyrmelangobardensis* sp. nov. This distribution pattern has long been known in Hymenoptera (de Lattin 1967), including bees and apoid wasps (Schmidt et al. 2015; Schmid-Egger et al. 2018), and can, in some cases, aid in refining species limits in taxonomically problematic taxa. It originates most probably from three retreat areas during ice age, including the Iberian Peninsula, Italy, and the Balkans (Schmitt 2020).

### Unexpected BIN divergence

Vespoidea are generally known for their, compared to most other Hymenoptera, large body size and by their low species diversity, and have therefore for a long time attracted the attention of hymenopterists. Of the 114 species recorded from Germany, more than 90% were described during the 18^th^ and 19^th^ centuries. The taxonomy of most of them has not changed since then and most species are accepted as valid to the present day, except species of taxonomically difficult genera like *Eumenes* Latreille, 1802 (Vespidae, Eumeninae). The BIN divergence of species in several families of the Vespoidea came therefore as a surprise and even led to the discovery and description of new species. BIN divergence was even higher in some species of *Polistes* (Vespidae, Polistinae) that were treated in detail in an earlier study (Schmid-Egger et al. 2017). After more than 100 years of mostly taxonomic stasis in the Vespoidea, DNA barcoding provided the incentive for scrutinising the taxonomy of several species in this group. It laid the foundation for subsequent analyses using integrative approaches combining DNA sequences and morphology, and analysis of supplementary characters systems that allow to refine the species boundaries, for example cuticular hydrocarbon profiles (Soon et al. 2021).

In absence of morphological differences between representatives of different BINs within a traditional species, we refrained from establishing new species, an approach that has also been followed during previous DNA barcoding studies, even when high levels of genetic diversity were detected within a traditional species (Schmid-Egger et al. 2017; Hansson and Schmidt 2018). In such cases, a thorough taxonomic study led to the discovery of morphological differences that were not detected by taxonomists before, but probably would have been discovered when the species question would have been scrutinised by specialists. However, in absence of any new taxonomic insights that would have triggered a taxonomic re-evaluation, species would have most probably been overlooked. DNA barcoding provided these cues and, for example, led to the description of a new species of velvet ants from Germany after more than 130 years. Other examples with DNA barcoding triggering taxonomic discovery included discovery of sibling species in the genus *Tachysphex* from Central Europe (Straka 2016) and the Alpine bee *Andrenaamieti* Praz, Müller & Genoud, 2019. The species was discovered during previous DNA barcoding efforts on German bees (Schmidt et al. 2015) but was only recently described as part of a comprehensive species level revision (Praz et al. 2019).

Species with BIN divergence but without any morphological characters to support the species status of BIN representatives remain problematic. These include species like *Myrmosaatra* and *Tiphiafemorata*, with two and five BINs, respectively, and maximum intraspecific distances of 8.9% in *M.atra* and 13.1% in *T.femorata*. If these genetic distances signify species diversity, then they are not correlated with equally pronounced morphological differences. Morphological examination did not reveal any consistent differences that would allow their separation, and we therefore left the species status of both species as is until further evidence will allow to decide this way or the other. This situation may seem unsatisfactorily at first sight, but it demonstrates a major difference between conventional and barcode-aided taxonomy. Traditionally, one was left with a species name, resulting either from the identification of a specimen using a key, or a name provided by a specialist, usually based on the most recent taxonomic revised of the taxon in question. However, a species name alone does not convey any information about intraspecific variation, nor distances to related species, information that would be critical for assessing the identity of a particular specimen. A name in combination with a DNA barcode, on the other hand, allows sequences and genetic distance information that accurately characterise a specimen to be carried over to subsequent taxonomic treatments aiding in resolving the taxonomic status, in particular if this information is publicly accessible. This would avoid losing information like distribution and life history traits, as it can happen when erroneously lumping species (Sharkey et al. 2021). The name, in combination with the DNA sequence and the Barcode Index Number, allows a much more accurate characterisation of species than morphology alone. Furthermore, and unlike morphological data, from sequences distance measures can be calculated between specimens, populations and species and continuously re-assessed as more barcodes are added to the reference database. Once the barcode structure of a species is has been assessed, it can be accurately and reliably identified and referred to in scientific research.

For species with an intraspecific variation larger than the commonly applied genetic distance threshold of 2%, and with multiple BINs, the information should be available “as is” in the globally accessible Barcode Life Database, including sequence data, photographs, collecting data and other supplementary information. Subsequent taxonomic assessment of these taxa based on morphology alone will most likely not aid much in refining species boundaries, and we therefore propose to use the current name in combination with an identifier like the Barcode Index Number (BIN) and a link to the auxiliary information in BOLD until sufficient data is available to clarify complexes of taxonomically problematic species.

## Supplementary Material

XML Treatment for Smicromyrme (Smicromyrme) rufipes

XML Treatment for Smicromyrme (Smicromyrme) burgeri

XML Treatment for Smicromyrme (Smicromyrme) langobardensis

## Supplementary materials

Supplementary material 1 List of voucher specimen Data type pdf file Explanation note List of voucher specimens with family, species, specimen ID, country of origin,collection date, depository, Barcode Index Number (BIN) and sequencing success (COI fragment length in bp, in square brackets number of unresolved bases). https://binary.pensoft.net/file/599060This dataset is made available under the Open Database License (http://opendatacommons.org/licenses/odbl/1.0/). The Open Database License (ODbL) is a license agreement intended to allow users to freely share, modify, and use this Dataset while maintaining this same freedom for others, provided that the original source and author(s) are credited.Christian Schmid-Egger, Stefan Schmidt

Supplementary material 2 DNA barcoding statistics Data type pdf file Explanation note Barcoding statistics of species with mean and maximum intraspecific distance, nearest neighbour species, minimum distance to nearest neighbour species, Barcode Index Number (BIN), country of origin and number of specimen. Distances are based on the Kimura 2-P model. https://binary.pensoft.net/file/599061This dataset is made available under the Open Database License (http://opendatacommons.org/licenses/odbl/1.0/). The Open Database License (ODbL) is a license agreement intended to allow users to freely share, modify, and use this Dataset while maintaining this same freedom for others, provided that the original source and author(s) are credited.Christian Schmid-Egger, Stefan Schmidt

Supplementary material 3 Neighbor-joining tree resulting from the analysis of DNA barcode sequences Data type pdf file Explanation note Neighbour-joining tree resulting from the analysis of DNA barcode (COI) sequences. Terminal taxa information include Sample ID, species, sex, country, province and (if available) Barcode Index Number (BIN). https://binary.pensoft.net/file/599062This dataset is made available under the Open Database License (http://opendatacommons.org/licenses/odbl/1.0/). The Open Database License (ODbL) is a license agreement intended to allow users to freely share, modify, and use this Dataset while maintaining this same freedom for others, provided that the original source and author(s) are credited.Christian Schmid-Egger, Stefan Schmidt
