# Supplementary material for: Unexpected diversity in Central European Vespoidea (Hymenoptera, Mutillidae, Myrmosidae, Sapygidae, Scoliidae, Tiphiidae, Thynnidae, Vespidae), with description of two species of Smicromyrme Thomson, 1870
Source: Zookeys. 2021 Oct 14;1062:49–72. doi: 10.3897/zookeys.1062.70763 (PMC8530993; doi:10.3897/zookeys.1062.70763)
Supplement: Supplementary material 1 — List of voucher specimen [file zookeys-1062-049-s001.pdf]

Family	Species	Specimen ID	Country	Collection Date	Depository	BIN	COI-5P
Mutillidae	Dasylabris maura	BC ZSM HYM 06268	Germany	03-Jul-2008	SNSB, Zoologische Staatssammlung Muenchen		422[0n]
Mutillidae	Dasylabris maura	BC ZSM HYM 06267	Germany	03-Jul-2008	SNSB, Zoologische Staatssammlung Muenchen		422[0n]
Mutillidae	Dasylabris maura	BC ZSM HYM 06266	Italy	17-Jun-2009	SNSB, Zoologische Staatssammlung Muenchen	BOLD:ABA1491	519[3n]
Mutillidae	Mutilla europaea	BC ZSM HYM 06364	France	15-Jul-2010	SNSB, Zoologische Staatssammlung Muenchen		407[0n]
Mutillidae	Mutilla europaea	BC ZSM HYM 10610	France	10-Jul-2010	SNSB, Zoologische Staatssammlung Muenchen		421[0n]
Mutillidae	Mutilla europaea	BC ZSM HYM 06259	France	17-Jul-2009	SNSB, Zoologische Staatssammlung Muenchen		422[4n]
Mutillidae	Mutilla marginata	BC ZSM HYM 06263	Germany	25-Aug-2009	SNSB, Zoologische Staatssammlung Muenchen		422[8n]
Mutillidae	Myrmilla calva	BC ZSM HYM 06625	Croatia	27-Aug-2005	SNSB, Zoologische Staatssammlung Muenchen	BOLD:AAU2040	658[0n]
Mutillidae	Myrmilla calva	BC ZSM HYM 06613	Italy	09-Jul-2006	SNSB, Zoologische Staatssammlung Muenchen	BOLD:AAU2040	658[0n]
Mutillidae	Myrmilla calva	BC ZSM HYM 06601	Italy	27-Jun-1996	SNSB, Zoologische Staatssammlung Muenchen	BOLD:AAU2040	658[1n]
Mutillidae	Myrmilla erythrocephala	BC ZSM HYM 10616	Italy	13-May-2006	SNSB, Zoologische Staatssammlung Muenchen	BOLD:ABA8661	658[0n]
Mutillidae	Myrmilla erythrocephala	BC ZSM HYM 10615	France	12-Jul-2009	SNSB, Zoologische Staatssammlung Muenchen	BOLD:ABA8661	421[0n]
Mutillidae	Physetopoda daghestanica	BC ZSM HYM 10834	Austria	05-Sep-1995	SNSB, Zoologische Staatssammlung Muenchen		421[0n]
Mutillidae	Physetopoda daghestanica	BC ZSM HYM 10569	Hungary	13-Jul-2009	SNSB, Zoologische Staatssammlung Muenchen		421[0n]
Mutillidae	Physetopoda daghestanica	BC ZSM HYM 10568	Hungary	22-Jul-2009	SNSB, Zoologische Staatssammlung Muenchen		421[0n]
Mutillidae	Physetopoda halensis	BC ZSM HYM 10852	Germany	03-Aug-1991	SNSB, Zoologische Staatssammlung Muenchen	BOLD:ABV4512	570[0n]
Mutillidae	Physetopoda halensis	BC ZSM HYM 12853	Czech Republic	30-Jun-2009	SNSB, Zoologische Staatssammlung Muenchen	BOLD:ACC4310	658[0n]
Mutillidae	Physetopoda halensis	BC ZSM HYM 10638	Germany	01-Jul-1994	SNSB, Zoologische Staatssammlung Muenchen	BOLD:ABV4512	658[0n]
Mutillidae	Physetopoda halensis	BC ZSM HYM 10637	Germany	16-Aug-2008	SNSB, Zoologische Staatssammlung Muenchen	BOLD:ABV4512	658[0n]
Mutillidae	Physetopoda scutellaris	BC ZSM HYM 10624	Germany	27-Jun-2011	SNSB, Zoologische Staatssammlung Muenchen		421[0n]
Mutillidae	Physetopoda scutellaris	BC ZSM HYM 10607	Germany	01-Jul-2008	SNSB, Zoologische Staatssammlung Muenchen		421[0n]
Mutillidae	Physetopoda scutellaris	BC ZSM HYM 10605	Germany	01-Jul-2008	SNSB, Zoologische Staatssammlung Muenchen		421[0n]
Mutillidae	Physetopoda scutellaris	BC ZSM HYM 06639	Germany	01-Jul-2008	SNSB, Zoologische Staatssammlung Muenchen		422[0n]
Mutillidae	Physetopoda scutellaris	BC ZSM HYM 06612	Germany	01-Jul-2008	SNSB, Zoologische Staatssammlung Muenchen		422[0n]
Mutillidae	Physetopoda scutellaris	BC ZSM HYM 06600	Germany	01-Jul-2008	SNSB, Zoologische Staatssammlung Muenchen		422[0n]
Mutillidae	Physetopoda scutellaris	BC ZSM HYM 06588	Germany	01-Jul-2008	SNSB, Zoologische Staatssammlung Muenchen		422[0n]
Mutillidae	Smicromyrme burgeri	BC ZSM HYM 10626	Germany	23-Jun-2008	SNSB, Zoologische Staatssammlung Muenchen	BOLD:AAU3642	658[0n]
Mutillidae	Smicromyrme burgeri	BC ZSM HYM 10617	France	14-Jul-2010	SNSB, Zoologische Staatssammlung Muenchen	BOLD:AAU3642	654[0n]
Mutillidae	Smicromyrme burgeri	BC ZSM HYM 10593	Germany	23-Jun-2008	SNSB, Zoologische Staatssammlung Muenchen	BOLD:AAU3642	631[0n]
Mutillidae	Smicromyrme burgeri	BC ZSM HYM 10592	Germany	23-Jun-2008	SNSB, Zoologische Staatssammlung Muenchen	BOLD:AAU3642	658[0n]
Mutillidae	Smicromyrme burgeri	BC ZSM HYM 10591	Germany	23-Jun-2008	SNSB, Zoologische Staatssammlung Muenchen	BOLD:AAU3642	658[0n]
Mutillidae	Smicromyrme burgeri	BC ZSM HYM 10590	Germany	23-Jun-2008	SNSB, Zoologische Staatssammlung Muenchen	BOLD:AAU3642	658[0n]
Mutillidae	Smicromyrme burgeri	BC ZSM HYM 06577	Germany	22-Jun-2008	SNSB, Zoologische Staatssammlung Muenchen	BOLD:AAU3642	658[0n]
Mutillidae	Smicromyrme burgeri	BC ZSM HYM 08196	Germany	13-Jun-2009	SNSB, Zoologische Staatssammlung Muenchen	BOLD:AAU3642	658[0n]
Mutillidae	Smicromyrme burgeri	BC ZSM HYM 08195	Germany	02-Jul-2008	SNSB, Zoologische Staatssammlung Muenchen	BOLD:AAU3642	658[0n]
Mutillidae	Smicromyrme burgeri	BC ZSM HYM 08149	Germany	16-Jun-2008	SNSB, Zoologische Staatssammlung Muenchen	BOLD:AAU3642	658[0n]
Mutillidae	Smicromyrme burgeri	BC ZSM HYM 08148	Germany	05-Jul-2006	SNSB, Zoologische Staatssammlung Muenchen	BOLD:AAU3642	658[0n]
Mutillidae	Smicromyrme lombardensis	BC ZSM HYM 17467	Italy	09-Jul-2006	SNSB, Zoologische Staatssammlung Muenchen	BOLD:ABU8217	614[0n]
Mutillidae	Smicromyrme lombardensis	BC ZSM HYM 10620	Italy	09-Jul-2006	SNSB, Zoologische Staatssammlung Muenchen	BOLD:ABU8217	658[0n]
Mutillidae	Smicromyrme lombardensis	BC ZSM HYM 10618	Italy	09-Jul-2006	SNSB, Zoologische Staatssammlung Muenchen	BOLD:ABU8217	658[0n]
Mutillidae	Smicromyrme rufipes	BC ZSM HYM 10552	Germany	15-Aug-2001	SNSB, Zoologische Staatssammlung Muenchen	BOLD:AAU3641	421[0n]
Mutillidae	Smicromyrme rufipes	BC ZSM HYM 10551	Germany	05-Aug-2000	SNSB, Zoologische Staatssammlung Muenchen	BOLD:AAU3641	421[0n]
Mutillidae	Smicromyrme rufipes	BC ZSM HYM 22670	Germany	02-Jul-2012	SNSB, Zoologische Staatssammlung Muenchen	BOLD:AAU3641	513[0n]
Mutillidae	Smicromyrme rufipes	BC ZSM HYM 22649	Germany	19-Jun-2012	SNSB, Zoologische Staatssammlung Muenchen	BOLD:AAU3641	414[2n]
Mutillidae	Smicromyrme rufipes	BC ZSM HYM 10600	Germany	25-Jun-2001	SNSB, Zoologische Staatssammlung Muenchen	BOLD:AAU3641	421[0n]
Mutillidae	Smicromyrme rufipes	BC ZSM HYM 10599	Germany	30-Jun-2001	SNSB, Zoologische Staatssammlung Muenchen	BOLD:AAU3641	421[0n]
Mutillidae	Smicromyrme rufipes	BC ZSM HYM 10597	Germany	03-Jul-2001	SNSB, Zoologische Staatssammlung Muenchen	BOLD:AAU3641	421[0n]

Family	Species	Specimen ID	Country	Collection Date	Depository	BIN	COI-5P
Mutillidae	Smicromyrme rufipes	BC ZSM HYM 10596	Germany	02-Aug-2008	SNSB, Zoologische Staatssammlung Muenchen	BOLD:AAU3641	421[0n]
Mutillidae	Smicromyrme rufipes	BC ZSM HYM 10595	Germany	02-Aug-2008	SNSB, Zoologische Staatssammlung Muenchen	BOLD:AAU3641	421[0n]
Mutillidae	Smicromyrme rufipes	BC ZSM HYM 10594	Germany	15-Jul-2008	SNSB, Zoologische Staatssammlung Muenchen	BOLD:AAU3641	649[0n]
Mutillidae	Smicromyrme rufipes	BC ZSM HYM 10581	Germany	18-Jun-1997	SNSB, Zoologische Staatssammlung Muenchen	BOLD:AAU3641	421[0n]
Mutillidae	Smicromyrme rufipes	BC ZSM HYM 10579	Germany	22-Jul-2001	SNSB, Zoologische Staatssammlung Muenchen	BOLD:AAU3641	421[0n]
Mutillidae	Smicromyrme rufipes	BC ZSM HYM 10550	Germany	05-Aug-2000	SNSB, Zoologische Staatssammlung Muenchen	BOLD:AAU3641	421[0n]
Mutillidae	Smicromyrme rufipes	BC ZSM HYM 10549	Germany	02-Jul-2009	SNSB, Zoologische Staatssammlung Muenchen	BOLD:AAU3641	421[0n]
Mutillidae	Smicromyrme rufipes	BC ZSM HYM 10548	Germany	02-Jul-2009	SNSB, Zoologische Staatssammlung Muenchen	BOLD:AAU3641	421[0n]
Mutillidae	Smicromyrme rufipes	BC ZSM HYM 10547	Germany	03-Jul-2001	SNSB, Zoologische Staatssammlung Muenchen	BOLD:AAU3641	421[0n]
Mutillidae	Smicromyrme rufipes	BC ZSM HYM 10546	Germany	03-Jul-2001	SNSB, Zoologische Staatssammlung Muenchen	BOLD:AAU3641	421[0n]
Mutillidae	Smicromyrme rufipes	BC ZSM HYM 09855	Germany	14-Jun-2011	SNSB, Zoologische Staatssammlung Muenchen	BOLD:AAU3641	421[0n]
Mutillidae	Smicromyrme rufipes	BC ZSM HYM 06624	Germany	02-Jul-2009	SNSB, Zoologische Staatssammlung Muenchen	BOLD:AAU3641	602[0n]
Mutillidae	Smicromyrme rufipes	BC ZSM HYM 06565	Germany	15-Jul-2008	SNSB, Zoologische Staatssammlung Muenchen	BOLD:AAU3641	616[3n]
Mutillidae	Smicromyrme rufipes	BC ZSM HYM 22662	Germany	02-Jul-2012	SNSB, Zoologische Staatssammlung Muenchen	BOLD:AAU3641	670[0n]
Mutillidae	Stenomutilla argentata	BC ZSM HYM 10611	France	10-Jul-2010	SNSB, Zoologische Staatssammlung Muenchen	BOLD:ABV3984	658[0n]
Mutillidae	Stenomutilla argentata	BC ZSM HYM 10612	France	12-Jul-2009	SNSB, Zoologische Staatssammlung Muenchen	BOLD:ABV3984	658[0n]
Myrmosidae	Krombeinella thoracica	BC ZSM HYM 10621	Italy	25-Jun-1999	SNSB, Zoologische Staatssammlung Muenchen	BOLD:ABV3986	615[2n]
Myrmosidae	Krombeinella thoracica	BC ZSM HYM 10614	Croatia	27-Aug-2005	SNSB, Zoologische Staatssammlung Muenchen	BOLD:ABV3985	622[0n]
Myrmosidae	Myrmosa atra	BC ZSM HYM 16992	Germany	18-Jul-2012	SNSB, Zoologische Staatssammlung Muenchen	BOLD:AAY9719	658[0n]
Myrmosidae	Myrmosa atra	BC ZSM HYM 10840	Germany	12-Aug-1992	SNSB, Zoologische Staatssammlung Muenchen	BOLD:AAY9719	421[0n]
Myrmosidae	Myrmosa atra	BC ZSM HYM 10839	Germany	16-Aug-1992	SNSB, Zoologische Staatssammlung Muenchen	BOLD:AAY9719	611[0n]
Myrmosidae	Myrmosa atra	BC ZSM HYM 12887	Slovakia	14-Jun-2008	SNSB, Zoologische Staatssammlung Muenchen	BOLD:AAU3504	658[0n]
Myrmosidae	Myrmosa atra	BC ZSM HYM 24670	Germany	07-Jul-2015	SNSB, Zoologische Staatssammlung Muenchen	BOLD:AAU3504	658[0n]
Myrmosidae	Myrmosa atra	BC ZSM HYM 12276	Germany	01-Jul-1997	SNSB, Zoologische Staatssammlung Muenchen	BOLD:AAU3504	522[0n]
Myrmosidae	Myrmosa atra	BC ZSM HYM 12275	Germany	21-Jun-2001	SNSB, Zoologische Staatssammlung Muenchen	BOLD:AAU3504	658[0n]
Myrmosidae	Myrmosa atra	BC ZSM HYM 10557	Germany	28-Aug-2009	SNSB, Zoologische Staatssammlung Muenchen	BOLD:AAY9719	421[0n]
Myrmosidae	Myrmosa atra	BC ZSM HYM 19937	Germany	07-Jul-2013	SNSB, Zoologische Staatssammlung Muenchen	BOLD:AAU3504	658[0n]
Myrmosidae	Myrmosa atra	BC ZSM HYM 10625	Germany	27-Jun-2011	SNSB, Zoologische Staatssammlung Muenchen	BOLD:AAU3504	658[0n]
Myrmosidae	Myrmosa atra	BC ZSM HYM 10604	Germany	28-Jul-2005	SNSB, Zoologische Staatssammlung Muenchen	BOLD:AAU3504	658[0n]
Myrmosidae	Myrmosa atra	BC ZSM HYM 10603	Germany	22-Jul-2009	SNSB, Zoologische Staatssammlung Muenchen	BOLD:AAU3504	658[0n]
Myrmosidae	Myrmosa atra	BC ZSM HYM 10602	Germany	01-Aug-2008	SNSB, Zoologische Staatssammlung Muenchen	BOLD:AAU3504	658[0n]
Myrmosidae	Myrmosa atra	BC ZSM HYM 10601	Germany	01-Aug-2008	SNSB, Zoologische Staatssammlung Muenchen	BOLD:AAU3504	658[0n]
Myrmosidae	Myrmosa atra	BC ZSM HYM 10587	Germany	10-Jul-2006	SNSB, Zoologische Staatssammlung Muenchen	BOLD:AAU3504	658[0n]
Myrmosidae	Myrmosa atra	BC ZSM HYM 10585	Germany	02-Aug-2001	SNSB, Zoologische Staatssammlung Muenchen	BOLD:AAU3504	658[0n]
Myrmosidae	Myrmosa atra	BC ZSM HYM 10584	Germany	18-Aug-2001	SNSB, Zoologische Staatssammlung Muenchen	BOLD:AAU3504	658[0n]
Myrmosidae	Myrmosa atra	BC ZSM HYM 10583	Germany	27-Jul-2010	SNSB, Zoologische Staatssammlung Muenchen	BOLD:AAU3504	658[0n]
Myrmosidae	Myrmosa atra	BC ZSM HYM 10564	Germany	02-Jul-2003	SNSB, Zoologische Staatssammlung Muenchen	BOLD:AAY9719	658[0n]
Myrmosidae	Myrmosa atra	BC ZSM HYM 10563	Germany	01-Jul-2003	SNSB, Zoologische Staatssammlung Muenchen	BOLD:AAU3504	658[0n]
Myrmosidae	Myrmosa atra	BC ZSM HYM 10560	Germany	30-Jul-2004	SNSB, Zoologische Staatssammlung Muenchen	BOLD:AAY9719	421[0n]
Myrmosidae	Myrmosa atra	BC ZSM HYM 10558	Germany	09-Jun-2009	SNSB, Zoologische Staatssammlung Muenchen	BOLD:AAY9719	658[0n]
Myrmosidae	Myrmosa atra	BC ZSM HYM 09940	Germany	27-Jun-2011	SNSB, Zoologische Staatssammlung Muenchen	BOLD:AAU3504	658[0n]
Myrmosidae	Myrmosa atra	BC ZSM HYM 06257	Germany	01-Jul-2007	SNSB, Zoologische Staatssammlung Muenchen	BOLD:AAU3504	658[0n]
Myrmosidae	Myrmosa atra	BC ZSM HYM 06256	Germany	01-Jul-2007	SNSB, Zoologische Staatssammlung Muenchen	BOLD:AAU3504	658[0n]
Myrmosidae	Myrmosa atra	BC ZSM HYM 06255	Germany	01-Jul-2007	SNSB, Zoologische Staatssammlung Muenchen	BOLD:AAU3504	658[0n]
Myrmosidae	Myrmosa atra	BC ZSM HYM 06254	Germany	01-Jul-2007	SNSB, Zoologische Staatssammlung Muenchen	BOLD:AAU3504	658[0n]
Myrmosidae	Myrmosa atra	BC ZSM HYM 12279	Germany	29-Jul-1997	SNSB, Zoologische Staatssammlung Muenchen	BOLD:AAU3504	610[0n]
Myrmosidae	Myrmosa atra	BC-ZSM-HYM-29774-A03	Germany	15-Jun-2016	SNSB, Zoologische Staatssammlung Muenchen	BOLD:AAU3504	634[0n]

Family	Species	Specimen ID	Country	Collection Date	Depository	BIN	COI-5P
Myrmosidae	Myrmosa atra	BC-ZSM-HYM-29774-A02	Germany	15-Jun-2016	SNSB, Zoologische Staatssammlung Muenchen	BOLD:AAU3504	635[0n]
Myrmosidae	Myrmosa atra	BC-ZSM-HYM-29774-A01	Germany	15-Jun-2016	SNSB, Zoologische Staatssammlung Muenchen	BOLD:AAU3504	631[0n]
Myrmosidae	Myrmosa atra	BC ZSM HYM 08147	Germany	24-Jun-2009	SNSB, Zoologische Staatssammlung Muenchen	BOLD:AAY9719	658[0n]
Myrmosidae	Myrmosa atra	BC ZSM HYM 08146	Germany	20-May-2003	SNSB, Zoologische Staatssammlung Muenchen	BOLD:AAY9719	421[1n]
Myrmosidae	Myrmosa atra	BC ZSM HYM 08145	Germany	09-Jun-2009	SNSB, Zoologische Staatssammlung Muenchen	BOLD:AAY9719	658[0n]
Myrmosidae	Paramyrmosa brunnipes	BC ZSM HYM 06253	France	23-May-1998	SNSB, Zoologische Staatssammlung Muenchen	BOLD:AAV3086	658[1n]
Sapygidae	Monosapyga clavicornis	BC ZSM HYM 19939	Germany	09-May-2013	SNSB, Zoologische Staatssammlung Muenchen	BOLD:AAU3221	658[0n]
Sapygidae	Monosapyga clavicornis	BC ZSM HYM 21076	Germany	08-May-2013	SNSB, Zoologische Staatssammlung Muenchen	BOLD:AAU3221	658[0n]
Sapygidae	Monosapyga clavicornis	BC ZSM HYM 06649	Austria	02-Jul-2006	SNSB, Zoologische Staatssammlung Muenchen	BOLD:AAU3221	658[0n]
Sapygidae	Monosapyga clavicornis	BC ZSM HYM 06626	Austria	02-Jul-2006	SNSB, Zoologische Staatssammlung Muenchen	BOLD:AAU3221	658[0n]
Sapygidae	Monosapyga clavicornis	BC ZSM HYM 06567	Germany	17-Jul-2004	SNSB, Zoologische Staatssammlung Muenchen	BOLD:AAU3221	611[0n]
Sapygidae	Monosapyga clavicornis	BC ZSM HYM 07382	Germany	09-Jun-2010	SNSB, Zoologische Staatssammlung Muenchen	BOLD:AAU3221	658[0n]
Sapygidae	Monosapyga clavicornis	BC ZSM HYM 07377	Germany	09-Jun-2010	SNSB, Zoologische Staatssammlung Muenchen	BOLD:AAU3221	658[0n]
Sapygidae	Monosapyga clavicornis	BC ZSM HYM 07376	Germany	25-Jun-2010	SNSB, Zoologische Staatssammlung Muenchen	BOLD:AAU3221	658[0n]
Sapygidae	Sapyga quinquepunctata	BC ZSM HYM 17465	Spain	08-Apr-2010	SNSB, Zoologische Staatssammlung Muenchen	BOLD:AAV7700	658[0n]
Sapygidae	Sapyga quinquepunctata	BC ZSM HYM 17464	Spain	08-Apr-2010	SNSB, Zoologische Staatssammlung Muenchen	BOLD:AAV7700	658[0n]
Sapygidae	Sapyga quinquepunctata	BC-ZSM-HYM-23654-D12	Czech Republic	03-Jun-2005	SNSB, Zoologische Staatssammlung Muenchen	BOLD:AAV7700	658[0n]
Sapygidae	Sapyga quinquepunctata	BC ZSM HYM 10187	Germany	17-Jul-2011	SNSB, Zoologische Staatssammlung Muenchen	BOLD:AAV7700	658[0n]
Sapygidae	Sapyga quinquepunctata	BC ZSM HYM 06614	Germany	17-Jun-2002	SNSB, Zoologische Staatssammlung Muenchen	BOLD:AAV7700	658[0n]
Sapygidae	Sapyga quinquepunctata	BC ZSM HYM 06591	Italy	15-May-1999	SNSB, Zoologische Staatssammlung Muenchen	BOLD:AAV7700	658[0n]
Sapygidae	Sapyga similis	BC ZSM HYM 06603	Germany	07-Jun-1996	SNSB, Zoologische Staatssammlung Muenchen	BOLD:AAV7701	658[0n]
Sapygidae	Sapyga similis	BC ZSM HYM 06572	France	09-Jul-2010	SNSB, Zoologische Staatssammlung Muenchen	BOLD:AAV7701	658[0n]
Sapygidae	Sapygina decemguttata	BC ZSM HYM 13453	Germany	07-Jun-2007	SNSB, Zoologische Staatssammlung Muenchen	BOLD:AAO1197	658[0n]
Sapygidae	Sapygina decemguttata	BC ZSM HYM 16994	Germany	18-Jul-2012	SNSB, Zoologische Staatssammlung Muenchen	BOLD:AAO1197	658[0n]
Sapygidae	Sapygina decemguttata	BC ZSM HYM 09224	Germany	29-Jul-1996	SNSB, Zoologische Staatssammlung Muenchen	BOLD:AAO1197	573[0n]
Sapygidae	Sapygina decemguttata	BC ZSM HYM 06637	Germany	22-Jul-2009	SNSB, Zoologische Staatssammlung Muenchen	BOLD:AAO1197	658[0n]
Sapygidae	Sapygina decemguttata	BC ZSM HYM 06579	Germany	07-Jun-2007	SNSB, Zoologische Staatssammlung Muenchen	BOLD:AAO1197	658[0n]
Sapygidae	Sapygina decemguttata	BC ZSM HYM 06560	France	12-Jul-2010	SNSB, Zoologische Staatssammlung Muenchen	BOLD:AAO1197	658[0n]
Sapygidae	Sapygina decemguttata	BC ZSM HYM 05795	Germany	29-Jul-1996	SNSB, Zoologische Staatssammlung Muenchen	BOLD:AAO1197	658[0n]
Sapygidae	Sapygina decemguttata	BC ZSM HYM 05794	Germany	29-Jul-1996	SNSB, Zoologische Staatssammlung Muenchen	BOLD:AAO1197	620[0n]
Sapygidae	Sapygina decemguttata	BC ZSM HYM 05793	Germany	29-Jul-1996	SNSB, Zoologische Staatssammlung Muenchen	BOLD:AAO1197	620[0n]
Sapygidae	Sapygina decemguttata	BC ZSM HYM 05792	Germany	29-Jul-1996	SNSB, Zoologische Staatssammlung Muenchen	BOLD:AAO1197	587[0n]
Sapygidae	Sapygina decemguttata	BC ZSM HYM 16993	Germany	18-Jul-2012	SNSB, Zoologische Staatssammlung Muenchen	BOLD:AAO1197	658[0n]
Sapygidae	Sapygina decemguttata	BC ZSM HYM 07375	Germany	07-Jul-2010	SNSB, Zoologische Staatssammlung Muenchen	BOLD:AAO1197	658[0n]
Sapygidae	Sapygina decemguttata	BC ZSM HYM 07374	Germany	25-Jun-2010	SNSB, Zoologische Staatssammlung Muenchen	BOLD:AAO1197	658[0n]
Scoliidae	Campsomeriella thoracica	BC ZSM HYM 20518	United Arab Emirates	13-Jan-2011	SNSB, Zoologische Staatssammlung Muenchen	BOLD:ADY5639	589[0n]
Scoliidae	Campsomeriella thoracica	BC ZSM HYM 20516	United Arab Emirates	13-Jan-2011	SNSB, Zoologische Staatssammlung Muenchen	BOLD:ADY5639	562[0n]
Scoliidae	Campsomeriella thoracica	BC ZSM HYM 20515	United Arab Emirates	11-Mar-2009	SNSB, Zoologische Staatssammlung Muenchen	BOLD:ADY5639	594[0n]
Scoliidae	Campsomeriella thoracica	BC-ZSM-HYM-30229-G06	Morocco	14-Jun-2017	SNSB, Zoologische Staatssammlung Muenchen	BOLD:ADY5638	607[0n]
Scoliidae	Campsomeriella thoracica	BC-ZSM-HYM-30229-G02	Egypt	01-Apr-2018	SNSB, Zoologische Staatssammlung Muenchen	BOLD:ADY3101	596[0n]
Scoliidae	Campsomeriella thoracica	BC-ZSM-HYM-30229-G01	Egypt	01-Apr-2018	SNSB, Zoologische Staatssammlung Muenchen	BOLD:ADY3101	611[0n]
Scoliidae	Campsomeriella thoracica	BC ZSM HYM 20997	Tunisia	06-Sep-2007	SNSB, Zoologische Staatssammlung Muenchen	BOLD:ADY5638	560[0n]
Scoliidae	Campsomeriella thoracica	BC ZSM HYM 20998	Tunisia	06-Sep-2007	SNSB, Zoologische Staatssammlung Muenchen	BOLD:ADY5638	530[0n]
Scoliidae	Campsomeriella thoracica	BC ZSM HYM 20996	Tunisia	06-Sep-2007	SNSB, Zoologische Staatssammlung Muenchen	BOLD:ADY5638	531[2n]
Scoliidae	Colpa quinquecincta	BC ZSM HYM 17323	Italy	20-Jul-2012	SNSB, Zoologische Staatssammlung Muenchen	BOLD:ACG2231	658[0n]
Scoliidae	Colpa quinquecincta	BC ZSM HYM 17322	Italy	20-Jul-2012	SNSB, Zoologische Staatssammlung Muenchen	BOLD:ACG2231	658[0n]
Scoliidae	Colpa quinquecincta	BC-ZSM-HYM-29769-A03	Italy	27-Jun-2017	SNSB, Zoologische Staatssammlung Muenchen	BOLD:ACG2231	658[0n]

Family	Species	Specimen ID	Country	Collection Date	Depository	BIN	COI-5P
Scoliidae	Colpa quinquecincta	BC-ZSM-HYM-29769-A02	Italy	28-Jun-2017	SNSB, Zoologische Staatssammlung Muenchen	BOLD:ACG2231	658[0n]
Scoliidae	Colpa quinquecincta	BC-ZSM-HYM-29769-A01	Italy	25-Jun-2017	SNSB, Zoologische Staatssammlung Muenchen	BOLD:ACG2231	658[0n]
Scoliidae	Colpa sexmaculata	BC-ZSM-HYM-30229-F12	Croatia	17-Jul-2014	SNSB, Zoologische Staatssammlung Muenchen	BOLD:ADM7341	613[0n]
Scoliidae	Colpa sexmaculata	BC-ZSM-HYM-29768-H07	Italy	03-Jul-2017	SNSB, Zoologische Staatssammlung Muenchen	BOLD:ADM7341	593[0n]
Scoliidae	Colpa sexmaculata	BC-ZSM-HYM-29768-H06	Italy	27-Jun-2017	SNSB, Zoologische Staatssammlung Muenchen	BOLD:ADM7341	620[0n]
Scoliidae	Colpa sexmaculata	BC-ZSM-HYM-29768-H05	Italy	27-Jun-2017	SNSB, Zoologische Staatssammlung Muenchen	BOLD:ADM7341	654[0n]
Scoliidae	Megascolia bidens	BC-ZSM-HYM-30229-H05	Spain	08-Apr-2010	SNSB, Zoologische Staatssammlung Muenchen	BOLD:AEC9507	607[0n]
Scoliidae	Megascolia bidens	BC-ZSM-HYM-30229-H04	Spain	08-Apr-2010	SNSB, Zoologische Staatssammlung Muenchen	BOLD:AEC9507	606[0n]
Scoliidae	Megascolia maculata	BC ZSM HYM 06623	Turkey	01-Aug-2009	SNSB, Zoologische Staatssammlung Muenchen	BOLD:AAV7442	658[0n]
Scoliidae	Megascolia maculata	BC ZSM HYM 06647	Turkey	01-Aug-2009	SNSB, Zoologische Staatssammlung Muenchen	BOLD:AAV7442	658[0n]
Scoliidae	Megascolia maculata	BC-ZSM-HYM-30229-H02	Cyprus	20-Jun-2013	SNSB, Zoologische Staatssammlung Muenchen	BOLD:AAV7442	600[0n]
Scoliidae	Megascolia maculata	BC-ZSM-HYM-29769-H11	Italy	03-Jul-2017	SNSB, Zoologische Staatssammlung Muenchen	BOLD:AAV7442	658[0n]
Scoliidae	Megascolia maculata	BC-ZSM-HYM-29771-H08	Spain	20-Jun-2014	SNSB, Zoologische Staatssammlung Muenchen	BOLD:AAV7442	637[0n]
Scoliidae	Megascolia maculata	BC-ZSM-HYM-29771-H07	Spain	20-Jun-2014	SNSB, Zoologische Staatssammlung Muenchen	BOLD:AAV7442	640[0n]
Scoliidae	Megascolia maculata	BC-ZSM-HYM-29771-H06	Spain	08-Jul-2015	SNSB, Zoologische Staatssammlung Muenchen	BOLD:AAV7442	618[0n]
Scoliidae	Megascolia maculata	BC ZSM HYM 18810	Hungary	13-Aug-2011	SNSB, Zoologische Staatssammlung Muenchen	BOLD:AAV7442	552[0n]
Scoliidae	Micromeriella aureola	BC ZSM HYM 21047	Tunisia	10-Sep-2007	SNSB, Zoologische Staatssammlung Muenchen	BOLD:ACO4425	658[0n]
Scoliidae	Micromeriella aureola	BC ZSM HYM 21046	Tunisia	09-Sep-2007	SNSB, Zoologische Staatssammlung Muenchen	BOLD:ACO4425	658[0n]
Scoliidae	Micromeriella aureola	BC ZSM HYM 21042	Tunisia	10-Sep-2007	SNSB, Zoologische Staatssammlung Muenchen	BOLD:ACO4425	658[0n]
Scoliidae	Micromeriella aureola	BC ZSM HYM 21041	Tunisia	09-Sep-2007	SNSB, Zoologische Staatssammlung Muenchen	BOLD:ACO4425	658[0n]
Scoliidae	Micromeriella hyalina	BC ZSM HYM 21052	United Arab Emirates	18-Mar-2009	SNSB, Zoologische Staatssammlung Muenchen	BOLD:ACO5576	597[0n]
Scoliidae	Micromeriella hyalina	BC-ZSM-HYM-30229-G08	United Arab Emirates	16-Aug-2018	SNSB, Zoologische Staatssammlung Muenchen	BOLD:ACO5284	600[0n]
Scoliidae	Micromeriella hyalina	BC-ZSM-HYM-30229-G07	United Arab Emirates	16-Aug-2018	SNSB, Zoologische Staatssammlung Muenchen	BOLD:ACO5576	588[0n]
Scoliidae	Micromeriella hyalina	BC-ZSM-HYM-30229-G05	Morocco	14-Jun-2016	SNSB, Zoologische Staatssammlung Muenchen	BOLD:ADX9764	575[1n]
Scoliidae	Micromeriella hyalina	BC-ZSM-HYM-30229-G04	Morocco	14-Jun-2015	SNSB, Zoologische Staatssammlung Muenchen	BOLD:ADX9764	607[1n]
Scoliidae	Micromeriella hyalina	BC-ZSM-HYM-30229-G03	Morocco	14-Jun-2015	SNSB, Zoologische Staatssammlung Muenchen	BOLD:ADX9764	574[1n]
Scoliidae	Micromeriella hyalina	BC ZSM HYM 21049	United Arab Emirates	12-Jan-2011	SNSB, Zoologische Staatssammlung Muenchen	BOLD:ACO5284	658[0n]
Scoliidae	Micromeriella hyalina	BC ZSM HYM 21048	United Arab Emirates	11-May-2009	SNSB, Zoologische Staatssammlung Muenchen	BOLD:ACO5577	658[0n]
Scoliidae	Scolia hirta	BC ZSM HYM 14011	Italy	17-Jul-2011	SNSB, Zoologische Staatssammlung Muenchen	BOLD:AAU2351	619[0n]
Scoliidae	Scolia hirta	BC ZSM HYM 21081	Germany	06-Jul-2013	SNSB, Zoologische Staatssammlung Muenchen	BOLD:AAU2351	549[0n]
Scoliidae	Scolia hirta	BC ZSM HYM 10186	Italy	17-Jul-2011	SNSB, Zoologische Staatssammlung Muenchen	BOLD:AAU2351	453[0n]
Scoliidae	Scolia hirta	BC ZSM HYM 06578	Germany	03-Jul-2008	SNSB, Zoologische Staatssammlung Muenchen	BOLD:AAU2351	602[0n]
Scoliidae	Scolia hirta	BC ZSM HYM 06566	Italy	27-Jul-2007	SNSB, Zoologische Staatssammlung Muenchen	BOLD:AAU2351	551[0n]
Scoliidae	Scolia hirta	BC-ZSM-HYM-29768-H04	Italy	03-Jul-2017	SNSB, Zoologische Staatssammlung Muenchen	BOLD:AAU2351	624[0n]
Scoliidae	Scolia hirta	BC-ZSM-HYM-29768-H03	Italy	27-Jun-2017	SNSB, Zoologische Staatssammlung Muenchen	BOLD:AAU2351	658[0n]
Scoliidae	Scolia hirta	BC-ZSM-HYM-29768-H02	Italy	03-Jul-2017	SNSB, Zoologische Staatssammlung Muenchen	BOLD:AAU2351	658[0n]
Scoliidae	Scolia hirta	BC ZSM HYM 15300	Germany	04-Aug-2012	SNSB, Zoologische Staatssammlung Muenchen	BOLD:AAU2351	632[0n]
Scoliidae	Scolia hirta	BC ZSM HYM 15299	Germany	04-Aug-2012	SNSB, Zoologische Staatssammlung Muenchen	BOLD:AAU2351	633[0n]
Scoliidae	Scolia hortorum	BC ZSM HYM 11287	Tunisia	07-Sep-2007	SNSB, Zoologische Staatssammlung Muenchen	BOLD:ACG2158	658[0n]
Scoliidae	Scolia hortorum	BC ZSM HYM 06632	France	12-Jul-2010	SNSB, Zoologische Staatssammlung Muenchen	BOLD:AAU2353	658[1n]
Scoliidae	Scolia miniata	BC ZSM HYM 11282	United Arab Emirates	04-Apr-2009	SNSB, Zoologische Staatssammlung Muenchen	BOLD:ACG2159	582[0n]
Scoliidae	Scolia sexmaculata	BC ZSM HYM 14035	Germany	26-Jun-2010	SNSB, Zoologische Staatssammlung Muenchen	BOLD:AAU2352	658[0n]
Scoliidae	Scolia sexmaculata	BC ZSM HYM 14034	Germany	26-Jun-2010	SNSB, Zoologische Staatssammlung Muenchen	BOLD:AAU2352	658[0n]
Scoliidae	Scolia sexmaculata	BC ZSM HYM 14033	France	10-Jul-2010	SNSB, Zoologische Staatssammlung Muenchen	BOLD:AAU2352	658[1n]
Scoliidae	Scolia sexmaculata	BC ZSM HYM 17324	Italy	20-Jul-2012	SNSB, Zoologische Staatssammlung Muenchen	BOLD:AAU2352	635[0n]
Scoliidae	Scolia sexmaculata	BC ZSM HYM 09857	Germany	22-Jul-2009	SNSB, Zoologische Staatssammlung Muenchen	BOLD:AAU2352	658[0n]
Scoliidae	Scolia sexmaculata	BC ZSM HYM 09856	Germany	22-Jul-2009	SNSB, Zoologische Staatssammlung Muenchen	BOLD:AAU2352	658[0n]

Family	Species	Specimen ID	Country	Collection Date	Depository	BIN	COI-5P
Scoliidae	Scolia sexmaculata	BC ZSM HYM 06620	Germany	26-Jun-2010	SNSB, Zoologische Staatssammlung Muenchen	BOLD:AAU2352	658[0n]
Scoliidae	Scolia sexmaculata	BC ZSM HYM 06608	Germany	26-Jun-2010	SNSB, Zoologische Staatssammlung Muenchen	BOLD:AAU2352	658[0n]
Scoliidae	Scolia sexmaculata	BC ZSM HYM 06596	France	10-Jul-2010	SNSB, Zoologische Staatssammlung Muenchen	BOLD:AAU2352	658[0n]
Scoliidae	Scolia sexmaculata	BC ZSM HYM 06584	France	10-Jul-2010	SNSB, Zoologische Staatssammlung Muenchen	BOLD:AAU2352	658[0n]
Scoliidae	Scolia sexmaculata	BC-ZSM-HYM-29768-H01	Italy	25-Jun-2017	SNSB, Zoologische Staatssammlung Muenchen	BOLD:AAU2352	658[0n]
Scoliidae	Scolia sexmaculata	BC-ZSM-HYM-29768-G12	Italy	03-Jul-2017	SNSB, Zoologische Staatssammlung Muenchen	BOLD:AAU2352	658[0n]
Scoliidae	Scolia sexmaculata	BC-ZSM-HYM-29768-G11	Italy	27-Jun-2017	SNSB, Zoologische Staatssammlung Muenchen	BOLD:AAU2352	658[0n]
Scoliidae	Scolia sexmaculata	BC ZSM HYM 14396	Germany	19-Jun-2012	SNSB, Zoologische Staatssammlung Muenchen	BOLD:AAU2352	658[0n]
Scoliidae	Scolia sexmaculata	BC ZSM HYM 14395	Germany	19-Jun-2012	SNSB, Zoologische Staatssammlung Muenchen	BOLD:AAU2352	658[0n]
Scoliidae	Scolia sexmaculata	BC ZSM HYM 14394	Germany	19-Jun-2012	SNSB, Zoologische Staatssammlung Muenchen	BOLD:AAU2352	658[0n]
Scoliidae	Scolia sexmaculata	BC ZSM HYM 14393	Germany	19-Jun-2012	SNSB, Zoologische Staatssammlung Muenchen	BOLD:AAU2352	658[0n]
Scoliidae	Scolia sexmaculata	BC ZSM HYM 14392	Germany	19-Jun-2012	SNSB, Zoologische Staatssammlung Muenchen	BOLD:AAU2352	658[0n]
Thynnidae	Meria cylindrica	BC ZSM HYM 20029	France	16-Aug-2013	SNSB, Zoologische Staatssammlung Muenchen	BOLD:ACG2533	658[0n]
Thynnidae	Meria cylindrica	BC ZSM HYM 20028	France	19-Jul-2013	SNSB, Zoologische Staatssammlung Muenchen	BOLD:ACG2533	658[0n]
Thynnidae	Meria cylindrica	BC ZSM HYM 20027	France	19-Jul-2013	SNSB, Zoologische Staatssammlung Muenchen	BOLD:ACG2533	658[0n]
Thynnidae	Meria cylindrica	BC ZSM HYM 20026	France	16-Aug-2013	SNSB, Zoologische Staatssammlung Muenchen	BOLD:ACG2533	658[0n]
Thynnidae	Meria cylindrica	BC ZSM HYM 20025	France	18-Aug-2012	SNSB, Zoologische Staatssammlung Muenchen	BOLD:ACG1615	658[0n]
Thynnidae	Meria cylindrica	BC ZSM HYM 20024	France	20-Jul-2013	SNSB, Zoologische Staatssammlung Muenchen	BOLD:ACG1615	658[0n]
Thynnidae	Meria cylindrica	BC-ZSM-HYM-30229-E06	Italy	03-Jul-2017	SNSB, Zoologische Staatssammlung Muenchen	BOLD:ACG1615	592[0n]
Thynnidae	Meria cylindrica	BC ZSM HYM 17463	France	18-Aug-2012	SNSB, Zoologische Staatssammlung Muenchen	BOLD:ACG1615	658[0n]
Thynnidae	Meria cylindrica	BC ZSM HYM 17462	France	18-Aug-2012	SNSB, Zoologische Staatssammlung Muenchen	BOLD:ACG1615	658[0n]
Thynnidae	Meria cylindrica	BC ZSM HYM 17459	France	05-Jul-2008	SNSB, Zoologische Staatssammlung Muenchen	BOLD:ADA9671	658[0n]
Thynnidae	Meria cylindrica	BC ZSM HYM 17458	France	05-Jul-2008	SNSB, Zoologische Staatssammlung Muenchen	BOLD:ACG2533	658[0n]
Thynnidae	Meria cylindrica	BC ZSM HYM 17457	France	28-Jul-2009	SNSB, Zoologische Staatssammlung Muenchen	BOLD:ACG2533	658[0n]
Thynnidae	Meria cylindrica	BC ZSM HYM 17456	France	27-Jul-2009	SNSB, Zoologische Staatssammlung Muenchen	BOLD:ACG2533	658[0n]
Thynnidae	Meria cylindrica	BC ZSM HYM 17455	France	16-Jul-2012	SNSB, Zoologische Staatssammlung Muenchen	BOLD:ACG2533	658[0n]
Thynnidae	Meria cylindrica	BC ZSM HYM 17454	France	08-Aug-2012	SNSB, Zoologische Staatssammlung Muenchen	BOLD:ACG2533	658[0n]
Thynnidae	Meria tripunctata	BC-ZSM-HYM-30229-E09	Italy	27-Jun-2017	SNSB, Zoologische Staatssammlung Muenchen	BOLD:ADX9865	633[0n]
Thynnidae	Meria tripunctata	BC-ZSM-HYM-30229-E08	Italy	27-Jun-2017	SNSB, Zoologische Staatssammlung Muenchen	BOLD:ADX9865	627[0n]
Thynnidae	Meria tripunctata	BC-ZSM-HYM-30229-E07	Italy	27-Jun-2017	SNSB, Zoologische Staatssammlung Muenchen	BOLD:ADX9865	590[0n]
Thynnidae	Meria tripunctata	BC ZSM HYM 17450	Italy	14-Sep-1996	SNSB, Zoologische Staatssammlung Muenchen	BOLD:AAY6941	618[0n]
Thynnidae	Meria tripunctata	BC ZSM HYM 07396	Italy	17-Jun-2009	SNSB, Zoologische Staatssammlung Muenchen	BOLD:AAY6941	658[0n]
Thynnidae	Meria tripunctata	BC ZSM HYM 07395	Italy	17-Jun-2009	SNSB, Zoologische Staatssammlung Muenchen	BOLD:AAY6941	658[0n]
Thynnidae	Methocha articulata	BC-ZSM-HYM-23654-E07	Czech Republic	24-Jun-2008	SNSB, Zoologische Staatssammlung Muenchen	BOLD:AAU3521	658[0n]
Thynnidae	Methocha articulata	BC ZSM HYM 06269	Germany	24-Jul-2008	SNSB, Zoologische Staatssammlung Muenchen	BOLD:AAU3521	658[0n]
Thynnidae	Methocha articulata	BC ZSM HYM 06576	Germany	22-Jul-2009	SNSB, Zoologische Staatssammlung Muenchen	BOLD:AAU3521	635[0n]
Thynnidae	Methocha articulata	BC ZSM HYM 06564	Croatia	27-Aug-2005	SNSB, Zoologische Staatssammlung Muenchen	BOLD:AAU3521	629[0n]
Thynnidae	Poecilotiphia rousseli	BC ZSM HYM 20030	France	20-Jul-2013	SNSB, Zoologische Staatssammlung Muenchen	BOLD:ACM1581	658[0n]
Thynnidae	Poecilotiphia rousseli	BC ZSM HYM 20034	France	29-Aug-2013	SNSB, Zoologische Staatssammlung Muenchen	BOLD:ACM1581	658[0n]
Tiphiidae	Tiphia femorata	BC ZSM HYM 21985	Germany	01-Aug-2014	SNSB, Zoologische Staatssammlung Muenchen	BOLD:AAP1103	632[0n]
Tiphiidae	Tiphia femorata	BC ZSM HYM 15444	Germany	16-Jul-2012	SNSB, Zoologische Staatssammlung Muenchen	BOLD:AAP1103	658[0n]
Tiphiidae	Tiphia femorata	BC ZSM HYM 15442	Germany	02-Jul-2012	SNSB, Zoologische Staatssammlung Muenchen	BOLD:AAY9685	658[0n]
Tiphiidae	Tiphia femorata	BC ZSM HYM 15441	Germany	12-Aug-2012	SNSB, Zoologische Staatssammlung Muenchen	BOLD:AAP1103	658[0n]
Tiphiidae	Tiphia femorata	BC ZSM HYM 15440	Germany	12-Aug-2012	SNSB, Zoologische Staatssammlung Muenchen	BOLD:AAP1103	658[0n]
Tiphiidae	Tiphia femorata	BC ZSM HYM 15439	Germany	01-Aug-2012	SNSB, Zoologische Staatssammlung Muenchen	BOLD:ABA9329	640[1n]
Tiphiidae	Tiphia femorata	BC ZSM HYM 15438	Germany	04-Aug-2012	SNSB, Zoologische Staatssammlung Muenchen	BOLD:AAP1103	658[0n]
Tiphiidae	Tiphia femorata	BC ZSM HYM 15437	Germany	04-Aug-2012	SNSB, Zoologische Staatssammlung Muenchen	BOLD:AAP1102	658[0n]

Family	Species	Specimen ID	Country	Collection Date	Depository	BIN	COI-5P
Tiphiidae	Tiphia femorata	BC ZSM HYM 15435	Germany	04-Aug-2012	SNSB, Zoologische Staatssammlung Muenchen	BOLD:AAP1103	658[0n]
Tiphiidae	Tiphia femorata	BC ZSM HYM 09863	Germany	22-Jul-2009	SNSB, Zoologische Staatssammlung Muenchen	BOLD:AAP1103	658[0n]
Tiphiidae	Tiphia femorata	BC ZSM HYM 09861	Germany	22-Jul-2009	SNSB, Zoologische Staatssammlung Muenchen	BOLD:AAP1102	658[0n]
Tiphiidae	Tiphia femorata	BC ZSM HYM 24561	Hungary	16-Aug-2014	SNSB, Zoologische Staatssammlung Muenchen	BOLD:AAP1102	658[0n]
Tiphiidae	Tiphia femorata	BC ZSM HYM 21902	Germany	07-Jul-2013	SNSB, Zoologische Staatssammlung Muenchen	BOLD:AAY9685	623[0n]
Tiphiidae	Tiphia femorata	BC ZSM HYM 21896	Germany	18-Jul-2012	SNSB, Zoologische Staatssammlung Muenchen	BOLD:AAP1103	626[1n]
Tiphiidae	Tiphia femorata	BC ZSM HYM 21895	Germany	18-Jul-2012	SNSB, Zoologische Staatssammlung Muenchen	BOLD:AAP1103	615[1n]
Tiphiidae	Tiphia femorata	BC ZSM HYM 21894	Germany	12-Jul-2013	SNSB, Zoologische Staatssammlung Muenchen	BOLD:AAY9685	606[0n]
Tiphiidae	Tiphia femorata	BC ZSM HYM 21893	Germany	11-Jul-2013	SNSB, Zoologische Staatssammlung Muenchen	BOLD:AAY9685	633[0n]
Tiphiidae	Tiphia femorata	BC ZSM HYM 21892	Germany	10-Jul-2013	SNSB, Zoologische Staatssammlung Muenchen	BOLD:AAY9685	596[2n]
Tiphiidae	Tiphia femorata	BC ZSM HYM 21891	Germany	09-Jul-2013	SNSB, Zoologische Staatssammlung Muenchen	BOLD:AAY9685	633[0n]
Tiphiidae	Tiphia femorata	BC ZSM HYM 21888	Germany	17-Jul-2007	SNSB, Zoologische Staatssammlung Muenchen	BOLD:AAP1103	626[0n]
Tiphiidae	Tiphia femorata	BC ZSM HYM 21885	Germany	18-Aug-2012	SNSB, Zoologische Staatssammlung Muenchen	BOLD:AAP1102	645[1n]
Tiphiidae	Tiphia femorata	BC ZSM HYM 21884	Germany	01-Aug-2012	SNSB, Zoologische Staatssammlung Muenchen	BOLD:AAP1103	626[0n]
Tiphiidae	Tiphia femorata	BC ZSM HYM 21882	Germany	02-Jul-2012	SNSB, Zoologische Staatssammlung Muenchen	BOLD:AAY9685	633[0n]
Tiphiidae	Tiphia femorata	BC ZSM HYM 21881	Germany	19-Jun-2012	SNSB, Zoologische Staatssammlung Muenchen	BOLD:AAY9685	620[0n]
Tiphiidae	Tiphia femorata	BC ZSM HYM 24266	Hungary	10-Aug-2014	SNSB, Zoologische Staatssammlung Muenchen	BOLD:AAY9685	658[0n]
Tiphiidae	Tiphia femorata	BC ZSM HYM 06351	Germany	27-Jul-2010	SNSB, Zoologische Staatssammlung Muenchen	BOLD:AAP1103	618[1n]
Tiphiidae	Tiphia femorata	BC ZSM HYM 06350	Germany	27-Jul-2010	SNSB, Zoologische Staatssammlung Muenchen	BOLD:AAP1103	658[0n]
Tiphiidae	Tiphia femorata	BC ZSM HYM 06349	Germany	26-Jul-2010	SNSB, Zoologische Staatssammlung Muenchen	BOLD:AAP1102	658[0n]
Tiphiidae	Tiphia femorata	BC ZSM HYM 19942	Germany	02-Aug-2013	SNSB, Zoologische Staatssammlung Muenchen	BOLD:AAP1103	658[0n]
Tiphiidae	Tiphia femorata	BC ZSM HYM 19941	Germany	02-Aug-2013	SNSB, Zoologische Staatssammlung Muenchen	BOLD:AAP1103	658[0n]
Tiphiidae	Tiphia femorata	BC ZSM HYM 19940	Germany	02-Aug-2013	SNSB, Zoologische Staatssammlung Muenchen	BOLD:AAP1103	658[0n]
Tiphiidae	Tiphia femorata	BC ZSM HYM 24676	Germany	07-Jul-2015	SNSB, Zoologische Staatssammlung Muenchen	BOLD:AAP1103	658[0n]
Tiphiidae	Tiphia femorata	BC ZSM HYM 24675	Germany	05-Aug-2015	SNSB, Zoologische Staatssammlung Muenchen	BOLD:AAP1103	658[0n]
Tiphiidae	Tiphia femorata	BC ZSM HYM 24673	Germany	19-Aug-2015	SNSB, Zoologische Staatssammlung Muenchen	BOLD:AAP1103	658[0n]
Tiphiidae	Tiphia femorata	BC ZSM HYM 24672	Germany	19-Aug-2015	SNSB, Zoologische Staatssammlung Muenchen	BOLD:AAY9685	658[0n]
Tiphiidae	Tiphia femorata	BC ZSM HYM 24671	Germany	19-Aug-2015	SNSB, Zoologische Staatssammlung Muenchen	BOLD:AAP1103	658[0n]
Tiphiidae	Tiphia femorata	BC ZSM HYM 24669	Germany	03-Aug-2015	SNSB, Zoologische Staatssammlung Muenchen	BOLD:AAY9685	645[0n]
Tiphiidae	Tiphia femorata	BC ZSM HYM 24668	Germany	05-Aug-2015	SNSB, Zoologische Staatssammlung Muenchen	BOLD:AAP1103	658[0n]
Tiphiidae	Tiphia femorata	BC ZSM HYM 24667	Germany	05-Aug-2015	SNSB, Zoologische Staatssammlung Muenchen	BOLD:AAP1103	658[0n]
Tiphiidae	Tiphia femorata	BC ZSM HYM 24666	Germany	05-Aug-2015	SNSB, Zoologische Staatssammlung Muenchen	BOLD:AAP1103	658[0n]
Tiphiidae	Tiphia femorata	BC ZSM HYM 14099	Germany	22-Jul-2009	SNSB, Zoologische Staatssammlung Muenchen	BOLD:AAP1102	658[0n]
Tiphiidae	Tiphia femorata	BC ZSM HYM 14097	Germany	28-Jul-2005	SNSB, Zoologische Staatssammlung Muenchen	BOLD:AAP1103	658[0n]
Tiphiidae	Tiphia femorata	BC ZSM HYM 14096	Germany	16-Jul-2011	SNSB, Zoologische Staatssammlung Muenchen	BOLD:AAP1103	658[0n]
Tiphiidae	Tiphia femorata	BC ZSM HYM 14095	Germany	16-Jul-2011	SNSB, Zoologische Staatssammlung Muenchen	BOLD:AAP1103	658[0n]
Tiphiidae	Tiphia femorata	BC ZSM HYM 14094	Germany	16-Jul-2011	SNSB, Zoologische Staatssammlung Muenchen	BOLD:AAP1103	658[0n]
Tiphiidae	Tiphia femorata	BC ZSM HYM 14092	Germany	26-Jul-2010	SNSB, Zoologische Staatssammlung Muenchen	BOLD:AAP1102	658[0n]
Tiphiidae	Tiphia femorata	BC ZSM HYM 14091	Germany	26-Jul-2010	SNSB, Zoologische Staatssammlung Muenchen	BOLD:ABA9329	658[0n]
Tiphiidae	Tiphia femorata	BC ZSM HYM 19434	Germany	18-Jul-2012	SNSB, Zoologische Staatssammlung Muenchen	BOLD:AAY9685	658[0n]
Tiphiidae	Tiphia femorata	BC ZSM HYM 19432	Germany	18-Jul-2012	SNSB, Zoologische Staatssammlung Muenchen	BOLD:AAY9685	658[0n]
Tiphiidae	Tiphia femorata	BC ZSM HYM 19431	Germany	18-Jul-2012	SNSB, Zoologische Staatssammlung Muenchen	BOLD:AAY9685	658[0n]
Tiphiidae	Tiphia femorata	BC ZSM HYM 19429	Germany	18-Jul-2012	SNSB, Zoologische Staatssammlung Muenchen	BOLD:AAP1103	658[0n]
Tiphiidae	Tiphia femorata	BC ZSM HYM 19426	Germany	18-Jul-2012	SNSB, Zoologische Staatssammlung Muenchen	BOLD:AAP1103	658[0n]
Tiphiidae	Tiphia femorata	BC ZSM HYM 19425	Germany	18-Jul-2012	SNSB, Zoologische Staatssammlung Muenchen	BOLD:AAP1103	618[0n]
Tiphiidae	Tiphia femorata	BC ZSM HYM 19421	Germany	18-Jul-2012	SNSB, Zoologische Staatssammlung Muenchen	BOLD:AAP1103	578[1n]
Tiphiidae	Tiphia femorata	BC ZSM HYM 19420	Germany	18-Jul-2012	SNSB, Zoologische Staatssammlung Muenchen	BOLD:AAP1103	658[0n]

Family	Species	Specimen ID	Country	Collection Date	Depository	BIN	COI-5P
Tiphiidae	Tiphia femorata	BC ZSM HYM 19419	Germany	18-Jul-2012	SNSB, Zoologische Staatssammlung Muenchen	BOLD:AAP1103	658[0n]
Tiphiidae	Tiphia femorata	BC ZSM HYM 19418	Germany	18-Jul-2012	SNSB, Zoologische Staatssammlung Muenchen	BOLD:AAP1103	658[0n]
Tiphiidae	Tiphia femorata	BC ZSM HYM 19417	Germany	18-Jul-2012	SNSB, Zoologische Staatssammlung Muenchen	BOLD:AAP1103	658[0n]
Tiphiidae	Tiphia femorata	BC ZSM HYM 19416	Germany	18-Jul-2012	SNSB, Zoologische Staatssammlung Muenchen	BOLD:AAP1103	658[0n]
Tiphiidae	Tiphia femorata	BC ZSM HYM 19415	Germany	18-Jul-2012	SNSB, Zoologische Staatssammlung Muenchen	BOLD:AAY9685	658[0n]
Tiphiidae	Tiphia femorata	BC ZSM HYM 19414	Germany	18-Jul-2012	SNSB, Zoologische Staatssammlung Muenchen	BOLD:AAP1103	618[0n]
Tiphiidae	Tiphia femorata	BC ZSM HYM 19413	Germany	18-Jul-2012	SNSB, Zoologische Staatssammlung Muenchen	BOLD:AAP1103	658[0n]
Tiphiidae	Tiphia femorata	BC ZSM HYM 19412	Germany	18-Jul-2012	SNSB, Zoologische Staatssammlung Muenchen	BOLD:AAP1103	658[0n]
Tiphiidae	Tiphia femorata	BC ZSM HYM 19410	Germany	18-Jul-2012	SNSB, Zoologische Staatssammlung Muenchen	BOLD:AAP1103	658[0n]
Tiphiidae	Tiphia femorata	BC ZSM HYM 19409	Germany	18-Jul-2012	SNSB, Zoologische Staatssammlung Muenchen	BOLD:AAP1103	658[0n]
Tiphiidae	Tiphia femorata	BC ZSM HYM 17445	Germany	15-Jul-2012	SNSB, Zoologische Staatssammlung Muenchen	BOLD:AAP1103	658[0n]
Tiphiidae	Tiphia femorata	BC ZSM HYM 17444	Germany	15-Jul-2012	SNSB, Zoologische Staatssammlung Muenchen	BOLD:AAP1103	630[0n]
Tiphiidae	Tiphia femorata	BC ZSM HYM 17443	Germany	15-Jul-2012	SNSB, Zoologische Staatssammlung Muenchen	BOLD:AAP1103	658[0n]
Tiphiidae	Tiphia femorata	BC ZSM HYM 17442	Germany	15-Jul-2012	SNSB, Zoologische Staatssammlung Muenchen	BOLD:AAP1103	658[0n]
Tiphiidae	Tiphia femorata	BC ZSM HYM 17441	Germany	15-Jul-2012	SNSB, Zoologische Staatssammlung Muenchen	BOLD:AAP1103	658[0n]
Tiphiidae	Tiphia femorata	BC ZSM HYM 17440	Germany	21-Aug-2012	SNSB, Zoologische Staatssammlung Muenchen	BOLD:AAP1103	658[0n]
Tiphiidae	Tiphia femorata	BC ZSM HYM 17439	Italy	20-Jul-2012	SNSB, Zoologische Staatssammlung Muenchen	BOLD:ABA9329	630[0n]
Tiphiidae	Tiphia femorata	BC ZSM HYM 17438	Italy	20-Jul-2012	SNSB, Zoologische Staatssammlung Muenchen	BOLD:ABA9329	630[0n]
Tiphiidae	Tiphia femorata	BC-ZSM-HYM-30223-E10	Romania	18-Aug-2018	SNSB, Zoologische Staatssammlung Muenchen	BOLD:AAP1103	658[0n]
Tiphiidae	Tiphia femorata	BC-ZSM-HYM-30223-E09	Romania	18-Aug-2018	SNSB, Zoologische Staatssammlung Muenchen	BOLD:AAP1103	658[0n]
Tiphiidae	Tiphia femorata	BC-ZSM-HYM-30223-E08	Romania	18-Aug-2018	SNSB, Zoologische Staatssammlung Muenchen	BOLD:AAP1103	612[1n]
Tiphiidae	Tiphia femorata	BC-ZSM-HYM-30223-E07	Romania	18-Aug-2018	SNSB, Zoologische Staatssammlung Muenchen	BOLD:AAP1103	658[0n]
Tiphiidae	Tiphia femorata	BC-ZSM-HYM-30223-E06	Romania	18-Aug-2018	SNSB, Zoologische Staatssammlung Muenchen	BOLD:AAP1103	658[0n]
Tiphiidae	Tiphia femorata	BC-ZSM-HYM-30223-E05	France	15-Aug-2018	SNSB, Zoologische Staatssammlung Muenchen	BOLD:ADV9902	635[0n]
Tiphiidae	Tiphia femorata	BC-ZSM-HYM-30223-E04	France	15-Aug-2018	SNSB, Zoologische Staatssammlung Muenchen	BOLD:ADV9902	658[0n]
Tiphiidae	Tiphia femorata	BC ZSM HYM 10193	Germany	09-Jul-2011	SNSB, Zoologische Staatssammlung Muenchen	BOLD:ABA9329	631[0n]
Tiphiidae	Tiphia femorata	BC ZSM HYM 10192	Germany	16-Jul-2011	SNSB, Zoologische Staatssammlung Muenchen	BOLD:AAP1103	658[0n]
Tiphiidae	Tiphia femorata	BC ZSM HYM 10191	Germany	16-Jul-2011	SNSB, Zoologische Staatssammlung Muenchen	BOLD:AAP1103	658[0n]
Tiphiidae	Tiphia femorata	BC ZSM HYM 10190	Germany	16-Jul-2011	SNSB, Zoologische Staatssammlung Muenchen	BOLD:AAP1103	658[0n]
Tiphiidae	Tiphia femorata	BC ZSM HYM 10189	Germany	09-Jul-2011	SNSB, Zoologische Staatssammlung Muenchen	BOLD:ABA9329	631[0n]
Tiphiidae	Tiphia femorata	BC ZSM HYM 10188	Germany	09-Jul-2011	SNSB, Zoologische Staatssammlung Muenchen	BOLD:AAP1103	658[0n]
Tiphiidae	Tiphia femorata	BC ZSM HYM 09862	Germany	22-Jul-2009	SNSB, Zoologische Staatssammlung Muenchen	BOLD:AAP1102	658[0n]
Tiphiidae	Tiphia femorata	BC ZSM HYM 19406	Germany	18-Jul-2012	SNSB, Zoologische Staatssammlung Muenchen	BOLD:ABA9329	658[0n]
Tiphiidae	Tiphia femorata	BC ZSM HYM 07381	Germany	26-Jul-2010	SNSB, Zoologische Staatssammlung Muenchen	BOLD:AAP1102	658[0n]
Tiphiidae	Tiphia femorata	BC ZSM HYM 07380	Germany	26-Jul-2010	SNSB, Zoologische Staatssammlung Muenchen	BOLD:AAP1102	658[0n]
Tiphiidae	Tiphia femorata	BC ZSM HYM 08165	Germany	15-Jul-2009	SNSB, Zoologische Staatssammlung Muenchen	BOLD:AAY9685	621[0n]
Tiphiidae	Tiphia femorata	BC ZSM HYM 08164	Germany	07-Jun-2008	SNSB, Zoologische Staatssammlung Muenchen	BOLD:AAY9685	658[0n]
Tiphiidae	Tiphia minuta	BC ZSM HYM 06644	Germany	01-Jul-2008	SNSB, Zoologische Staatssammlung Muenchen	BOLD:AAU3783	658[0n]
Tiphiidae	Tiphia minuta	BC ZSM HYM 15171	Germany	04-Jul-2008	SNSB, Zoologische Staatssammlung Muenchen	BOLD:AAU3783	658[0n]
Tiphiidae	Tiphia minuta	BC ZSM HYM 06627	Germany	16-Jun-2008	SNSB, Zoologische Staatssammlung Muenchen	BOLD:AAU3783	658[0n]
Tiphiidae	Tiphia minuta	BC ZSM HYM 06615	Germany	01-Jul-2008	SNSB, Zoologische Staatssammlung Muenchen	BOLD:AAU3783	658[0n]
Tiphiidae	Tiphia minuta	BC ZSM HYM 08163	Germany	19-May-2007	SNSB, Zoologische Staatssammlung Muenchen	BOLD:AAU3783	658[0n]
Tiphiidae	Tiphia unicolor	BC ZSM HYM 15446	Germany	16-Jul-2011	SNSB, Zoologische Staatssammlung Muenchen	BOLD:AAU2938	658[0n]
Tiphiidae	Tiphia unicolor	BC ZSM HYM 16987	Germany	18-Jul-2012	SNSB, Zoologische Staatssammlung Muenchen	BOLD:AAU2938	535[0n]
Tiphiidae	Tiphia unicolor	BC ZSM HYM 13363	Germany	04-Jul-2009	SNSB, Zoologische Staatssammlung Muenchen	BOLD:AAU2938	658[0n]
Tiphiidae	Tiphia unicolor	BC ZSM HYM 13361	Germany	04-Jul-2009	SNSB, Zoologische Staatssammlung Muenchen	BOLD:AAU2938	658[0n]
Tiphiidae	Tiphia unicolor	BC ZSM HYM 13360	Germany	22-Jul-2009	SNSB, Zoologische Staatssammlung Muenchen	BOLD:AAU2938	658[0n]

Family	Species	Specimen ID	Country	Collection Date	Depository	BIN	COI-5P
Tiphiidae	Tiphia unicolor	BC ZSM HYM 14098	Germany	22-Jul-2009	SNSB, Zoologische Staatssammlung Muenchen	BOLD:AAU2938	658[0n]
Tiphiidae	Tiphia unicolor	BC ZSM HYM 17001	Germany	18-Jul-2012	SNSB, Zoologische Staatssammlung Muenchen	BOLD:AAU2938	658[0n]
Tiphiidae	Tiphia unicolor	BC ZSM HYM 16988	Germany	18-Jul-2012	SNSB, Zoologische Staatssammlung Muenchen	BOLD:AAU2938	658[0n]
Tiphiidae	Tiphia unicolor	BC ZSM HYM 19408	Germany	18-Jul-2012	SNSB, Zoologische Staatssammlung Muenchen	BOLD:AAU2938	658[0n]
Tiphiidae	Tiphia unicolor	BC ZSM HYM 17436	Germany	07-Jul-2010	SNSB, Zoologische Staatssammlung Muenchen	BOLD:AAU2938	658[0n]
Tiphiidae	Tiphia unicolor	BC ZSM HYM 09859	Germany	27-Jun-2011	SNSB, Zoologische Staatssammlung Muenchen	BOLD:AAU2938	658[0n]
Tiphiidae	Tiphia unicolor	BC ZSM HYM 09858	Germany	27-Jun-2011	SNSB, Zoologische Staatssammlung Muenchen	BOLD:AAU2938	658[0n]
Tiphiidae	Tiphia unicolor	BC ZSM HYM 06597	Italy	09-Jul-2006	SNSB, Zoologische Staatssammlung Muenchen	BOLD:AAU2938	658[0n]
Tiphiidae	Tiphia unicolor	BC ZSM HYM 06585	Italy	09-Jul-2006	SNSB, Zoologische Staatssammlung Muenchen	BOLD:AAU2938	658[0n]
Tiphiidae	Tiphia unicolor	BC ZSM HYM 06573	Germany	15-Jul-2008	SNSB, Zoologische Staatssammlung Muenchen	BOLD:AAU2938	658[0n]
Tiphiidae	Tiphia unicolor	BC ZSM HYM 06561	Germany	15-Jul-2008	SNSB, Zoologische Staatssammlung Muenchen	BOLD:AAU2938	658[0n]
Tiphiidae	Tiphia unicolor	BC ZSM HYM 08199	Germany	15-Jul-2008	SNSB, Zoologische Staatssammlung Muenchen	BOLD:AAU2938	658[0n]
Tiphiidae	Tiphia unicolor	BC ZSM HYM 08198	Germany	06-Jul-2004	SNSB, Zoologische Staatssammlung Muenchen	BOLD:AAU2938	658[0n]
Tiphiidae	Tiphia unicolor	BC ZSM HYM 08197	Germany	26-Jul-2007	SNSB, Zoologische Staatssammlung Muenchen	BOLD:AAU2938	658[0n]
Tiphiidae	Tiphia unicolor	BC ZSM HYM 14391	Germany	02-Jul-2012	SNSB, Zoologische Staatssammlung Muenchen	BOLD:AAU2938	658[0n]
Tiphiidae	Tiphia unicolor	BC ZSM HYM 14390	Germany	30-Jun-2012	SNSB, Zoologische Staatssammlung Muenchen	BOLD:AAU2938	658[0n]
Tiphiidae	Tiphia villosa	BC ZSM HYM 06645	Germany	27-Apr-2008	SNSB, Zoologische Staatssammlung Muenchen	BOLD:AAU2941	658[0n]
Tiphiidae	Tiphia villosa	BC ZSM HYM 06633	Germany	27-Apr-2008	SNSB, Zoologische Staatssammlung Muenchen	BOLD:AAU2941	621[0n]
Tiphiidae	Tiphia villosa	BC ZSM HYM 06621	Germany	27-Apr-2008	SNSB, Zoologische Staatssammlung Muenchen	BOLD:AAU2941	658[0n]
Tiphiidae	Tiphia villosa	BC ZSM HYM 06609	Germany	27-Apr-2008	SNSB, Zoologische Staatssammlung Muenchen	BOLD:AAU2941	658[0n]
Vespidae	Alastor atropos	BC ZSM HYM 17480	Germany	18-Jul-2012	SNSB, Zoologische Staatssammlung Muenchen	BOLD:ACG1916	658[0n]
Vespidae	Alastor atropos	BC ZSM HYM 17200	Germany	18-Jul-2012	SNSB, Zoologische Staatssammlung Muenchen	BOLD:ACG1916	630[0n]
Vespidae	Alastor biegelebeni	BC-ZSM-HYM-23654-D03	Czech Republic	30-May-2005	SNSB, Zoologische Staatssammlung Muenchen	BOLD:ADH3858	658[0n]
Vespidae	Allodynerus delphinalis	BC ZSM HYM 24664	Germany	05-Aug-2015	SNSB, Zoologische Staatssammlung Muenchen	BOLD:AAM4849	658[0n]
Vespidae	Allodynerus delphinalis	BC ZSM HYM 17198	Italy	20-Jul-2012	SNSB, Zoologische Staatssammlung Muenchen	BOLD:AAM4849	658[0n]
Vespidae	Allodynerus delphinalis	BC ZSM HYM 04783	Germany	16-Sep-2008	SNSB, Zoologische Staatssammlung Muenchen	BOLD:AAM4849	658[0n]
Vespidae	Allodynerus delphinalis	BC ZSM HYM 04782	Germany	12-Aug-2003	SNSB, Zoologische Staatssammlung Muenchen	BOLD:AAM4849	658[0n]
Vespidae	Allodynerus rossii	BC ZSM HYM 04787	Germany	01-Jul-2008	SNSB, Zoologische Staatssammlung Muenchen	BOLD:AAM3835	658[0n]
Vespidae	Allodynerus rossii	BC ZSM HYM 04785	Germany	01-Jul-2008	SNSB, Zoologische Staatssammlung Muenchen	BOLD:AAM3835	658[0n]
Vespidae	Allodynerus rossii	BC ZSM HYM 04784	Germany	01-Jun-2008	SNSB, Zoologische Staatssammlung Muenchen	BOLD:AAM3835	653[0n]
Vespidae	Ancistrocerus antilope	BC ZSM HYM 04753	Germany	01-Aug-2008	SNSB, Zoologische Staatssammlung Muenchen	BOLD:AAM2254	634[0n]
Vespidae	Ancistrocerus antilope	BC ZSM HYM 04752	Germany	01-Sep-2008	SNSB, Zoologische Staatssammlung Muenchen	BOLD:AAM2254	658[0n]
Vespidae	Ancistrocerus antilope	BC ZSM HYM 04751	Germany	10-Jun-2008	SNSB, Zoologische Staatssammlung Muenchen	BOLD:AAM2254	658[0n]
Vespidae	Ancistrocerus antilope	BC ZSM HYM 15265	Germany	04-Aug-2012	SNSB, Zoologische Staatssammlung Muenchen	BOLD:AAM2254	658[0n]
Vespidae	Ancistrocerus antilope	BC ZSM HYM 04754	Germany	10-Jun-2006	SNSB, Zoologische Staatssammlung Muenchen	BOLD:AAM2254	658[1n]
Vespidae	Ancistrocerus auctus	BC ZSM HYM 04757	Italy	09-Jul-2006	SNSB, Zoologische Staatssammlung Muenchen	BOLD:AAM4699	658[0n]
Vespidae	Ancistrocerus auctus	BC ZSM HYM 04756	Italy	25-Jul-1999	SNSB, Zoologische Staatssammlung Muenchen	BOLD:AAM4699	658[0n]
Vespidae	Ancistrocerus auctus	BC ZSM HYM 04755	Italy	25-Jul-1999	SNSB, Zoologische Staatssammlung Muenchen	BOLD:AAM4699	619[0n]
Vespidae	Ancistrocerus biphaleratus	BC ZSM HYM 24032	Italy	05-May-2014	SNSB, Zoologische Staatssammlung Muenchen	BOLD:ACV7686	658[0n]
Vespidae	Ancistrocerus claripennis	BC ZSM HYM 13985	Germany	10-Jun-2005	SNSB, Zoologische Staatssammlung Muenchen	BOLD:AAM3573	658[0n]
Vespidae	Ancistrocerus claripennis	BC ZSM HYM 15261	Germany	01-Aug-2012	SNSB, Zoologische Staatssammlung Muenchen	BOLD:AAM3573	658[0n]
Vespidae	Ancistrocerus claripennis	BC ZSM HYM 04764	Italy	11-Jul-2009	SNSB, Zoologische Staatssammlung Muenchen	BOLD:AAM3573	658[0n]
Vespidae	Ancistrocerus claripennis	BC ZSM HYM 04762	Italy	11-Jul-2009	SNSB, Zoologische Staatssammlung Muenchen	BOLD:AAM3573	658[0n]
Vespidae	Ancistrocerus claripennis	BC ZSM HYM 04761	Italy	11-Jul-2009	SNSB, Zoologische Staatssammlung Muenchen	BOLD:AAM3573	658[0n]
Vespidae	Ancistrocerus claripennis	BC ZSM HYM 04760	Germany	30-Jul-2008	SNSB, Zoologische Staatssammlung Muenchen	BOLD:AAM3573	658[0n]
Vespidae	Ancistrocerus dusmetiolus	BC ZSM HYM 20106	Germany	28-May-2013	SNSB, Zoologische Staatssammlung Muenchen	BOLD:ACP4668	664[0n]
Vespidae	Ancistrocerus gazella	BC ZSM HYM 13090	Germany	19-Jul-2011	SNSB, Zoologische Staatssammlung Muenchen	BOLD:AAF4164	658[0n]

Family	Species	Specimen ID	Country	Collection Date	Depository	BIN	COI-5P
Vespidae	Ancistrocerus gazella	BC ZSM HYM 17378	Italy	20-Jul-2012	SNSB, Zoologische Staatssammlung Muenchen	BOLD:AAF4164	658[0n]
Vespidae	Ancistrocerus gazella	BC ZSM HYM 17197	Germany	18-Jul-2012	SNSB, Zoologische Staatssammlung Muenchen	BOLD:AAF4164	658[0n]
Vespidae	Ancistrocerus gazella	BC ZSM HYM 13089	Germany	07-Jul-2011	SNSB, Zoologische Staatssammlung Muenchen	BOLD:AAF4164	658[0n]
Vespidae	Ancistrocerus gazella	BC ZSM HYM 04766	Italy	08-Jun-2007	SNSB, Zoologische Staatssammlung Muenchen	BOLD:AAF4164	636[0n]
Vespidae	Ancistrocerus gazella	BC ZSM HYM 04765	Germany	10-May-2007	SNSB, Zoologische Staatssammlung Muenchen	BOLD:AAF4164	634[0n]
Vespidae	Ancistrocerus gazella	BC ZSM HYM 04763	Germany	01-Jul-2006	SNSB, Zoologische Staatssammlung Muenchen	BOLD:AAF4164	658[0n]
Vespidae	Ancistrocerus gazella	BC ZSM HYM 04759	Germany	24-Jul-2008	SNSB, Zoologische Staatssammlung Muenchen	BOLD:AAF4164	658[0n]
Vespidae	Ancistrocerus ichneumonideus	BC ZSM HYM 19922	Germany	09-Jun-2013	SNSB, Zoologische Staatssammlung Muenchen	BOLD:ACC4131	658[0n]
Vespidae	Ancistrocerus ichneumonideus	BC ZSM HYM 13208	Germany	02-Jul-2007	SNSB, Zoologische Staatssammlung Muenchen	BOLD:ACC4131	658[0n]
Vespidae	Ancistrocerus longispinosus	GBOL18848	Cyprus	20-Jun-2013	SNSB, Zoologische Staatssammlung Muenchen	BOLD:ACV8574	658[0n]
Vespidae	Ancistrocerus longispinosus	GBOL18846	Cyprus	20-Jun-2013	SNSB, Zoologische Staatssammlung Muenchen	BOLD:ACV8574	644[0n]
Vespidae	Ancistrocerus longispinosus	BC ZSM HYM 24034	Switzerland	13-Aug-2013	SNSB, Zoologische Staatssammlung Muenchen	BOLD:ACV8574	658[0n]
Vespidae	Ancistrocerus nigricornis	BC ZSM HYM 17377	Germany	18-Jul-2012	SNSB, Zoologische Staatssammlung Muenchen	BOLD:AAM3899	658[0n]
Vespidae	Ancistrocerus nigricornis	BC ZSM HYM 14027	Germany	19-Aug-2006	SNSB, Zoologische Staatssammlung Muenchen	BOLD:AAM3899	658[0n]
Vespidae	Ancistrocerus nigricornis	BC ZSM HYM 14026	Germany	19-Aug-2006	SNSB, Zoologische Staatssammlung Muenchen	BOLD:AAM3899	658[0n]
Vespidae	Ancistrocerus nigricornis	BC ZSM HYM 14025	Germany	19-Aug-2006	SNSB, Zoologische Staatssammlung Muenchen	BOLD:AAM3899	658[0n]
Vespidae	Ancistrocerus nigricornis	BC ZSM HYM 14024	Germany	09-Jul-2011	SNSB, Zoologische Staatssammlung Muenchen	BOLD:AAM3899	658[0n]
Vespidae	Ancistrocerus nigricornis	BC ZSM HYM 14023	Germany	16-Jul-2011	SNSB, Zoologische Staatssammlung Muenchen	BOLD:AAM3899	658[0n]
Vespidae	Ancistrocerus nigricornis	BC ZSM HYM 17376	Germany	18-Jul-2012	SNSB, Zoologische Staatssammlung Muenchen	BOLD:AAM3899	658[0n]
Vespidae	Ancistrocerus nigricornis	BC ZSM HYM 17375	Germany	18-Jul-2012	SNSB, Zoologische Staatssammlung Muenchen	BOLD:AAM3899	658[0n]
Vespidae	Ancistrocerus nigricornis	BC ZSM HYM 15260	Germany	04-Aug-2012	SNSB, Zoologische Staatssammlung Muenchen	BOLD:AAM3899	658[0n]
Vespidae	Ancistrocerus nigricornis	BC ZSM HYM 15259	Germany	04-Aug-2012	SNSB, Zoologische Staatssammlung Muenchen	BOLD:AAM3899	658[0n]
Vespidae	Ancistrocerus nigricornis	BC ZSM HYM 15258	Germany	18-Aug-2012	SNSB, Zoologische Staatssammlung Muenchen	BOLD:AAM3899	658[0n]
Vespidae	Ancistrocerus nigricornis	BC ZSM HYM 15257	Germany	28-Aug-2012	SNSB, Zoologische Staatssammlung Muenchen	BOLD:AAM3899	658[0n]
Vespidae	Ancistrocerus nigricornis	GBOL04176	Germany	23-Mar-2012	SNSB, Zoologische Staatssammlung Muenchen	BOLD:AAM3899	658[0n]
Vespidae	Ancistrocerus nigricornis	BC ZSM HYM 04770	Germany	12-May-2008	SNSB, Zoologische Staatssammlung Muenchen	BOLD:AAM3899	658[0n]
Vespidae	Ancistrocerus nigricornis	BC ZSM HYM 04769	Germany	01-Oct-2008	SNSB, Zoologische Staatssammlung Muenchen	BOLD:AAM3899	658[0n]
Vespidae	Ancistrocerus nigricornis	BC ZSM HYM 04768	Germany	05-Aug-2007	SNSB, Zoologische Staatssammlung Muenchen	BOLD:AAM3899	658[0n]
Vespidae	Ancistrocerus nigricornis	BC ZSM HYM 04767	Germany	22-Jul-2009	SNSB, Zoologische Staatssammlung Muenchen	BOLD:AAM3899	658[0n]
Vespidae	Ancistrocerus nigricornis	BC ZSM HYM 10217	Germany	09-Jul-2011	SNSB, Zoologische Staatssammlung Muenchen	BOLD:AAM3899	658[0n]
Vespidae	Ancistrocerus nigricornis	GBOL04177	Germany	23-Mar-2012	SNSB, Zoologische Staatssammlung Muenchen	BOLD:AAM3899	658[0n]
Vespidae	Ancistrocerus oviventris	BC ZSM HYM 13984	Germany	28-Jul-2006	SNSB, Zoologische Staatssammlung Muenchen	BOLD:AAJ2022	658[0n]
Vespidae	Ancistrocerus oviventris	BC ZSM HYM 13982	Germany	28-Jul-2006	SNSB, Zoologische Staatssammlung Muenchen	BOLD:AAJ2022	658[0n]
Vespidae	Ancistrocerus oviventris	BC ZSM HYM 17207	Italy	20-Jul-2012	SNSB, Zoologische Staatssammlung Muenchen	BOLD:AAJ2022	658[0n]
Vespidae	Ancistrocerus oviventris	BC ZSM HYM 17206	Italy	20-Jul-2012	SNSB, Zoologische Staatssammlung Muenchen	BOLD:AAJ2022	658[0n]
Vespidae	Ancistrocerus oviventris	BC ZSM HYM 17205	Italy	20-Jul-2012	SNSB, Zoologische Staatssammlung Muenchen	BOLD:AAJ2022	658[0n]
Vespidae	Ancistrocerus oviventris	BC ZSM HYM 04775	Italy	11-Jul-2009	SNSB, Zoologische Staatssammlung Muenchen	BOLD:AAJ2022	658[0n]
Vespidae	Ancistrocerus oviventris	BC ZSM HYM 04774	Italy	17-Jun-2009	SNSB, Zoologische Staatssammlung Muenchen	BOLD:AAJ2022	658[0n]
Vespidae	Ancistrocerus oviventris	BC ZSM HYM 04773	Italy	27-Jul-2007	SNSB, Zoologische Staatssammlung Muenchen	BOLD:AAJ2022	634[0n]
Vespidae	Ancistrocerus oviventris	BC ZSM HYM 04772	Germany	21-May-2007	SNSB, Zoologische Staatssammlung Muenchen	BOLD:AAJ2022	658[0n]
Vespidae	Ancistrocerus oviventris	BC ZSM HYM 08262	Germany	19-Apr-2011	SNSB, Zoologische Staatssammlung Muenchen	BOLD:AAJ2022	658[0n]
Vespidae	Ancistrocerus oviventris	BC ZSM HYM 08261	Germany	19-Apr-2011	SNSB, Zoologische Staatssammlung Muenchen	BOLD:AAJ2022	658[0n]
Vespidae	Ancistrocerus oviventris	BC ZSM HYM 08260	Germany	19-Apr-2011	SNSB, Zoologische Staatssammlung Muenchen	BOLD:AAJ2022	658[0n]
Vespidae	Ancistrocerus oviventris	BC ZSM HYM 08259	Germany	19-Apr-2011	SNSB, Zoologische Staatssammlung Muenchen	BOLD:AAJ2022	658[0n]
Vespidae	Ancistrocerus parietinus	BC ZSM HYM 04771	Poland	12-May-2008	SNSB, Zoologische Staatssammlung Muenchen	BOLD:AAM4868	658[0n]
Vespidae	Ancistrocerus parietum	BC ZSM HYM 13088	Germany	19-Jul-2011	SNSB, Zoologische Staatssammlung Muenchen	BOLD:AAM4869	658[0n]
Vespidae	Ancistrocerus parietum	BC ZSM HYM 04776	Germany	05-Aug-2007	SNSB, Zoologische Staatssammlung Muenchen	BOLD:AAM4869	658[0n]

Family	Species	Specimen ID	Country	Collection Date	Depository	BIN	COI-5P
Vespidae	Ancistrocerus renimacula	BC ZSM HYM 04758	France	12-Jul-2009	SNSB, Zoologische Staatssammlung Muenchen	BOLD:AAM4859	658[0n]
Vespidae	Ancistrocerus trifasciatus	BC ZSM HYM 13988	Germany	28-Jul-2005	SNSB, Zoologische Staatssammlung Muenchen	BOLD:AAM3937	658[0n]
Vespidae	Ancistrocerus trifasciatus	BC ZSM HYM 17204	Germany	18-Jul-2012	SNSB, Zoologische Staatssammlung Muenchen	BOLD:AAM3937	658[0n]
Vespidae	Ancistrocerus trifasciatus	BC ZSM HYM 04780	Austria	06-Aug-2007	SNSB, Zoologische Staatssammlung Muenchen	BOLD:AAM3937	658[0n]
Vespidae	Ancistrocerus trifasciatus	BC ZSM HYM 04779	Germany	02-Jul-2008	SNSB, Zoologische Staatssammlung Muenchen	BOLD:AAM3937	658[0n]
Vespidae	Ancistrocerus trifasciatus	BC ZSM HYM 04778	Germany	01-Jul-2008	SNSB, Zoologische Staatssammlung Muenchen	BOLD:AAM3937	658[0n]
Vespidae	Ancistrocerus trifasciatus	BC ZSM HYM 04777	Germany	01-Sep-2008	SNSB, Zoologische Staatssammlung Muenchen	BOLD:AAM3937	658[0n]
Vespidae	Ancistrocerus trifasciatus	BC ZSM HYM 10216	Italy	17-Jul-2011	SNSB, Zoologische Staatssammlung Muenchen	BOLD:AAM3937	658[0n]
Vespidae	Antepipona deflenda	GBOL18868	Cyprus	20-Jun-2013	SNSB, Zoologische Staatssammlung Muenchen	BOLD:ADL0396	658[0n]
Vespidae	Antepipona deflenda	GBOL18867	Cyprus	20-Jun-2013	SNSB, Zoologische Staatssammlung Muenchen	BOLD:ADL0396	658[0n]
Vespidae	Antepipona orbitalis	BC ZSM HYM 13135	Hungary	09-May-2011	SNSB, Zoologische Staatssammlung Muenchen	BOLD:ACC2791	658[0n]
Vespidae	Antepipona orbitalis	BC ZSM HYM 13134	Hungary	09-May-2011	SNSB, Zoologische Staatssammlung Muenchen	BOLD:ACC2791	658[0n]
Vespidae	Antepipona orbitalis	BC ZSM HYM 13133	Hungary	09-May-2011	SNSB, Zoologische Staatssammlung Muenchen	BOLD:ACC2791	658[0n]
Vespidae	Antepipona orbitalis	BC-ZSM-HYM-23654-E01	Hungary	05-Jul-2006	SNSB, Zoologische Staatssammlung Muenchen	BOLD:ACC2791	658[0n]
Vespidae	Celonites abbreviatus	BC ZSM HYM 22351	Switzerland	11-Aug-2013	SNSB, Zoologische Staatssammlung Muenchen	BOLD:AAP2382	658[0n]
Vespidae	Celonites abbreviatus	BC ZSM HYM 22350	Switzerland	12-Aug-2013	SNSB, Zoologische Staatssammlung Muenchen	BOLD:AAP2382	658[0n]
Vespidae	Celonites abbreviatus	BC ZSM HYM 22349	Switzerland	05-Aug-2013	SNSB, Zoologische Staatssammlung Muenchen	BOLD:AAP2382	658[0n]
Vespidae	Celonites abbreviatus	BC ZSM HYM 05056	Italy	11-Jul-2009	SNSB, Zoologische Staatssammlung Muenchen	BOLD:AAP2383	658[0n]
Vespidae	Celonites abbreviatus	BC ZSM HYM 05055	Italy	17-Jun-2009	SNSB, Zoologische Staatssammlung Muenchen	BOLD:AAP2382	658[1n]
Vespidae	Celonites abbreviatus	BC ZSM HYM 05054	Germany	01-Jul-2008	SNSB, Zoologische Staatssammlung Muenchen	BOLD:AAP2382	658[0n]
Vespidae	Celonites abbreviatus	BC ZSM HYM 05053	Austria	06-Aug-2007	SNSB, Zoologische Staatssammlung Muenchen	BOLD:AAP2382	658[0n]
Vespidae	Celonites abbreviatus	BC ZSM HYM 17373	Italy	20-Jul-2012	SNSB, Zoologische Staatssammlung Muenchen	BOLD:AAP2383	658[0n]
Vespidae	Celonites abbreviatus	BC ZSM HYM 17374	Italy	20-Jul-2012	SNSB, Zoologische Staatssammlung Muenchen	BOLD:AAP2383	658[0n]
Vespidae	Celonites abbreviatus	BC ZSM HYM 10209	Italy	17-Jul-2011	SNSB, Zoologische Staatssammlung Muenchen	BOLD:AAP2382	658[0n]
Vespidae	Celonites mayeti	BC ZSM HYM 16245	France	12-Jul-2009	SNSB, Zoologische Staatssammlung Muenchen	BOLD:ACG0764	658[0n]
Vespidae	Celonites rugiceps	20191113AIMSEQ-005	Greece	21-Jun-2019	Research Collection of Volker Mauss	BOLD:ACX5359	622[0n]
Vespidae	Celonites rugiceps	20191113AIMSEQ-004	Greece	20-Jun-2019	Research Collection of Volker Mauss	BOLD:ACX5359	624[0n]
Vespidae	Ceramius tuberculifer	BC ZSM HYM 24587	France	16-Jul-2010	SNSB, Zoologische Staatssammlung Muenchen	BOLD:ACX4794	658[0n]
Vespidae	Delta unguiculatum	BC ZSM HYM 04803	Italy	09-Jul-2006	SNSB, Zoologische Staatssammlung Muenchen	BOLD:AAN1374	658[0n]
Vespidae	Discoelius dufourii	BC ZSM HYM 04806	Germany	12-Aug-2003	SNSB, Zoologische Staatssammlung Muenchen	BOLD:AAN1415	658[0n]
Vespidae	Discoelius zonalis	BC ZSM HYM 04807	Germany	31-May-1997	SNSB, Zoologische Staatssammlung Muenchen	BOLD:AAY8844	308[2n]
Vespidae	Discoelius zonalis	BC ZSM HYM 13007	Germany	06-Jul-2011	SNSB, Zoologische Staatssammlung Muenchen	BOLD:AAY8844	658[0n]
Vespidae	Discoelius zonalis	BC ZSM HYM 08203	Germany	14-Aug-2010	SNSB, Zoologische Staatssammlung Muenchen	BOLD:AAY8844	634[0n]
Vespidae	Discoelius zonalis	BC ZSM HYM 08202	Germany	16-May-2004	SNSB, Zoologische Staatssammlung Muenchen	BOLD:AAY8844	658[0n]
Vespidae	Discoelius zonalis	BC ZSM HYM 08201	Germany	31-Aug-2008	SNSB, Zoologische Staatssammlung Muenchen	BOLD:AAY8844	658[0n]
Vespidae	Discoelius zonalis	BC ZSM HYM 08200	Germany	28-Jul-2009	SNSB, Zoologische Staatssammlung Muenchen	BOLD:AAY8844	658[0n]
Vespidae	Discoelius zonalis	BC ZSM HYM 17203	Germany	18-Jul-2012	SNSB, Zoologische Staatssammlung Muenchen	BOLD:AAY8844	658[0n]
Vespidae	Discoelius zonalis	BC ZSM HYM 13006	Germany	06-Jun-2011	SNSB, Zoologische Staatssammlung Muenchen	BOLD:AAY8844	658[0n]
Vespidae	Dolichovespula adulterina	BC ZSM HYM 05042	Germany	13-Jun-2007	SNSB, Zoologische Staatssammlung Muenchen	BOLD:AAI6347	658[0n]
Vespidae	Dolichovespula adulterina	BC ZSM HYM 05015	Germany	01-Aug-2007	SNSB, Zoologische Staatssammlung Muenchen	BOLD:AAI6347	658[0n]
Vespidae	Dolichovespula adulterina	BC ZSM HYM 05013	Germany	15-Jun-2008	SNSB, Zoologische Staatssammlung Muenchen	BOLD:AAI6347	626[0n]
Vespidae	Dolichovespula adulterina	BC ZSM HYM 05014	Germany	15-Jun-2008	SNSB, Zoologische Staatssammlung Muenchen	BOLD:AAI6347	658[0n]
Vespidae	Dolichovespula adulterina	BC ZSM HYM 05016	Germany	23-Jul-2006	SNSB, Zoologische Staatssammlung Muenchen	BOLD:AAI6347	658[0n]
Vespidae	Dolichovespula media	BC ZSM HYM 16990	Germany	18-Jul-2012	SNSB, Zoologische Staatssammlung Muenchen	BOLD:AAB6388	658[0n]
Vespidae	Dolichovespula media	BC ZSM HYM 05034	Germany	29-May-2008	SNSB, Zoologische Staatssammlung Muenchen	BOLD:AAB6388	658[0n]
Vespidae	Dolichovespula media	BC ZSM HYM 05033	Germany	01-May-2007	SNSB, Zoologische Staatssammlung Muenchen	BOLD:AAB6388	658[0n]
Vespidae	Dolichovespula norwegica	BC ZSM HYM 13973	Germany	23-Jul-2006	SNSB, Zoologische Staatssammlung Muenchen	BOLD:AAA6640	658[0n]

Family	Species	Specimen ID	Country	Collection Date	Depository	BIN	COI-5P
Vespidae	Dolichovespula norwegica	BC ZSM HYM 19932	Italy	05-Sep-2013	SNSB, Zoologische Staatssammlung Muenchen	BOLD:AAA6640	658[0n]
Vespidae	Dolichovespula norwegica	BC ZSM HYM 19931	Italy	05-Sep-2013	SNSB, Zoologische Staatssammlung Muenchen	BOLD:AAA6640	658[0n]
Vespidae	Dolichovespula norwegica	BC ZSM HYM 05038	Germany	28-Jul-2006	SNSB, Zoologische Staatssammlung Muenchen	BOLD:AAA6640	658[0n]
Vespidae	Dolichovespula norwegica	BC ZSM HYM 05037	Germany	01-May-2007	SNSB, Zoologische Staatssammlung Muenchen	BOLD:AAA6640	658[0n]
Vespidae	Dolichovespula norwegica	BC ZSM HYM 05036	Germany	23-Jul-2006	SNSB, Zoologische Staatssammlung Muenchen	BOLD:AAA6640	658[0n]
Vespidae	Dolichovespula norwegica	BC ZSM HYM 21082	Italy	04-Sep-2013	SNSB, Zoologische Staatssammlung Muenchen	BOLD:AAA6640	658[0n]
Vespidae	Dolichovespula norwegica	BC ZSM HYM 05035	Germany	28-Jul-2006	SNSB, Zoologische Staatssammlung Muenchen	BOLD:AAA6640	658[0n]
Vespidae	Dolichovespula omissa	BC ZSM HYM 05018	Germany	01-Aug-2007	SNSB, Zoologische Staatssammlung Muenchen	BOLD:AAN4303	658[0n]
Vespidae	Dolichovespula omissa	BC ZSM HYM 05017	Germany	01-May-2007	SNSB, Zoologische Staatssammlung Muenchen	BOLD:AAN4303	637[1n]
Vespidae	Dolichovespula omissa	BC ZSM HYM 17212	Germany	18-Jul-2012	SNSB, Zoologische Staatssammlung Muenchen	BOLD:AAN4303	658[0n]
Vespidae	Dolichovespula pacifica	BC ZSM HYM 11064	Sweden	05-Aug-2009	SNSB, Zoologische Staatssammlung Muenchen	BOLD:ACL8383	658[0n]
Vespidae	Dolichovespula pacifica	BC ZSM HYM 11063	Sweden	06-Jun-2009	SNSB, Zoologische Staatssammlung Muenchen	BOLD:ACL8383	658[0n]
Vespidae	Dolichovespula saxonica	BC ZSM HYM 13968	Germany	02-Apr-2012	SNSB, Zoologische Staatssammlung Muenchen	BOLD:AAM3988	658[0n]
Vespidae	Dolichovespula saxonica	BC ZSM HYM 13967	Germany	02-Apr-2012	SNSB, Zoologische Staatssammlung Muenchen	BOLD:AAM3988	658[0n]
Vespidae	Dolichovespula saxonica	BC ZSM HYM 13966	Germany	02-Apr-2012	SNSB, Zoologische Staatssammlung Muenchen	BOLD:AAM3988	658[0n]
Vespidae	Dolichovespula saxonica	BC ZSM HYM 19930	Germany	17-Jul-2011	SNSB, Zoologische Staatssammlung Muenchen	BOLD:AAM3988	658[0n]
Vespidae	Dolichovespula saxonica	BC ZSM HYM 16999	Germany	18-Jul-2012	SNSB, Zoologische Staatssammlung Muenchen	BOLD:AAM3988	658[0n]
Vespidae	Dolichovespula saxonica	BC ZSM HYM 05041	Germany	01-Jun-2007	SNSB, Zoologische Staatssammlung Muenchen	BOLD:AAM3988	658[0n]
Vespidae	Dolichovespula saxonica	BC ZSM HYM 05040	Germany	01-May-2007	SNSB, Zoologische Staatssammlung Muenchen	BOLD:AAM3988	658[0n]
Vespidae	Dolichovespula saxonica	BC ZSM HYM 05039	Germany	01-May-2007	SNSB, Zoologische Staatssammlung Muenchen	BOLD:AAM3988	635[0n]
Vespidae	Dolichovespula sylvestris	BC ZSM HYM 13970	Germany	09-Jul-2011	SNSB, Zoologische Staatssammlung Muenchen	BOLD:AAM3989	658[0n]
Vespidae	Dolichovespula sylvestris	BC ZSM HYM 13969	Germany	02-Apr-2012	SNSB, Zoologische Staatssammlung Muenchen	BOLD:AAM3989	658[0n]
Vespidae	Dolichovespula sylvestris	BC ZSM HYM 16998	Germany	18-Jul-2012	SNSB, Zoologische Staatssammlung Muenchen	BOLD:AAM3989	658[0n]
Vespidae	Dolichovespula sylvestris	BC ZSM HYM 05046	France	15-Jul-2009	SNSB, Zoologische Staatssammlung Muenchen	BOLD:AAM3991	658[0n]
Vespidae	Dolichovespula sylvestris	BC ZSM HYM 05044	Italy	17-Jul-2009	SNSB, Zoologische Staatssammlung Muenchen	BOLD:AAM3990	658[0n]
Vespidae	Dolichovespula sylvestris	BC ZSM HYM 05043	Germany	01-May-2007	SNSB, Zoologische Staatssammlung Muenchen	BOLD:AAM3989	658[0n]
Vespidae	Dolichovespula sylvestris	BC ZSM HYM 17211	Germany	04-Aug-2012	SNSB, Zoologische Staatssammlung Muenchen	BOLD:AAM3989	658[0n]
Vespidae	Dolichovespula sylvestris	BC ZSM HYM 15266	Germany	04-Aug-2012	SNSB, Zoologische Staatssammlung Muenchen	BOLD:AAM3989	658[0n]
Vespidae	Dolichovespula sylvestris	BC ZSM HYM 10195	Germany	09-Jul-2011	SNSB, Zoologische Staatssammlung Muenchen	BOLD:AAM3989	658[0n]
Vespidae	Dolichovespula sylvestris	BC ZSM HYM 10194	Germany	09-Jul-2011	SNSB, Zoologische Staatssammlung Muenchen	BOLD:AAM3989	658[0n]
Vespidae	Dolichovespula sylvestris	BC ZSM HYM 05045	France	14-Jul-2009	SNSB, Zoologische Staatssammlung Muenchen	BOLD:AAM3991	658[0n]
Vespidae	Eumenes coarctatus	BC ZSM HYM 04995	France	13-Jul-2007	SNSB, Zoologische Staatssammlung Muenchen	BOLD:ACE4606	614[0n]
Vespidae	Eumenes coarctatus	BC ZSM HYM 04994	France	12-Jul-2009	SNSB, Zoologische Staatssammlung Muenchen	BOLD:ACE4606	658[1n]
Vespidae	Eumenes coarctatus	BC ZSM HYM 04969	Germany	06-Sep-2008	SNSB, Zoologische Staatssammlung Muenchen	BOLD:AAN3538	658[0n]
Vespidae	Eumenes coarctatus	BC ZSM HYM 17363	Italy	20-Jul-2012	SNSB, Zoologische Staatssammlung Muenchen	BOLD:ACE4606	658[0n]
Vespidae	Eumenes coarctatus	BC ZSM HYM 17362	Italy	20-Jul-2012	SNSB, Zoologische Staatssammlung Muenchen	BOLD:ACE4606	658[0n]
Vespidae	Eumenes coarctatus	BC ZSM HYM 21143	Cyprus	20-Jun-2013	SNSB, Zoologische Staatssammlung Muenchen	BOLD:AAN4043	658[0n]
Vespidae	Eumenes coarctatus	BC ZSM HYM 21142	Cyprus	20-Jun-2013	SNSB, Zoologische Staatssammlung Muenchen	BOLD:AAN4043	658[0n]
Vespidae	Eumenes coarctatus	BC ZSM HYM 21141	Cyprus	20-Jun-2013	SNSB, Zoologische Staatssammlung Muenchen	BOLD:AAN4043	658[0n]
Vespidae	Eumenes coarctatus	BC ZSM HYM 21140	Cyprus	20-Jun-2013	SNSB, Zoologische Staatssammlung Muenchen	BOLD:AAN4043	658[0n]
Vespidae	Eumenes coarctatus	BC ZSM HYM 17365	Italy	20-Jul-2012	SNSB, Zoologische Staatssammlung Muenchen	BOLD:ACE4606	658[0n]
Vespidae	Eumenes coarctatus	BC ZSM HYM 04986	Italy	09-Jul-2006	SNSB, Zoologische Staatssammlung Muenchen	BOLD:ACE4606	658[1n]
Vespidae	Eumenes coarctatus	BC ZSM HYM 17372	Germany	18-Jul-2012	SNSB, Zoologische Staatssammlung Muenchen	BOLD:AAN3538	658[0n]
Vespidae	Eumenes coarctatus	BC ZSM HYM 15254	Germany	28-Aug-2012	SNSB, Zoologische Staatssammlung Muenchen	BOLD:AAN3538	658[0n]
Vespidae	Eumenes coarctatus	BC ZSM HYM 15253	Germany	28-Aug-2012	SNSB, Zoologische Staatssammlung Muenchen	BOLD:AAN3538	658[0n]
Vespidae	Eumenes coarctatus	BC ZSM HYM 15252	Germany	28-Aug-2012	SNSB, Zoologische Staatssammlung Muenchen	BOLD:AAN3538	658[0n]
Vespidae	Eumenes coarctatus	BC ZSM HYM 04971	Germany	19-Jul-2008	SNSB, Zoologische Staatssammlung Muenchen	BOLD:AAN3538	619[0n]

Family	Species	Specimen ID	Country	Collection Date	Depository	BIN	COI-5P
Vespidae	Eumenes coarctatus	BC ZSM HYM 04970	Germany	03-Jul-2008	SNSB, Zoologische Staatssammlung Muenchen	BOLD:AAN3538	658[0n]
Vespidae	Eumenes coarctatus	BC ZSM HYM 04968	Germany	19-Jul-2008	SNSB, Zoologische Staatssammlung Muenchen	BOLD:AAN3538	658[0n]
Vespidae	Eumenes coarctatus	BC ZSM HYM 09887	Germany	22-Jul-2009	SNSB, Zoologische Staatssammlung Muenchen	BOLD:AAN3538	658[0n]
Vespidae	Eumenes coarctatus	BC ZSM HYM 10213	Italy	17-Jul-2011	SNSB, Zoologische Staatssammlung Muenchen	BOLD:ACE4606	658[0n]
Vespidae	Eumenes coarctatus	BC-ZSM-HYM-29771-G07	Italy	27-Jul-2007	SNSB, Zoologische Staatssammlung Muenchen	BOLD:ACE4606	614[0n]
Vespidae	Eumenes coarctatus	BC-ZSM-HYM-29771-G06	Italy	27-Jul-2007	SNSB, Zoologische Staatssammlung Muenchen	BOLD:ACE4606	637[0n]
Vespidae	Eumenes coarctatus	BC ZSM HYM 07818	Germany	20-Jun-1993	SNSB, Zoologische Staatssammlung Muenchen	BOLD:AAN3538	638[1n]
Vespidae	Eumenes coronatus	BC ZSM HYM 17366	Germany	18-Jul-2012	SNSB, Zoologische Staatssammlung Muenchen	BOLD:AAN3639	658[0n]
Vespidae	Eumenes coronatus	BC ZSM HYM 13092	Germany	04-Aug-2010	SNSB, Zoologische Staatssammlung Muenchen	BOLD:AAN3639	658[0n]
Vespidae	Eumenes coronatus	BC ZSM HYM 17367	Germany	18-Jul-2012	SNSB, Zoologische Staatssammlung Muenchen	BOLD:AAN3639	658[0n]
Vespidae	Eumenes coronatus	BC ZSM HYM 08207	Germany	01-Sep-2010	SNSB, Zoologische Staatssammlung Muenchen	BOLD:AAN3639	658[0n]
Vespidae	Eumenes coronatus	BC ZSM HYM 08206	Germany	03-Oct-2010	SNSB, Zoologische Staatssammlung Muenchen	BOLD:AAN3639	658[0n]
Vespidae	Eumenes coronatus	BC ZSM HYM 08205	Germany	05-Oct-2010	SNSB, Zoologische Staatssammlung Muenchen	BOLD:AAN3639	658[0n]
Vespidae	Eumenes coronatus	BC ZSM HYM 08204	Germany	01-Sep-2010	SNSB, Zoologische Staatssammlung Muenchen	BOLD:AAN3639	658[0n]
Vespidae	Eumenes coronatus	BC ZSM HYM 15263	Germany	12-Aug-2012	SNSB, Zoologische Staatssammlung Muenchen	BOLD:AAN3639	658[0n]
Vespidae	Eumenes coronatus	BC ZSM HYM 04975	Germany	07-Jun-2007	SNSB, Zoologische Staatssammlung Muenchen	BOLD:AAN3639	658[0n]
Vespidae	Eumenes coronatus	BC ZSM HYM 04974	Germany	19-Jul-2008	SNSB, Zoologische Staatssammlung Muenchen	BOLD:AAN3639	658[0n]
Vespidae	Eumenes coronatus	BC ZSM HYM 04973	Germany	19-Jul-2008	SNSB, Zoologische Staatssammlung Muenchen	BOLD:AAN3639	658[0n]
Vespidae	Eumenes coronatus	BC ZSM HYM 04972	Germany	25-May-2008	SNSB, Zoologische Staatssammlung Muenchen	BOLD:AAN3639	658[0n]
Vespidae	Eumenes coronatus	BC ZSM HYM 13005	Germany	31-Jul-2008	SNSB, Zoologische Staatssammlung Muenchen	BOLD:AAN3639	566[0n]
Vespidae	Eumenes coronatus	BC ZSM HYM 13004	Germany	04-Aug-2010	SNSB, Zoologische Staatssammlung Muenchen	BOLD:AAN3639	658[0n]
Vespidae	Eumenes coronatus	BC ZSM HYM 13003	Germany	04-Aug-2010	SNSB, Zoologische Staatssammlung Muenchen	BOLD:AAN3639	658[0n]
Vespidae	Eumenes coronatus	BC ZSM HYM 13002	Germany	26-Aug-2008	SNSB, Zoologische Staatssammlung Muenchen	BOLD:AAN3639	658[0n]
Vespidae	Eumenes coronatus	BC ZSM HYM 07819	Germany	02-Jul-2000	SNSB, Zoologische Staatssammlung Muenchen	BOLD:AAN3639	611[0n]
Vespidae	Eumenes dubius	BC ZSM HYM 21145	Cyprus	20-Jun-2013	SNSB, Zoologische Staatssammlung Muenchen	BOLD:ACR5018	658[0n]
Vespidae	Eumenes dubius	BC ZSM HYM 21144	Cyprus	20-Jun-2013	SNSB, Zoologische Staatssammlung Muenchen	BOLD:ACR5018	658[0n]
Vespidae	Eumenes dubius	BC ZSM HYM 24031	Spain	28-Jul-2010	SNSB, Zoologische Staatssammlung Muenchen	BOLD:ACV9421	658[0n]
Vespidae	Eumenes dubius	BC ZSM HYM 24029	Spain	06-Aug-2007	SNSB, Zoologische Staatssammlung Muenchen	BOLD:ACV9421	658[0n]
Vespidae	Eumenes mediterraneus	BC ZSM HYM 21147	Cyprus	20-Jun-2013	SNSB, Zoologische Staatssammlung Muenchen	BOLD:ACR5439	658[0n]
Vespidae	Eumenes mediterraneus	BC ZSM HYM 21146	Cyprus	20-Jun-2013	SNSB, Zoologische Staatssammlung Muenchen	BOLD:ACR5439	658[0n]
Vespidae	Eumenes papillarius	BC ZSM HYM 13093	Germany	16-May-2000	SNSB, Zoologische Staatssammlung Muenchen	BOLD:AAY8811	658[0n]
Vespidae	Eumenes papillarius	BC ZSM HYM 08217	Germany	10-Sep-2010	SNSB, Zoologische Staatssammlung Muenchen	BOLD:AAY8811	658[0n]
Vespidae	Eumenes papillarius	BC ZSM HYM 08216	Germany	03-Oct-2010	SNSB, Zoologische Staatssammlung Muenchen	BOLD:AAY8811	658[0n]
Vespidae	Eumenes papillarius	BC ZSM HYM 04981	Italy	13-Jul-2000	SNSB, Zoologische Staatssammlung Muenchen	BOLD:AAY8811	614[0n]
Vespidae	Eumenes papillarius	BC ZSM HYM 13095	Germany	09-Jun-2000	SNSB, Zoologische Staatssammlung Muenchen	BOLD:AAY8811	658[0n]
Vespidae	Eumenes papillarius	BC ZSM HYM 13094	Germany	06-Jul-2001	SNSB, Zoologische Staatssammlung Muenchen	BOLD:AAY8811	658[0n]
Vespidae	Eumenes papillarius	BC ZSM HYM 09886	Germany	27-Jun-2011	SNSB, Zoologische Staatssammlung Muenchen	BOLD:AAY8811	658[0n]
Vespidae	Eumenes papillarius	BC ZSM HYM 09885	Germany	14-Jun-2011	SNSB, Zoologische Staatssammlung Muenchen	BOLD:AAY8811	658[0n]
Vespidae	Eumenes papillarius	BC ZSM HYM 09884	Germany	14-Jun-2011	SNSB, Zoologische Staatssammlung Muenchen	BOLD:AAY8811	658[0n]
Vespidae	Eumenes papillarius	BC ZSM HYM 08219	Germany	28-Jul-2009	SNSB, Zoologische Staatssammlung Muenchen	BOLD:AAY8811	658[0n]
Vespidae	Eumenes papillarius	BC ZSM HYM 08218	Germany	30-May-2009	SNSB, Zoologische Staatssammlung Muenchen	BOLD:AAY8811	658[0n]
Vespidae	Eumenes pedunculatus	BC ZSM HYM 13990	Germany	26-Jun-2010	SNSB, Zoologische Staatssammlung Muenchen	BOLD:AAL4355	658[0n]
Vespidae	Eumenes pedunculatus	BC ZSM HYM 00045	Germany	21-May-2007	SNSB, Zoologische Staatssammlung Muenchen	BOLD:AAL4355	658[0n]
Vespidae	Eumenes pedunculatus	BC ZSM HYM 17371	Germany	18-Jul-2012	SNSB, Zoologische Staatssammlung Muenchen	BOLD:AAL4355	658[0n]
Vespidae	Eumenes pedunculatus	BC ZSM HYM 17369	Germany	18-Jul-2012	SNSB, Zoologische Staatssammlung Muenchen	BOLD:AAL4355	658[0n]
Vespidae	Eumenes pedunculatus	BC ZSM HYM 17368	Germany	18-Jul-2012	SNSB, Zoologische Staatssammlung Muenchen	BOLD:AAL4355	658[0n]
Vespidae	Eumenes pedunculatus	BC ZSM HYM 17370	Germany	18-Jul-2012	SNSB, Zoologische Staatssammlung Muenchen	BOLD:AAL4355	614[1n]

Family	Species	Specimen ID	Country	Collection Date	Depository	BIN	COI-5P
Vespidae	Eumenes pedunculatus	BC ZSM HYM 15256	Germany	12-Aug-2012	SNSB, Zoologische Staatssammlung Muenchen	BOLD:AAL4355	658[0n]
Vespidae	Eumenes pedunculatus	BC ZSM HYM 15255	Germany	28-Aug-2012	SNSB, Zoologische Staatssammlung Muenchen	BOLD:AAL4355	658[0n]
Vespidae	Eumenes pedunculatus	BC ZSM HYM 04980	Germany	07-Jul-2008	SNSB, Zoologische Staatssammlung Muenchen	BOLD:AAL4355	658[0n]
Vespidae	Eumenes pedunculatus	BC ZSM HYM 04979	Germany	16-Aug-2008	SNSB, Zoologische Staatssammlung Muenchen	BOLD:AAL4355	658[0n]
Vespidae	Eumenes pedunculatus	BC ZSM HYM 04978	Germany	16-Jul-2009	SNSB, Zoologische Staatssammlung Muenchen	BOLD:AAL4355	658[0n]
Vespidae	Eumenes pedunculatus	BC ZSM HYM 04977	Germany	16-Aug-2008	SNSB, Zoologische Staatssammlung Muenchen	BOLD:AAL4355	614[0n]
Vespidae	Eumenes pedunculatus	BC ZSM HYM 04976	Germany	07-Jun-2007	SNSB, Zoologische Staatssammlung Muenchen	BOLD:AAL4355	658[0n]
Vespidae	Eumenes pedunculatus	BC ZSM HYM 09883	Germany	14-Jun-2011	SNSB, Zoologische Staatssammlung Muenchen	BOLD:AAL4355	658[0n]
Vespidae	Eumenes pedunculatus	BC ZSM HYM 10215	Germany	16-Jul-2011	SNSB, Zoologische Staatssammlung Muenchen	BOLD:AAL4355	658[0n]
Vespidae	Eumenes pedunculatus	BC ZSM HYM 08263	Germany	19-Apr-2011	SNSB, Zoologische Staatssammlung Muenchen	BOLD:AAL4355	658[0n]
Vespidae	Eumenes pomiformis	BC ZSM HYM 23636	Kyrgyzstan	14-Jul-2014	SNSB, Zoologische Staatssammlung Muenchen	BOLD:ACV9324	664[0n]
Vespidae	Eumenes pomiformis	BC ZSM HYM 17479	Italy	20-Jul-2012	SNSB, Zoologische Staatssammlung Muenchen	BOLD:AAN4044	658[0n]
Vespidae	Eumenes pomiformis	BC ZSM HYM 04985	Italy	27-Jul-2007	SNSB, Zoologische Staatssammlung Muenchen	BOLD:AAN4044	658[0n]
Vespidae	Eumenes pomiformis	BC ZSM HYM 04984	Italy	27-Jul-2007	SNSB, Zoologische Staatssammlung Muenchen	BOLD:AAN4044	658[0n]
Vespidae	Eumenes pomiformis	BC ZSM HYM 04983	Italy	25-Jul-1999	SNSB, Zoologische Staatssammlung Muenchen	BOLD:AAN4044	658[0n]
Vespidae	Eumenes pomiformis	BC ZSM HYM 04982	Italy	14-Jul-2000	SNSB, Zoologische Staatssammlung Muenchen	BOLD:AAN4044	625[1n]
Vespidae	Eumenes pomiformis	BC ZSM HYM 10214	Italy	17-Jul-2011	SNSB, Zoologische Staatssammlung Muenchen	BOLD:AAN4044	658[1n]
Vespidae	Eumenes pomiformis	BC ZSM HYM 10212	Italy	17-Jul-2011	SNSB, Zoologische Staatssammlung Muenchen	BOLD:AAN4044	658[0n]
Vespidae	Eumenes pomiformis	BC ZSM HYM 10210	Italy	17-Jul-2011	SNSB, Zoologische Staatssammlung Muenchen	BOLD:AAN4044	658[0n]
Vespidae	Eumenes sareptanus	BC ZSM HYM 04996	Germany	03-Jun-1997	SNSB, Zoologische Staatssammlung Muenchen	BOLD:ABA9380	421[0n]
Vespidae	Eumenes sareptanus	BC ZSM HYM 13128	Hungary	13-Aug-2011	SNSB, Zoologische Staatssammlung Muenchen	BOLD:ABA9380	658[0n]
Vespidae	Eumenes sareptanus	BC ZSM HYM 13127	Hungary	13-Aug-2011	SNSB, Zoologische Staatssammlung Muenchen	BOLD:ABA9380	658[0n]
Vespidae	Eumenes sareptanus	BC ZSM HYM 12909	Hungary	11-Aug-2011	SNSB, Zoologische Staatssammlung Muenchen	BOLD:ABA9380	658[0n]
Vespidae	Eumenes sareptanus	BC ZSM HYM 13057	Germany	05-Jun-1996	SNSB, Zoologische Staatssammlung Muenchen	BOLD:ABA9380	658[0n]
Vespidae	Eumenes sareptanus	BC ZSM HYM 24028	Spain	25-Jun-2011	SNSB, Zoologische Staatssammlung Muenchen	BOLD:ABA9380	658[0n]
Vespidae	Eumenes sareptanus	BC ZSM HYM 13056	Germany	24-Jul-1997	SNSB, Zoologische Staatssammlung Muenchen	BOLD:ABA9380	658[0n]
Vespidae	Eumenes sareptanus	BC ZSM HYM 13055	Germany	24-Jul-1997	SNSB, Zoologische Staatssammlung Muenchen	BOLD:ABA9380	658[0n]
Vespidae	Eumenes sareptanus	BC ZSM HYM 10211	Italy	17-Jul-2011	SNSB, Zoologische Staatssammlung Muenchen	BOLD:ABA9380	658[0n]
Vespidae	Eumenes subpomiformis	BC-ZSM-HYM-29771-G05	France	12-Jul-2009	SNSB, Zoologische Staatssammlung Muenchen	BOLD:ACG1686	658[0n]
Vespidae	Euodynerus dantici	BC ZSM HYM 04789	France	07-Jun-1997	SNSB, Zoologische Staatssammlung Muenchen	BOLD:AAM5315	658[0n]
Vespidae	Euodynerus dantici	BC ZSM HYM 04788	France	16-Jul-2009	SNSB, Zoologische Staatssammlung Muenchen	BOLD:AAM5315	658[0n]
Vespidae	Euodynerus disconotatus	GBOL18843	Cyprus	20-Jun-2013	SNSB, Zoologische Staatssammlung Muenchen	BOLD:ADD0366	658[0n]
Vespidae	Euodynerus disconotatus	GBOL18842	Cyprus	20-Jun-2013	SNSB, Zoologische Staatssammlung Muenchen	BOLD:ADD0366	589[0n]
Vespidae	Euodynerus disconotatus	GBOL18841	Cyprus	20-Jun-2013	SNSB, Zoologische Staatssammlung Muenchen	BOLD:ADD0366	658[0n]
Vespidae	Euodynerus notatus	BC ZSM HYM 14400	Germany	30-Jun-2012	SNSB, Zoologische Staatssammlung Muenchen	BOLD:AAN0689	658[0n]
Vespidae	Euodynerus notatus	BC ZSM HYM 14399	Germany	30-Jun-2012	SNSB, Zoologische Staatssammlung Muenchen	BOLD:AAN0689	658[0n]
Vespidae	Euodynerus notatus	BC ZSM HYM 08211	Germany	01-Jun-1999	SNSB, Zoologische Staatssammlung Muenchen	BOLD:AAN0689	658[0n]
Vespidae	Euodynerus notatus	BC ZSM HYM 08210	Germany	25-May-2005	SNSB, Zoologische Staatssammlung Muenchen	BOLD:AAN0689	658[0n]
Vespidae	Euodynerus notatus	BC ZSM HYM 08209	Germany	10-Jul-2008	SNSB, Zoologische Staatssammlung Muenchen	BOLD:AAN0689	658[0n]
Vespidae	Euodynerus notatus	BC ZSM HYM 08208	Germany	09-Jun-2009	SNSB, Zoologische Staatssammlung Muenchen	BOLD:AAN0689	658[0n]
Vespidae	Euodynerus notatus	BC ZSM HYM 14398	Germany	30-Jun-2012	SNSB, Zoologische Staatssammlung Muenchen	BOLD:AAN0689	658[0n]
Vespidae	Euodynerus notatus	BC ZSM HYM 14397	Germany	30-Jun-2012	SNSB, Zoologische Staatssammlung Muenchen	BOLD:AAN0689	658[0n]
Vespidae	Euodynerus notatus	BC ZSM HYM 04791	Germany	01-Jul-2008	SNSB, Zoologische Staatssammlung Muenchen	BOLD:AAN0689	658[0n]
Vespidae	Euodynerus notatus	BC ZSM HYM 12991	Germany	02-Jul-2008	SNSB, Zoologische Staatssammlung Muenchen	BOLD:AAN0689	658[0n]
Vespidae	Euodynerus notatus	BC ZSM HYM 12990	Germany	24-Jun-2001	SNSB, Zoologische Staatssammlung Muenchen	BOLD:AAN0689	658[0n]
Vespidae	Euodynerus notatus	BC ZSM HYM 12988	Germany	10-Jun-2010	SNSB, Zoologische Staatssammlung Muenchen	BOLD:AAN0689	658[0n]
Vespidae	Euodynerus quadrifasciatus	BC ZSM HYM 17202	Germany	18-Jul-2012	SNSB, Zoologische Staatssammlung Muenchen	BOLD:ABY9039	658[0n]

Family	Species	Specimen ID	Country	Collection Date	Depository	BIN	COI-5P
Vespidae	Euodynerus quadrifasciatus	BC ZSM HYM 17201	Germany	18-Jul-2012	SNSB, Zoologische Staatssammlung Muenchen	BOLD:ABY9039	658[0n]
Vespidae	Euodynerus quadrifasciatus	BC ZSM HYM 04793	Italy	09-Jul-2006	SNSB, Zoologische Staatssammlung Muenchen	BOLD:ABY9039	658[0n]
Vespidae	Euodynerus quadrifasciatus	BC ZSM HYM 04792	Italy	13-May-2006	SNSB, Zoologische Staatssammlung Muenchen	BOLD:ABY9039	658[0n]
Vespidae	Euodynerus quadrifasciatus	BC ZSM HYM 06606	France	09-Jul-2010	SNSB, Zoologische Staatssammlung Muenchen	BOLD:ABY9039	658[0n]
Vespidae	Euodynerus quadrifasciatus	BC ZSM HYM 06594	France	13-Jul-2010	SNSB, Zoologische Staatssammlung Muenchen	BOLD:ABY9039	658[0n]
Vespidae	Gymnomerus laevipes	BC ZSM HYM 13981	Germany	25-Jun-2006	SNSB, Zoologische Staatssammlung Muenchen	BOLD:AAL3407	658[0n]
Vespidae	Gymnomerus laevipes	BC ZSM HYM 19925	Germany	07-Jul-2013	SNSB, Zoologische Staatssammlung Muenchen	BOLD:AAL3407	658[0n]
Vespidae	Gymnomerus laevipes	BC ZSM HYM 04797	Germany	07-Jun-2007	SNSB, Zoologische Staatssammlung Muenchen	BOLD:AAL3407	658[0n]
Vespidae	Gymnomerus laevipes	BC ZSM HYM 04795	Germany	05-Aug-2007	SNSB, Zoologische Staatssammlung Muenchen	BOLD:AAL3407	658[0n]
Vespidae	Gymnomerus laevipes	BC ZSM HYM 21078	Germany	06-Jul-2013	SNSB, Zoologische Staatssammlung Muenchen	BOLD:AAL3407	658[0n]
Vespidae	Gymnomerus laevipes	BC ZSM HYM 08215	Germany	28-May-2006	SNSB, Zoologische Staatssammlung Muenchen	BOLD:AAL3407	658[0n]
Vespidae	Gymnomerus laevipes	BC ZSM HYM 08214	Germany	13-Jun-2007	SNSB, Zoologische Staatssammlung Muenchen	BOLD:AAL3407	658[0n]
Vespidae	Gymnomerus laevipes	BC ZSM HYM 08213	Germany	20-Jun-2008	SNSB, Zoologische Staatssammlung Muenchen	BOLD:AAL3407	658[0n]
Vespidae	Gymnomerus laevipes	BC ZSM HYM 08212	Germany	05-Jun-2009	SNSB, Zoologische Staatssammlung Muenchen	BOLD:AAL3407	658[0n]
Vespidae	Gymnomerus laevipes	BC ZSM HYM 13062	Germany	02-Jun-2009	SNSB, Zoologische Staatssammlung Muenchen	BOLD:AAL3407	658[0n]
Vespidae	Gymnomerus laevipes	BC ZSM HYM 13061	Germany	29-Jun-2009	SNSB, Zoologische Staatssammlung Muenchen	BOLD:AAL3407	658[0n]
Vespidae	Gymnomerus laevipes	BC ZSM HYM 00047	Germany	21-May-2007	SNSB, Zoologische Staatssammlung Muenchen	BOLD:AAL3407	658[0n]
Vespidae	Gymnomerus laevipes	BC ZSM HYM 04796	Germany	01-Jun-2008	SNSB, Zoologische Staatssammlung Muenchen	BOLD:AAL3407	658[0n]
Vespidae	Gymnomerus laevipes	BC ZSM HYM 04794	Germany	21-May-2007	SNSB, Zoologische Staatssammlung Muenchen	BOLD:AAL3407	658[0n]
Vespidae	Katamenes arbustorum	BC ZSM HYM 04805	Italy	27-Jul-2007	SNSB, Zoologische Staatssammlung Muenchen	BOLD:AAM6807	658[0n]
Vespidae	Katamenes arbustorum	BC ZSM HYM 04804	Italy	09-Jul-2006	SNSB, Zoologische Staatssammlung Muenchen	BOLD:AAM6807	658[0n]
Vespidae	Katamenes arbustorum	BC ZSM HYM 06630	France	15-Jul-2010	SNSB, Zoologische Staatssammlung Muenchen	BOLD:AAM6807	658[0n]
Vespidae	Katamenes arbustorum	BC ZSM HYM 06618	France	15-Jul-2010	SNSB, Zoologische Staatssammlung Muenchen	BOLD:AAM6807	658[0n]
Vespidae	Leptochilus alpestris	BC ZSM HYM 04808	Italy	17-Jun-2009	SNSB, Zoologische Staatssammlung Muenchen	BOLD:AAM9217	632[0n]
Vespidae	Leptochilus alpestris	BC ZSM HYM 04826	France	12-Jul-2009	SNSB, Zoologische Staatssammlung Muenchen	BOLD:AAM9218	658[0n]
Vespidae	Leptochilus alpestris	BC ZSM HYM 07397	France	12-Jul-2010	SNSB, Zoologische Staatssammlung Muenchen	BOLD:AAM9218	658[0n]
Vespidae	Leptochilus alpestris	BC ZSM HYM 07378	France	10-Jul-2010	SNSB, Zoologische Staatssammlung Muenchen	BOLD:AAM9217	658[0n]
Vespidae	Leptochilus moustirsensis	BC ZSM HYM 16244	France	10-Jul-2010	SNSB, Zoologische Staatssammlung Muenchen	BOLD:ACG1074	658[0n]
Vespidae	Leptochilus regulus	BC ZSM HYM 04809	France	12-Jul-2009	SNSB, Zoologische Staatssammlung Muenchen	BOLD:AAM9219	658[0n]
Vespidae	Leptochilus regulus	BC ZSM HYM 04810	France	13-Jul-2007	SNSB, Zoologische Staatssammlung Muenchen	BOLD:AAM9219	658[0n]
Vespidae	Leptochilus tarsatus	BC ZSM HYM 04813	Italy	11-Jul-2009	SNSB, Zoologische Staatssammlung Muenchen	BOLD:AAM4311	658[0n]
Vespidae	Leptochilus tarsatus	BC ZSM HYM 04812	Italy	11-Jul-2009	SNSB, Zoologische Staatssammlung Muenchen	BOLD:AAM4311	658[0n]
Vespidae	Leptochilus tarsatus	BC ZSM HYM 04811	Italy	11-Jul-2009	SNSB, Zoologische Staatssammlung Muenchen	BOLD:AAM4311	658[0n]
Vespidae	Microdynerus exilis	BC ZSM HYM 08227	Germany	14-Jun-2010	SNSB, Zoologische Staatssammlung Muenchen		421[0n]
Vespidae	Microdynerus exilis	BC ZSM HYM 08226	Germany	12-Jun-2009	SNSB, Zoologische Staatssammlung Muenchen		421[0n]
Vespidae	Microdynerus exilis	BC ZSM HYM 08225	Germany	29-May-2009	SNSB, Zoologische Staatssammlung Muenchen		421[0n]
Vespidae	Microdynerus exilis	BC ZSM HYM 08224	Germany	08-Jun-2008	SNSB, Zoologische Staatssammlung Muenchen		421[0n]
Vespidae	Microdynerus longicollis	BC ZSM HYM 04821	Germany	01-Jul-2008	SNSB, Zoologische Staatssammlung Muenchen	BOLD:AAN2278	658[0n]
Vespidae	Microdynerus nugdunensis	BC ZSM HYM 04818	Germany	30-May-1997	SNSB, Zoologische Staatssammlung Muenchen		306[2n]
Vespidae	Microdynerus parvulus	BC ZSM HYM 04825	Germany	07-Jun-2007	SNSB, Zoologische Staatssammlung Muenchen	BOLD:AAM3885	658[0n]
Vespidae	Microdynerus parvulus	BC ZSM HYM 04824	Germany	19-Jul-2008	SNSB, Zoologische Staatssammlung Muenchen	BOLD:AAM3885	658[0n]
Vespidae	Microdynerus parvulus	BC ZSM HYM 04823	Germany	07-Jun-2007	SNSB, Zoologische Staatssammlung Muenchen	BOLD:AAM3885	658[0n]
Vespidae	Microdynerus parvulus	BC ZSM HYM 04822	Germany	15-Jul-2008	SNSB, Zoologische Staatssammlung Muenchen	BOLD:AAM3885	624[0n]
Vespidae	Microdynerus parvulus	BC ZSM HYM 04817	Italy	03-Aug-1997	SNSB, Zoologische Staatssammlung Muenchen	BOLD:AAM3885	658[0n]
Vespidae	Microdynerus timidus	BC ZSM HYM 13285	Germany	20-Jun-2000	SNSB, Zoologische Staatssammlung Muenchen	BOLD:AAY8818	658[0n]
Vespidae	Microdynerus timidus	BC ZSM HYM 13284	Germany	20-Jun-2000	SNSB, Zoologische Staatssammlung Muenchen	BOLD:AAY8818	658[0n]
Vespidae	Microdynerus timidus	BC ZSM HYM 08231	Germany	10-Jun-2008	SNSB, Zoologische Staatssammlung Muenchen	BOLD:AAY8818	658[0n]

Family	Species	Specimen ID	Country	Collection Date	Depository	BIN	COI-5P
Vespidae	Microdynerus timidus	BC ZSM HYM 08230	Germany	26-Jun-2008	SNSB, Zoologische Staatssammlung Muenchen	BOLD:AAY8818	658[0n]
Vespidae	Microdynerus timidus	BC ZSM HYM 08229	Germany	03-Jul-2010	SNSB, Zoologische Staatssammlung Muenchen	BOLD:AAY8818	658[0n]
Vespidae	Microdynerus timidus	BC ZSM HYM 08228	Germany	26-Jul-2009	SNSB, Zoologische Staatssammlung Muenchen	BOLD:AAY8818	658[0n]
Vespidae	Odynerus alpinus	BC ZSM HYM 04798	France	14-Jul-2009	SNSB, Zoologische Staatssammlung Muenchen	BOLD:AAN1663	658[0n]
Vespidae	Odynerus alpinus	BC-ZSM-HYM-27676-D07	Germany	04-Jul-2014	SNSB, Zoologische Staatssammlung Muenchen	BOLD:AAN1663	637[0n]
Vespidae	Odynerus alpinus	BC ZSM HYM 06642	France	14-Jul-2010	SNSB, Zoologische Staatssammlung Muenchen	BOLD:AAN1663	658[0n]
Vespidae	Odynerus alpinus	BC ZSM HYM 06559	France	14-Jul-2010	SNSB, Zoologische Staatssammlung Muenchen	BOLD:AAN1663	658[0n]
Vespidae	Odynerus dusmeticus	GBOL19143	Spain	29-May-2004	SNSB, Zoologische Staatssammlung Muenchen	BOLD:ADE9903	614[0n]
Vespidae	Odynerus dusmeticus	GBOL19142	Spain	09-May-2009	SNSB, Zoologische Staatssammlung Muenchen	BOLD:ADE9903	658[0n]
Vespidae	Odynerus melanocephalus	BC ZSM HYM 19924	Germany	19-May-2013	SNSB, Zoologische Staatssammlung Muenchen	BOLD:AAW9811	658[0n]
Vespidae	Odynerus melanocephalus	BC ZSM HYM 21079	Germany	18-May-2013	SNSB, Zoologische Staatssammlung Muenchen	BOLD:AAW9811	658[0n]
Vespidae	Odynerus melanocephalus	BC ZSM HYM 12982	Germany	20-May-2009	SNSB, Zoologische Staatssammlung Muenchen	BOLD:AAW9811	658[0n]
Vespidae	Odynerus melanocephalus	BC ZSM HYM 12981	Germany	15-Jun-2009	SNSB, Zoologische Staatssammlung Muenchen	BOLD:AAW9811	658[0n]
Vespidae	Odynerus melanocephalus	BC ZSM HYM 12984	Germany	28-May-2008	SNSB, Zoologische Staatssammlung Muenchen	BOLD:AAW9811	658[0n]
Vespidae	Odynerus melanocephalus	BC ZSM HYM 12983	Germany	28-Jun-2004	SNSB, Zoologische Staatssammlung Muenchen	BOLD:AAW9811	658[0n]
Vespidae	Odynerus melanocephalus	BC ZSM HYM 06571	France	15-Jul-2010	SNSB, Zoologische Staatssammlung Muenchen	BOLD:AAW9811	658[0n]
Vespidae	Odynerus melanocephalus	BC ZSM HYM 08223	Germany	16-Jun-2010	SNSB, Zoologische Staatssammlung Muenchen	BOLD:AAW9811	658[0n]
Vespidae	Odynerus melanocephalus	BC ZSM HYM 08222	Germany	11-Jun-2010	SNSB, Zoologische Staatssammlung Muenchen	BOLD:AAW9811	658[0n]
Vespidae	Odynerus melanocephalus	BC ZSM HYM 08221	Germany	10-Jun-2007	SNSB, Zoologische Staatssammlung Muenchen	BOLD:AAW9811	658[0n]
Vespidae	Odynerus melanocephalus	BC ZSM HYM 08220	Germany	30-May-2008	SNSB, Zoologische Staatssammlung Muenchen	BOLD:AAW9811	658[0n]
Vespidae	Odynerus reniformis	BC ZSM HYM 12986	Germany	25-May-2009	SNSB, Zoologische Staatssammlung Muenchen	BOLD:AAV3568	658[0n]
Vespidae	Odynerus reniformis	BC ZSM HYM 12985	Germany	25-May-2009	SNSB, Zoologische Staatssammlung Muenchen	BOLD:AAV3568	658[0n]
Vespidae	Odynerus reniformis	BC ZSM HYM 06595	France	15-Jul-2010	SNSB, Zoologische Staatssammlung Muenchen	BOLD:AAV3568	658[0n]
Vespidae	Odynerus reniformis	BC ZSM HYM 06583	France	15-Jul-2010	SNSB, Zoologische Staatssammlung Muenchen	BOLD:AAV3568	658[0n]
Vespidae	Odynerus spinipes	BC ZSM HYM 13980	Germany	26-May-2005	SNSB, Zoologische Staatssammlung Muenchen	BOLD:AAK2980	658[0n]
Vespidae	Odynerus spinipes	BC ZSM HYM 00048	Germany	30-Apr-2007	SNSB, Zoologische Staatssammlung Muenchen	BOLD:AAK2980	658[0n]
Vespidae	Odynerus spinipes	BC ZSM HYM 13060	Germany	02-Jun-2003	SNSB, Zoologische Staatssammlung Muenchen	BOLD:AAK2980	658[0n]
Vespidae	Odynerus spinipes	BC ZSM HYM 13059	Germany	02-Jun-2003	SNSB, Zoologische Staatssammlung Muenchen	BOLD:AAK2980	658[0n]
Vespidae	Odynerus spinipes	BC ZSM HYM 04800	Germany	10-Jun-2008	SNSB, Zoologische Staatssammlung Muenchen	BOLD:AAK2980	658[0n]
Vespidae	Odynerus spinipes	BC ZSM HYM 04799	Poland	12-May-2008	SNSB, Zoologische Staatssammlung Muenchen	BOLD:AAK2980	658[0n]
Vespidae	Odynerus spinipes	BC ZSM HYM 14701	Germany	21-May-2012	SNSB, Zoologische Staatssammlung Muenchen	BOLD:AAK2980	658[0n]
Vespidae	Polistes albellus	BC ZSM HYM 05009	Germany	12-Aug-2003	SNSB, Zoologische Staatssammlung Muenchen	BOLD:AAN3553	658[0n]
Vespidae	Polistes albellus	BC ZSM HYM 05011	Germany	06-Sep-2006	SNSB, Zoologische Staatssammlung Muenchen	BOLD:AAN3553	658[0n]
Vespidae	Polistes albellus	BC ZSM HYM 15513	Switzerland	08-Sep-2012	SNSB, Zoologische Staatssammlung Muenchen	BOLD:AAN3553	658[0n]
Vespidae	Polistes atrimandibularis	BC ZSM HYM 05020	Italy	23-Aug-1995	SNSB, Zoologische Staatssammlung Muenchen	BOLD:AAN4297	658[0n]
Vespidae	Polistes atrimandibularis	BC ZSM HYM 05019	Italy	23-Aug-1995	SNSB, Zoologische Staatssammlung Muenchen	BOLD:AAN4297	658[0n]
Vespidae	Polistes austroccidentalis	BC ZSM HYM 22042	Morocco	12-Jun-2014	SNSB, Zoologische Staatssammlung Muenchen	BOLD:ACG1677	658[0n]
Vespidae	Polistes austroccidentalis	BC ZSM HYM 22339	Switzerland	07-Aug-1998	SNSB, Zoologische Staatssammlung Muenchen	BOLD:ACG1677	658[0n]
Vespidae	Polistes austroccidentalis	BC ZSM HYM 15527	France	12-Jul-2009	SNSB, Zoologische Staatssammlung Muenchen	BOLD:ACG1677	654[0n]
Vespidae	Polistes biglumis	BC ZSM HYM 19402	Italy	20-Jul-2012	SNSB, Zoologische Staatssammlung Muenchen	BOLD:AAN3552	658[0n]
Vespidae	Polistes biglumis	BC ZSM HYM 05005	Germany	19-Aug-2006	SNSB, Zoologische Staatssammlung Muenchen	BOLD:AAN3552	658[0n]
Vespidae	Polistes biglumis	BC ZSM HYM 15561	Germany	22-Jul-2004	SNSB, Zoologische Staatssammlung Muenchen	BOLD:AAN3552	658[0n]
Vespidae	Polistes bischoffi	BC ZSM HYM 15554	Croatia	24-Aug-2005	SNSB, Zoologische Staatssammlung Muenchen	BOLD:ACG2292	658[1n]
Vespidae	Polistes bischoffi	BC ZSM HYM 22343	Switzerland	26-Jun-2014	SNSB, Zoologische Staatssammlung Muenchen	BOLD:ACG2292	658[0n]
Vespidae	Polistes bischoffi	BC ZSM HYM 22341	Switzerland	19-Aug-2013	SNSB, Zoologische Staatssammlung Muenchen	BOLD:ACG2292	658[0n]
Vespidae	Polistes dominula	BC ZSM HYM 19394	Germany	18-Jul-2012	SNSB, Zoologische Staatssammlung Muenchen	BOLD:AAB7105	658[0n]
Vespidae	Polistes dominula	BC ZSM HYM 19392	Germany	18-Jul-2012	SNSB, Zoologische Staatssammlung Muenchen	BOLD:AAB7105	658[0n]

Family	Species	Specimen ID	Country	Collection Date	Depository	BIN	COI-5P
Vespidae	Polistes dominula	BC ZSM HYM 05003	France	16-Jul-2009	SNSB, Zoologische Staatssammlung Muenchen	BOLD:AAA9495	658[0n]
Vespidae	Polistes dominula	BC ZSM HYM 00044	Germany	16-Aug-2008	SNSB, Zoologische Staatssammlung Muenchen	BOLD:AAA9495	658[0n]
Vespidae	Polistes dominula	BC ZSM HYM 15565	Germany	18-Aug-2008	SNSB, Zoologische Staatssammlung Muenchen	BOLD:AAA9495	658[0n]
Vespidae	Polistes dominula	BC ZSM HYM 10201	Italy	17-Jul-2011	SNSB, Zoologische Staatssammlung Muenchen	BOLD:AAB7105	658[0n]
Vespidae	Polistes gallicus	BC ZSM HYM 13472	Croatia	28-Jul-2012	SNSB, Zoologische Staatssammlung Muenchen	BOLD:AAN3302	658[0n]
Vespidae	Polistes gallicus	BC ZSM HYM 05024	Italy	13-May-2006	SNSB, Zoologische Staatssammlung Muenchen	BOLD:AAN3302	658[0n]
Vespidae	Polistes gallicus	BC ZSM HYM 17494	Spain	20-Jun-2011	SNSB, Zoologische Staatssammlung Muenchen	BOLD:AAN3302	658[0n]
Vespidae	Polistes nimpha	BC ZSM HYM 14018	Germany	27-Jun-2011	SNSB, Zoologische Staatssammlung Muenchen	BOLD:ACC1661	658[0n]
Vespidae	Polistes nimpha	BC ZSM HYM 14017	Germany	09-Jul-2011	SNSB, Zoologische Staatssammlung Muenchen	BOLD:ACC1661	658[1n]
Vespidae	Polistes nimpha	BC ZSM HYM 14013	Germany	09-Jul-2011	SNSB, Zoologische Staatssammlung Muenchen	BOLD:ACC1661	602[0n]
Vespidae	Polistes nimpha	BC ZSM HYM 14012	Germany	16-Jul-2011	SNSB, Zoologische Staatssammlung Muenchen	BOLD:AAL0103	658[0n]
Vespidae	Polistes nimpha	BC ZSM HYM 19398	Germany	18-Jul-2012	SNSB, Zoologische Staatssammlung Muenchen	BOLD:AAL0103	658[0n]
Vespidae	Polistes nimpha	BC ZSM HYM 19397	Germany	18-Jul-2012	SNSB, Zoologische Staatssammlung Muenchen	BOLD:AAL0103	658[0n]
Vespidae	Polistes semenowi	BC ZSM HYM 22337	Switzerland	17-Aug-2013	SNSB, Zoologische Staatssammlung Muenchen	BOLD:ACG1290	658[0n]
Vespidae	Polistes semenowi	BC ZSM HYM 15529	Italy	27-Jul-2007	SNSB, Zoologische Staatssammlung Muenchen	BOLD:ACG1290	658[0n]
Vespidae	Polistes semenowi	BC ZSM HYM 15530	Italy	27-Jul-2007	SNSB, Zoologische Staatssammlung Muenchen	BOLD:ACG1290	658[0n]
Vespidae	Pterocheilus phaleratus	BC ZSM HYM 19923	Germany	09-Jun-2013	SNSB, Zoologische Staatssammlung Muenchen	BOLD:AAL2208	658[0n]
Vespidae	Pterocheilus phaleratus	BC ZSM HYM 14090	Germany	27-Jun-2011	SNSB, Zoologische Staatssammlung Muenchen	BOLD:AAL2208	658[0n]
Vespidae	Pterocheilus phaleratus	BC ZSM HYM 14089	Germany	27-Jun-2011	SNSB, Zoologische Staatssammlung Muenchen	BOLD:AAL2208	658[0n]
Vespidae	Pterocheilus phaleratus	BC ZSM HYM 14088	Germany	27-Jun-2011	SNSB, Zoologische Staatssammlung Muenchen	BOLD:AAL2208	658[0n]
Vespidae	Pterocheilus phaleratus	BC ZSM HYM 21077	Germany	08-Jun-2013	SNSB, Zoologische Staatssammlung Muenchen	BOLD:AAL2208	658[0n]
Vespidae	Pterocheilus phaleratus	BC ZSM HYM 04830	Germany	03-Jul-2008	SNSB, Zoologische Staatssammlung Muenchen	BOLD:AAL2208	658[0n]
Vespidae	Pterocheilus phaleratus	BC ZSM HYM 04829	Germany	03-Jul-2008	SNSB, Zoologische Staatssammlung Muenchen	BOLD:AAL2208	658[0n]
Vespidae	Pterocheilus phaleratus	BC ZSM HYM 04828	Germany	03-Jul-2008	SNSB, Zoologische Staatssammlung Muenchen	BOLD:AAL2208	658[0n]
Vespidae	Pterocheilus phaleratus	BC ZSM HYM 04827	Germany	03-Jul-2008	SNSB, Zoologische Staatssammlung Muenchen	BOLD:AAL2208	658[0n]
Vespidae	Pterocheilus phaleratus	BC ZSM HYM 00046	Germany	03-Jul-2008	SNSB, Zoologische Staatssammlung Muenchen	BOLD:AAL2208	658[0n]
Vespidae	Pterocheilus phaleratus	BC ZSM HYM 14699	Germany	21-May-2012	SNSB, Zoologische Staatssammlung Muenchen	BOLD:AAL2208	658[0n]
Vespidae	Pterocheilus phaleratus	BC ZSM HYM 14698	Germany	21-May-2012	SNSB, Zoologische Staatssammlung Muenchen	BOLD:AAL2208	658[0n]
Vespidae	Pterocheilus phaleratus	BC ZSM HYM 14697	Germany	02-May-2012	SNSB, Zoologische Staatssammlung Muenchen	BOLD:AAL2208	658[0n]
Vespidae	Stenodynerus bluethgeni	BC ZSM HYM 04841	Italy	09-Jul-2006	SNSB, Zoologische Staatssammlung Muenchen	BOLD:AAM4330	658[0n]
Vespidae	Stenodynerus bluethgeni	BC ZSM HYM 13299	Germany	07-Jun-1998	SNSB, Zoologische Staatssammlung Muenchen	BOLD:AAM4330	645[0n]
Vespidae	Stenodynerus bluethgeni	BC ZSM HYM 17383	Germany	18-Jul-2012	SNSB, Zoologische Staatssammlung Muenchen	BOLD:AAM4330	658[0n]
Vespidae	Stenodynerus bluethgeni	BC ZSM HYM 17385	Germany	18-Jul-2012	SNSB, Zoologische Staatssammlung Muenchen	BOLD:AAM4330	658[0n]
Vespidae	Stenodynerus bluethgeni	BC ZSM HYM 17196	Germany	18-Jul-2012	SNSB, Zoologische Staatssammlung Muenchen	BOLD:AAM4330	658[0n]
Vespidae	Stenodynerus bluethgeni	BC ZSM HYM 04834	Italy	27-Jul-2007	SNSB, Zoologische Staatssammlung Muenchen	BOLD:AAM4330	658[0n]
Vespidae	Stenodynerus bluethgeni	BC ZSM HYM 04832	Italy	12-Aug-2008	SNSB, Zoologische Staatssammlung Muenchen	BOLD:AAM4330	658[0n]
Vespidae	Stenodynerus bluethgeni	BC ZSM HYM 04831	France	17-Jul-2009	SNSB, Zoologische Staatssammlung Muenchen	BOLD:AAM4330	623[1n]
Vespidae	Stenodynerus bluethgeni	BC ZSM HYM 10220	Germany	16-Jul-2011	SNSB, Zoologische Staatssammlung Muenchen	BOLD:AAM4330	658[0n]
Vespidae	Stenodynerus bluethgeni	BC ZSM HYM 10218	Germany	09-Jul-2011	SNSB, Zoologische Staatssammlung Muenchen	BOLD:AAM4330	658[0n]
Vespidae	Stenodynerus bluethgeni	BC ZSM HYM 17384	Germany	18-Jul-2012	SNSB, Zoologische Staatssammlung Muenchen	BOLD:AAM4330	658[0n]
Vespidae	Stenodynerus bluethgeni	BC ZSM HYM 10219	Germany	16-Jul-2011	SNSB, Zoologische Staatssammlung Muenchen	BOLD:AAM4330	658[0n]
Vespidae	Stenodynerus bluethgeni	BC ZSM HYM 08264	Germany	19-Apr-2011	SNSB, Zoologische Staatssammlung Muenchen	BOLD:AAM4330	658[0n]
Vespidae	Stenodynerus chevrieranus	BC ZSM HYM 04835	Switzerland	29-Aug-1999	SNSB, Zoologische Staatssammlung Muenchen	BOLD:AAN0040	645[0n]
Vespidae	Stenodynerus chevrieranus	BC ZSM HYM 24663	Germany	06-May-2015	SNSB, Zoologische Staatssammlung Muenchen	BOLD:AAN0040	658[0n]
Vespidae	Stenodynerus chevrieranus	BC-ZSM-HYM-23654-G01	Slovakia	05-Aug-2008	SNSB, Zoologische Staatssammlung Muenchen	BOLD:AAN0040	658[0n]
Vespidae	Stenodynerus clypeopictus	BC-ZSM-HYM-23654-C07	Slovakia	06-Aug-2008	SNSB, Zoologische Staatssammlung Muenchen	BOLD:ADH6201	658[0n]
Vespidae	Stenodynerus jurinei	BC ZSM HYM 04836	Italy	09-Jul-2006	SNSB, Zoologische Staatssammlung Muenchen	BOLD:AAM3906	658[0n]

Family	Species	Specimen ID	Country	Collection Date	Depository	BIN	COI-5P
Vespidae	Stenodynerus jurinei	BC-ZSM-HYM-27676-D05	Germany	30-Jun-2013	SNSB, Zoologische Staatssammlung Muenchen	BOLD:AAM3906	658[0n]
Vespidae	Stenodynerus jurinei	BC ZSM HYM 04839	Italy	27-Jul-2007	SNSB, Zoologische Staatssammlung Muenchen	BOLD:AAM3906	658[0n]
Vespidae	Stenodynerus jurinei	BC ZSM HYM 04838	Italy	27-Jul-2007	SNSB, Zoologische Staatssammlung Muenchen	BOLD:AAM3906	658[0n]
Vespidae	Stenodynerus jurinei	BC ZSM HYM 04837	Italy	27-Jul-2007	SNSB, Zoologische Staatssammlung Muenchen	BOLD:AAM3906	658[0n]
Vespidae	Stenodynerus picticrus	BC-ZSM-HYM-27676-D06	Germany	10-Jun-2015	SNSB, Zoologische Staatssammlung Muenchen	BOLD:ADG3096	658[0n]
Vespidae	Stenodynerus punctifrons	BC ZSM HYM 06643	France	14-Jul-2010	SNSB, Zoologische Staatssammlung Muenchen	BOLD:AAU2628	658[0n]
Vespidae	Stenodynerus punctifrons	BC ZSM HYM 06631	France	14-Jul-2010	SNSB, Zoologische Staatssammlung Muenchen	BOLD:AAU2628	658[0n]
Vespidae	Stenodynerus punctifrons	BC ZSM HYM 06619	France	14-Jul-2010	SNSB, Zoologische Staatssammlung Muenchen	BOLD:AAU2628	658[0n]
Vespidae	Stenodynerus punctifrons	BC ZSM HYM 06607	France	15-Jul-2010	SNSB, Zoologische Staatssammlung Muenchen	BOLD:AAU2628	658[0n]
Vespidae	Stenodynerus steckianus	BC ZSM HYM 04842	Italy	14-Jul-2000	SNSB, Zoologische Staatssammlung Muenchen	BOLD:AAM4331	658[0n]
Vespidae	Stenodynerus steckianus	BC ZSM HYM 04840	Italy	13-May-2006	SNSB, Zoologische Staatssammlung Muenchen	BOLD:AAM4331	658[0n]
Vespidae	Stenodynerus xanthomelas	BC ZSM HYM 04843	Germany	10-Jun-2008	SNSB, Zoologische Staatssammlung Muenchen	BOLD:AAM3836	627[0n]
Vespidae	Stenodynerus xanthomelas	BC ZSM HYM 17381	Germany	18-Jul-2012	SNSB, Zoologische Staatssammlung Muenchen	BOLD:AAM3836	658[0n]
Vespidae	Stenodynerus xanthomelas	BC ZSM HYM 17380	Germany	18-Jul-2012	SNSB, Zoologische Staatssammlung Muenchen	BOLD:AAM3836	658[0n]
Vespidae	Stenodynerus xanthomelas	BC ZSM HYM 17382	Germany	18-Jul-2012	SNSB, Zoologische Staatssammlung Muenchen	BOLD:AAM3836	658[0n]
Vespidae	Stenodynerus xanthomelas	BC ZSM HYM 04786	Germany	02-Jul-2008	SNSB, Zoologische Staatssammlung Muenchen	BOLD:AAM3836	658[0n]
Vespidae	Stenodynerus xanthomelas	BC ZSM HYM 11897	Germany	03-Sep-2011	SNSB, Zoologische Staatssammlung Muenchen	BOLD:AAM3836	586[0n]
Vespidae	Stenodynerus xanthomelas	BC ZSM HYM 10221	Germany	16-Jul-2011	SNSB, Zoologische Staatssammlung Muenchen	BOLD:AAM3836	658[0n]
Vespidae	Stenodynerus xanthomelas	BC ZSM HYM 07409	Germany	07-Jul-2010	SNSB, Zoologische Staatssammlung Muenchen	BOLD:AAM3836	608[2n]
Vespidae	Symmorphus allobrogus	BC ZSM HYM 13977	Italy	09-Jul-2006	SNSB, Zoologische Staatssammlung Muenchen	BOLD:AAN0135	658[0n]
Vespidae	Symmorphus allobrogus	BC ZSM HYM 04941	Germany	23-Jul-2008	SNSB, Zoologische Staatssammlung Muenchen	BOLD:AAN0135	658[0n]
Vespidae	Symmorphus allobrogus	BC ZSM HYM 04943	Germany	01-Aug-2008	SNSB, Zoologische Staatssammlung Muenchen	BOLD:AAN0135	626[0n]
Vespidae	Symmorphus allobrogus	BC ZSM HYM 04942	Germany	02-Jul-2008	SNSB, Zoologische Staatssammlung Muenchen	BOLD:AAN0135	658[0n]
Vespidae	Symmorphus allobrogus	BC ZSM HYM 04845	Italy	09-Jul-2006	SNSB, Zoologische Staatssammlung Muenchen	BOLD:AAN0135	658[0n]
Vespidae	Symmorphus angustatus	BC ZSM HYM 13300	Germany	02-Jun-2009	SNSB, Zoologische Staatssammlung Muenchen	BOLD:ACL6133	658[0n]
Vespidae	Symmorphus bifasciatus	BC ZSM HYM 10634	Germany	19-Jul-2011	SNSB, Zoologische Staatssammlung Muenchen	BOLD:AAN3541	630[0n]
Vespidae	Symmorphus bifasciatus	BC ZSM HYM 10633	Germany	03-Sep-2011	SNSB, Zoologische Staatssammlung Muenchen	BOLD:AAN3541	658[0n]
Vespidae	Symmorphus bifasciatus	BC ZSM HYM 04947	Germany	02-Jul-2008	SNSB, Zoologische Staatssammlung Muenchen	BOLD:AAN3541	658[0n]
Vespidae	Symmorphus bifasciatus	BC ZSM HYM 04945	Germany	23-Jul-2006	SNSB, Zoologische Staatssammlung Muenchen	BOLD:AAN3541	658[0n]
Vespidae	Symmorphus bifasciatus	BC ZSM HYM 04944	Germany	23-Jul-2006	SNSB, Zoologische Staatssammlung Muenchen	BOLD:AAN3541	658[0n]
Vespidae	Symmorphus bifasciatus	BC ZSM HYM 10632	Germany	03-Sep-2011	SNSB, Zoologische Staatssammlung Muenchen	BOLD:AAN3541	658[0n]
Vespidae	Symmorphus connexus	BC ZSM HYM 19926	Italy	31-Jul-2013	SNSB, Zoologische Staatssammlung Muenchen	BOLD:AAN3693	658[0n]
Vespidae	Symmorphus connexus	BC ZSM HYM 04950	Germany	02-Jul-2008	SNSB, Zoologische Staatssammlung Muenchen	BOLD:AAN3693	658[0n]
Vespidae	Symmorphus connexus	BC ZSM HYM 04949	Germany	02-Jul-2008	SNSB, Zoologische Staatssammlung Muenchen	BOLD:AAN3693	658[0n]
Vespidae	Symmorphus connexus	BC ZSM HYM 04948	Germany	14-Aug-2008	SNSB, Zoologische Staatssammlung Muenchen	BOLD:AAN3693	658[0n]
Vespidae	Symmorphus crassicornis	BC ZSM HYM 10635	Germany	20-Jul-2011	SNSB, Zoologische Staatssammlung Muenchen	BOLD:AAN3694	658[0n]
Vespidae	Symmorphus crassicornis	BC ZSM HYM 04954	Germany	01-Jul-2008	SNSB, Zoologische Staatssammlung Muenchen	BOLD:AAN3694	614[0n]
Vespidae	Symmorphus crassicornis	BC ZSM HYM 04953	Germany	02-Jul-2008	SNSB, Zoologische Staatssammlung Muenchen	BOLD:AAN3694	658[0n]
Vespidae	Symmorphus crassicornis	BC ZSM HYM 04952	Germany	01-Jul-2008	SNSB, Zoologische Staatssammlung Muenchen	BOLD:AAN3694	658[0n]
Vespidae	Symmorphus crassicornis	BC ZSM HYM 04951	Germany	14-Aug-2008	SNSB, Zoologische Staatssammlung Muenchen	BOLD:AAN3694	658[0n]
Vespidae	Symmorphus debilitatus	BC ZSM HYM 17199	Germany	18-Jul-2012	SNSB, Zoologische Staatssammlung Muenchen	BOLD:AAN3537	658[0n]
Vespidae	Symmorphus debilitatus	BC ZSM HYM 04958	Germany	01-Jul-2008	SNSB, Zoologische Staatssammlung Muenchen	BOLD:AAN3537	658[0n]
Vespidae	Symmorphus debilitatus	BC ZSM HYM 04957	Germany	01-Jul-2008	SNSB, Zoologische Staatssammlung Muenchen	BOLD:AAN3537	658[0n]
Vespidae	Symmorphus debilitatus	BC ZSM HYM 04956	Germany	10-Jun-2008	SNSB, Zoologische Staatssammlung Muenchen	BOLD:AAN3537	658[0n]
Vespidae	Symmorphus debilitatus	BC ZSM HYM 04955	Germany	10-Jun-2008	SNSB, Zoologische Staatssammlung Muenchen	BOLD:AAN3537	658[0n]
Vespidae	Symmorphus gracilis	BC ZSM HYM 13975	Germany	28-Jul-2006	SNSB, Zoologische Staatssammlung Muenchen	BOLD:AAN3695	658[0n]
Vespidae	Symmorphus gracilis	BC ZSM HYM 13974	Germany	28-Jul-2006	SNSB, Zoologische Staatssammlung Muenchen	BOLD:AAN3695	658[0n]

Family	Species	Specimen ID	Country	Collection Date	Depository	BIN	COI-5P
Vespidae	Symmorphus gracilis	BC ZSM HYM 04963	Italy	17-Jul-2009	SNSB, Zoologische Staatssammlung Muenchen	BOLD:AAN3695	658[0n]
Vespidae	Symmorphus gracilis	BC ZSM HYM 04961	Germany	23-Jul-2008	SNSB, Zoologische Staatssammlung Muenchen	BOLD:AAN3695	622[0n]
Vespidae	Symmorphus gracilis	BC ZSM HYM 04960	Germany	02-Jul-2008	SNSB, Zoologische Staatssammlung Muenchen	BOLD:AAN3695	630[0n]
Vespidae	Symmorphus gracilis	BC ZSM HYM 04959	Germany	01-Jul-2008	SNSB, Zoologische Staatssammlung Muenchen	BOLD:AAN3695	658[0n]
Vespidae	Symmorphus gracilis	BC ZSM HYM 14702	Germany	21-May-2012	SNSB, Zoologische Staatssammlung Muenchen	BOLD:AAN3695	658[0n]
Vespidae	Symmorphus murarius	BC ZSM HYM 14405	Germany	30-Jun-2012	SNSB, Zoologische Staatssammlung Muenchen	BOLD:AAN3692	658[0n]
Vespidae	Symmorphus murarius	BC ZSM HYM 14404	Germany	30-Jun-2012	SNSB, Zoologische Staatssammlung Muenchen	BOLD:AAN3692	658[0n]
Vespidae	Symmorphus murarius	BC ZSM HYM 14403	Germany	30-Jun-2012	SNSB, Zoologische Staatssammlung Muenchen	BOLD:AAN3692	658[0n]
Vespidae	Symmorphus murarius	BC ZSM HYM 14402	Germany	30-Jun-2012	SNSB, Zoologische Staatssammlung Muenchen	BOLD:AAN3692	658[0n]
Vespidae	Symmorphus murarius	BC ZSM HYM 14401	Germany	30-Jun-2012	SNSB, Zoologische Staatssammlung Muenchen	BOLD:AAN3692	658[0n]
Vespidae	Symmorphus murarius	BC ZSM HYM 04967	Germany	10-Jun-2008	SNSB, Zoologische Staatssammlung Muenchen	BOLD:AAN3692	658[0n]
Vespidae	Symmorphus murarius	BC ZSM HYM 04966	Germany	01-Jul-2008	SNSB, Zoologische Staatssammlung Muenchen	BOLD:AAN3692	658[0n]
Vespidae	Symmorphus murarius	BC ZSM HYM 04965	Germany	27-Jun-2006	SNSB, Zoologische Staatssammlung Muenchen	BOLD:AAN3692	622[0n]
Vespidae	Symmorphus murarius	BC ZSM HYM 04964	Germany	27-Jun-2006	SNSB, Zoologische Staatssammlung Muenchen	BOLD:AAN3692	658[0n]
Vespidae	Vespa crabro	BC ZSM HYM 13037	Germany	10-Oct-2010	SNSB, Zoologische Staatssammlung Muenchen	BOLD:ABA8441	658[0n]
Vespidae	Vespa crabro	BC ZSM HYM 13038	Germany	10-Oct-2010	SNSB, Zoologische Staatssammlung Muenchen	BOLD:ABA8441	658[0n]
Vespidae	Vespa crabro	BC ZSM HYM 13036	Germany	10-Oct-2010	SNSB, Zoologische Staatssammlung Muenchen	BOLD:ABA8441	658[0n]
Vespidae	Vespa crabro	BC ZSM HYM 15275	Germany	04-Aug-2012	SNSB, Zoologische Staatssammlung Muenchen	BOLD:ABA8441	658[0n]
Vespidae	Vespa crabro	BC ZSM HYM 14389	Germany	30-Jun-2012	SNSB, Zoologische Staatssammlung Muenchen	BOLD:ABA8441	658[0n]
Vespidae	Vespa crabro	BC ZSM HYM 14388	Germany	30-Jun-2012	SNSB, Zoologische Staatssammlung Muenchen	BOLD:ABA8441	658[0n]
Vespidae	Vespa crabro	BC ZSM HYM 14387	Germany	30-Jun-2012	SNSB, Zoologische Staatssammlung Muenchen	BOLD:ABA8441	658[0n]
Vespidae	Vespa crabro	GBOL09095	Hungary	06-Sep-2013	SNSB, Zoologische Staatssammlung Muenchen	BOLD:ABA8441	658[0n]
Vespidae	Vespa orientalis	BC ZSM HYM 21139	Cyprus	20-Jun-2013	SNSB, Zoologische Staatssammlung Muenchen	BOLD:ACR5399	658[0n]
Vespidae	Vespa orientalis	BC ZSM HYM 21138	Cyprus	20-Jun-2013	SNSB, Zoologische Staatssammlung Muenchen	BOLD:ACR5399	658[0n]
Vespidae	Vespa velutina	BC ZSM HYM 10631	France	02-Oct-2007	SNSB, Zoologische Staatssammlung Muenchen	BOLD:AAQ3010	658[0n]
Vespidae	Vespa velutina	BC ZSM HYM 10628	France	01-Oct-2007	SNSB, Zoologische Staatssammlung Muenchen	BOLD:AAQ3010	658[0n]
Vespidae	Vespa velutina	BC ZSM HYM 10630	France	01-Oct-2007	SNSB, Zoologische Staatssammlung Muenchen	BOLD:AAQ3010	658[0n]
Vespidae	Vespa velutina	BC ZSM HYM 10629	France	01-Oct-2007	SNSB, Zoologische Staatssammlung Muenchen	BOLD:AAQ3010	658[0n]
Vespidae	Vespula austriaca	BC ZSM HYM 05028	Germany	01-Jul-2008	SNSB, Zoologische Staatssammlung Muenchen	BOLD:AAN3441	658[0n]
Vespidae	Vespula austriaca	BC ZSM HYM 05025	Germany	01-Aug-2007	SNSB, Zoologische Staatssammlung Muenchen	BOLD:AAN3441	658[0n]
Vespidae	Vespula austriaca	BC ZSM HYM 05027	Germany	01-Jul-2008	SNSB, Zoologische Staatssammlung Muenchen	BOLD:AAN3441	658[0n]
Vespidae	Vespula austriaca	BC ZSM HYM 05026	Germany	01-Jul-2008	SNSB, Zoologische Staatssammlung Muenchen	BOLD:AAN3441	658[0n]
Vespidae	Vespula austriaca	BC ZSM HYM 17209	Germany	05-Aug-2007	SNSB, Zoologische Staatssammlung Muenchen	BOLD:AAN3441	658[0n]
Vespidae	Vespula germanica	BC ZSM HYM 17208	Iran	26-Sep-2008	SNSB, Zoologische Staatssammlung Muenchen	BOLD:AAG9055	658[0n]
Vespidae	Vespula germanica	BC ZSM HYM 15270	Germany	28-Aug-2012	SNSB, Zoologische Staatssammlung Muenchen	BOLD:AAG9055	658[0n]
Vespidae	Vespula germanica	BC ZSM HYM 15269	Germany	20-Aug-2012	SNSB, Zoologische Staatssammlung Muenchen	BOLD:AAG9055	658[0n]
Vespidae	Vespula germanica	BC ZSM HYM 15268	Germany	12-Aug-2012	SNSB, Zoologische Staatssammlung Muenchen	BOLD:AAG9055	658[0n]
Vespidae	Vespula germanica	BC ZSM HYM 15267	Germany	10-Aug-2012	SNSB, Zoologische Staatssammlung Muenchen	BOLD:AAG9055	658[0n]
Vespidae	Vespula germanica	BC ZSM HYM 13991	Germany	16-Jul-2011	SNSB, Zoologische Staatssammlung Muenchen	BOLD:AAG9055	658[0n]
Vespidae	Vespula germanica	BC ZSM HYM 10199	Germany	16-Jul-2011	SNSB, Zoologische Staatssammlung Muenchen	BOLD:AAG9055	658[0n]
Vespidae	Vespula germanica	BC ZSM HYM 10198	Germany	16-Jul-2011	SNSB, Zoologische Staatssammlung Muenchen	BOLD:AAG9055	658[0n]
Vespidae	Vespula germanica	BC ZSM HYM 10197	Germany	16-Jul-2011	SNSB, Zoologische Staatssammlung Muenchen	BOLD:AAG9055	658[0n]
Vespidae	Vespula germanica	BC ZSM HYM 10196	Germany	16-Jul-2011	SNSB, Zoologische Staatssammlung Muenchen	BOLD:AAG9055	658[0n]
Vespidae	Vespula germanica	BC ZSM HYM 06246	Germany	10-Aug-2010	SNSB, Zoologische Staatssammlung Muenchen	BOLD:AAG9055	658[0n]
Vespidae	Vespula germanica	BC ZSM HYM 08255	Germany	19-Apr-2011	SNSB, Zoologische Staatssammlung Muenchen	BOLD:AAG9055	658[0n]
Vespidae	Vespula rufa	BC ZSM HYM 13972	Germany	07-May-2006	SNSB, Zoologische Staatssammlung Muenchen	BOLD:AAG0744	658[0n]
Vespidae	Vespula rufa	BC ZSM HYM 13971	Germany	07-May-2006	SNSB, Zoologische Staatssammlung Muenchen	BOLD:AAG0744	658[0n]

Family	Species	Specimen ID	Country	Collection Date	Depository	BIN	COI-5P
Vespidae	Vespula rufa	BC ZSM HYM 05032	Italy	27-Jul-2007	SNSB, Zoologische Staatssammlung Muenchen	BOLD:AAG0744	658[0n]
Vespidae	Vespula rufa	BC ZSM HYM 05031	Germany	19-Aug-2006	SNSB, Zoologische Staatssammlung Muenchen	BOLD:AAG0744	658[0n]
Vespidae	Vespula rufa	BC ZSM HYM 05030	Germany	05-Aug-2007	SNSB, Zoologische Staatssammlung Muenchen	BOLD:AAG0744	622[0n]
Vespidae	Vespula rufa	BC ZSM HYM 05029	Germany	01-May-2007	SNSB, Zoologische Staatssammlung Muenchen	BOLD:AAG0744	658[0n]
Vespidae	Vespula rufa	BC ZSM HYM 10200	Germany	16-Jul-2011	SNSB, Zoologische Staatssammlung Muenchen	BOLD:AAG0744	658[0n]
Vespidae	Vespula vulgaris	BC ZSM HYM 14022	Germany	16-Jul-2011	SNSB, Zoologische Staatssammlung Muenchen	BOLD:AAM2781	658[0n]
Vespidae	Vespula vulgaris	BC ZSM HYM 14020	Germany	16-Jul-2011	SNSB, Zoologische Staatssammlung Muenchen	BOLD:AAM2781	658[0n]
Vespidae	Vespula vulgaris	BC ZSM HYM 14019	Germany	16-Jul-2011	SNSB, Zoologische Staatssammlung Muenchen	BOLD:AAM2781	658[0n]
Vespidae	Vespula vulgaris	BC ZSM HYM 16991	Germany	18-Jul-2012	SNSB, Zoologische Staatssammlung Muenchen	BOLD:AAM2781	658[0n]
Vespidae	Vespula vulgaris	BC ZSM HYM 14021	Germany	16-Jul-2011	SNSB, Zoologische Staatssammlung Muenchen	BOLD:AAM2781	658[0n]
Vespidae	Vespula vulgaris	BC ZSM HYM 05052	Germany	10-May-2010	SNSB, Zoologische Staatssammlung Muenchen	BOLD:AAM2781	658[0n]
Vespidae	Vespula vulgaris	BC ZSM HYM 05051	Germany	15-May-2008	SNSB, Zoologische Staatssammlung Muenchen	BOLD:AAM2781	658[0n]
Vespidae	Vespula vulgaris	BC ZSM HYM 05050	Germany	01-May-2007	SNSB, Zoologische Staatssammlung Muenchen	BOLD:AAM2781	658[0n]
Vespidae	Vespula vulgaris	BC ZSM HYM 05049	Germany	01-Jul-2008	SNSB, Zoologische Staatssammlung Muenchen	BOLD:AAM2781	658[0n]
Vespidae	Vespula vulgaris	BC ZSM HYM 05048	Germany	05-Aug-2007	SNSB, Zoologische Staatssammlung Muenchen	BOLD:AAM2781	658[0n]
Vespidae	Vespula vulgaris	BC ZSM HYM 05047	Austria	06-Aug-2007	SNSB, Zoologische Staatssammlung Muenchen	BOLD:AAM2781	658[0n]
Vespidae	Vespula vulgaris	BC ZSM HYM 17210	Germany	05-Aug-2007	SNSB, Zoologische Staatssammlung Muenchen	BOLD:AAM2781	658[0n]
Vespidae	Vespula vulgaris	BC ZSM HYM 15274	Germany	12-Aug-2012	SNSB, Zoologische Staatssammlung Muenchen	BOLD:AAM2781	658[0n]
Vespidae	Vespula vulgaris	BC ZSM HYM 15273	Germany	12-Aug-2012	SNSB, Zoologische Staatssammlung Muenchen	BOLD:AAM2781	658[0n]
Vespidae	Vespula vulgaris	BC ZSM HYM 15272	Germany	01-Aug-2012	SNSB, Zoologische Staatssammlung Muenchen	BOLD:AAM2781	658[0n]
Vespidae	Vespula vulgaris	BC ZSM HYM 15271	Germany	04-Aug-2012	SNSB, Zoologische Staatssammlung Muenchen	BOLD:AAM2781	658[0n]
Vespidae	Vespula vulgaris	BC ZSM HYM 06245	Germany	10-Aug-2010	SNSB, Zoologische Staatssammlung Muenchen	BOLD:AAM2781	658[0n]
