# Supplementary material for: Unexpected diversity in Central European Vespoidea (Hymenoptera, Mutillidae, Myrmosidae, Sapygidae, Scoliidae, Tiphiidae, Thynnidae, Vespidae), with description of two species of Smicromyrme Thomson, 1870
Source: Zookeys. 2021 Oct 14;1062:49–72. doi: 10.3897/zookeys.1062.70763 (PMC8530993; doi:10.3897/zookeys.1062.70763)
Supplement: Supplementary material 2 — DNA barcoding statistics [file zookeys-1062-049-s002.pdf]

Family	Species	Mean	Max	Nearest species	NN distance	BIN	Country	Specimens
Mutillidae	Dasylabris maura	N/A	0.00	Eumenes mediterraneus	32.55		Germany	2
						BOLD:ABA1491	Italy	1
	Mutilla europaea	N/A	N/A	N/A			France	3
	Mutilla marginata	N/A	N/A	N/A	N/A		Germany	1
	Myrmilla calva	0.41	0.62	Myrmilla erythrocephala	17.71	BOLD:AAU2040	Croatia	1
							Italy	2
	Myrmilla erythrocephala	N/A	0.00	Myrmilla calva	17.71	BOLD:ABA8661	France	1
							Italy	1
	Physetopoda daghestanica	N/A	N/A	N/A	N/A		Austria	1
							Hungary	2
	Physetopoda halensis	1.55	2.92	Smicromyrme lombardensis	24.93	BOLD:ABV4512	Germany	3
						BOLD:ACC4310	Czech Republic	1
	Physetopoda scutellaris	N/A	N/A	N/A	N/A		Germany	7
	Smicromyrme burgeri	0.67	2.20	Smicromyrme lombardensis	5.65	BOLD:AAU3642	France	1
							Germany	10
	Smicromyrme lombardensis	0.54	0.83	Smicromyrme burgeri	5.65	BOLD:ABU8217	Italy	3
	Smicromyrme rufipes	0.33	0.68	Smicromyrme lombardensis	13.69	BOLD:AAU3641	Germany	21
	Stenomutilla argentata	1.09	1.09	Myrmilla erythrocephala	31.83	BOLD:ABV3984	France	2
Myrmosidae	Krombeinella thoracica	14.49	14.49	Paramyrmosa brunnipes	26.28	BOLD:ABV3985	Croatia	1
						BOLD:ABV3986	Italy	1
	Myrmosa atra	3.33	8.78	Paramyrmosa brunnipes	24.08	BOLD:AAU3504	Germany	23
							Slovakia	1
						BOLD:AAY9719	Germany	10
	Paramyrmosa brunnipes	N/A	0.00	Myrmosa atra	24.08	BOLD:AAV3086	France	1
Sapygidae	Monosapyga clavicornis	0.29	0.77	Sapygina decemguttata	14.29	BOLD:AAU3221	Austria	2
							Germany	6
	Sapyga quinquepunctata	0.32	0.62	Sapyga similis	11.40	BOLD:AAV7700	Czech Republic	1
							Germany	2
							Italy	1
							Spain	2
	Sapyga similis	1.24	1.24	Sapyga quinquepunctata	11.40	BOLD:AAV7701	France	1
							Germany	1
	Sapygina decemguttata	0.05	0.35	Sapyga similis	12.83	BOLD:AAO1197	France	1
							Germany	12
Scoliidae	Campsomeriella thoracica	2.83	5.3	Megascolia maculata	19.34	BOLD:ADY3101	Egypt	2
						BOLD:ADY5638	Morocco	1
							Tunisia	3
						BOLD:ADY5639	United Arab Emirates	3
	Colpa quinquecincta	1.02	1.71	Colpa sexmaculata	14.05	BOLD:ACG2231	Italy	5
	Colpa sexmaculata	0.59	1.16	Megascolia bidens	10.97	BOLD:ADM7341	Croatia	1
							Italy	3

Family	Species	Mean	Max	Nearest species	NN distance	BIN	Country	Specimens
	Megascolia bidens	0.00	0.00	Megascolia maculata	3.38	BOLD:AEC9507	Spain	2
	Megascolia maculata	0.43	1.70	Megascolia bidens	3.38	BOLD:AAV7442	Cyprus	1
							Hungary	1
							Italy	1
							Spain	3
							Turkey	2
	Micromeriella aureola	0.15	0.31	Micromeriella hyalina	19.89	BOLD:ACO4425	Tunisia	4
	Micromeriella hyalina	5.40	10.07	Scolia sexmaculata	18.11	BOLD:ACO5284	United Arab Emirates	2
						BOLD:ACO5576	United Arab Emirates	2
						BOLD:ACO5577	United Arab Emirates	1
						BOLD:ADX9764	Morocco	3
	Scolia hirta	0.07	0.19	Scolia hortorum	6.22	BOLD:AAU2351	Germany	4
							Italy	6
	Scolia hortorum	4.75	4.75	Scolia miniata	2.47	BOLD:AAU2353	France	1
						BOLD:ACG2158	Tunisia	1
	Scolia miniata	N/A	0.00	Scolia hortorum	2.47	BOLD:ACG2159	United Arab Emirates	1
	Scolia sexmaculata	0.44	1.12	Scolia hortorum	3.29	BOLD:AAU2352	France	3
							Germany	11
							Italy	4
Thynnidae	Meria cylindrica	6.93	13.24	Meria tripunctata	9.47	BOLD:ACG1615	France	4
							Italy	1
						BOLD:ACG2533	France	9
						BOLD:ADA9671	France	1
	Meria tripunctata	3.61	6.05	Meria cylindrica	9.47	BOLD:AAY6941	Italy	3
						BOLD:ADX9865	Italy	3
	Methocha articulata	0.73	1.48	Eumenes dubius	26.19	BOLD:AAU3521	Croatia	1
							Czech Republic	1
							Germany	2
	Poecilotiphia rousseli	0.00	0.00	Meria tripunctata	15.07	BOLD:ACM1581	France	2
Tiphiidae	Tiphia femorata	4.48	13.05	Tiphia minuta	12.89	BOLD:AAP1102	Germany	9
							Hungary	1
						BOLD:AAP1103	Germany	51
							Romania	5
						BOLD:AAY9685	Germany	16
							Hungary	1
						BOLD:ABA9329	Germany	5
							Italy	2
						BOLD:ADV9902	France	2
	Tiphia minuta	0.18	0.31	Tiphia femorata	12.89	BOLD:AAU3783	Germany	5
	Tiphia unicolor	0.40	1.14	Tiphia femorata	13.85	BOLD:AAU2938	Germany	19
							Italy	2

Family	Species	Mean	Max	Nearest species	NN distance	BIN	Country	Specimens
	Tiphia villosa	0.08	0.16	Tiphia femorata	19.48	BOLD:AAU2941	Germany	4
Vespidae	Alastor atropos	0.00	0.00	Alastor biegelebeni	11.17	BOLD:ACG1916	Germany	2
	Alastor biegelebeni	N/A	0.00	Alastor atropos	11.17	BOLD:ADH3858	Czech Republic	1
	Allodynerus delphinalis	0.85	1.39	Euodynerus notatus	17.51	BOLD:AAM4849	Germany	3
							Italy	1
	Allodynerus rossii	0.41	0.46	Euodynerus notatus	17.47	BOLD:AAM3835	Germany	3
	Ancistrocerus antilope	0.00	0.00	Ancistrocerus longispinosus	9.97	BOLD:AAM2254	Germany	5
	Ancistrocerus auctus	0.11	0.16	Ancistrocerus renimacula	9.14	BOLD:AAM4699	Italy	3
	Ancistrocerus biphaleratus	N/A	0.00	Ancistrocerus longispinosus	8.66	BOLD:ACV7686	Italy	1
	Ancistrocerus claripennis	0.14	0.31	Ancistrocerus trifasciatus	6.42	BOLD:AAM3573	Germany	3
							Italy	3
	Ancistrocerus dusmetiolus	N/A	0.00	Ancistrocerus parietum	6.25	BOLD:ACP4668	Germany	1
	Ancistrocerus gazella	0.00	0.00	Ancistrocerus trifasciatus	6.07	BOLD:AAF4164	Germany	6
							Italy	2
	Ancistrocerus ichneumonideus	0.15	0.15	Ancistrocerus longispinosus	7.23	BOLD:ACC4131	Germany	2
	Ancistrocerus longispinosus	0.10	0.16	Ancistrocerus trifasciatus	4.01	BOLD:ACV8574	Cyprus	2
							Switzerland	1
	Ancistrocerus nigricornis	0.02	0.15	Ancistrocerus longispinosus	7.90	BOLD:AAM3899	Germany	19
	Ancistrocerus oviventris	0.10	0.31	Ancistrocerus longispinosus	6.56	BOLD:AAJ2022	Germany	7
							Italy	6
	Ancistrocerus parietinus	N/A	0.00	Ancistrocerus longispinosus	8.75	BOLD:AAM4868	Poland	1
	Ancistrocerus parietum	0.15	0.15	Ancistrocerus dusmetiolus	6.25	BOLD:AAM4869	Germany	2
	Ancistrocerus renimacula	N/A	0.00	Ancistrocerus auctus	9.14	BOLD:AAM4859	France	1
	Ancistrocerus trifasciatus	0.28	0.77	Ancistrocerus longispinosus	4.01	BOLD:AAM3937	Austria	1
							Germany	5
							Italy	1
	Antepipona deflenda	0.00	0.00	Euodynerus quadrifasciatus	16.34	BOLD:ADL0396	Cyprus	2
	Antepipona orbitalis	0.00	0.00	Ancistrocerus oviventris	15.96	BOLD:ACC2791	Hungary	4
	Celonites abbreviatus	1.93	3.68	Celonites mayeti	14.16	BOLD:AAP2382	Austria	1
							Germany	1
							Italy	2
							Switzerland	3
						BOLD:AAP2383	Italy	3
	Celonites mayeti	N/A	0.00	Celonites abbreviatus	14.16	BOLD:ACG0764	France	1
	Celonites rugiceps	0.34	0.34	Celonites mayeti	19.11	BOLD:ACX5359	Greece	2
	Ceramius tuberculifer	N/A	0.00	Celonites rugiceps	25.38	BOLD:ACX4794	France	1
	Delta unguiculatum	N/A	0.00	Eumenes pedunculatus	12.04	BOLD:AAN1374	Italy	1
	Discoelius dufourii	N/A	0.00	Discoelius zonalis	18.49	BOLD:AAN1415	Germany	1
	Discoelius zonalis	0.28	0.64	Ancistrocerus parietinus	16.94	BOLD:AAY8844	Germany	8
	Dolichovespula adulterina	0.33	0.49	Dolichovespula omissa	4.07	BOLD:AAI6347	Germany	5
	Dolichovespula media	0.00	0.00	Dolichovespula sylvestris	12.63	BOLD:AAB6388	Germany	3

Family	Species	Mean	Max	Nearest species	NN distance	BIN	Country	Specimens
	Dolichovespula norwegica	0.08	0.31	Dolichovespula pacifica	3.93	BOLD:AAA6640	Germany	5
							Italy	3
	Dolichovespula omissa	0.21	0.32	Dolichovespula adulterina	4.07	BOLD:AAN4303	Germany	3
	Dolichovespula pacifica	0.00	0.00	Dolichovespula norwegica	3.93	BOLD:ACL8383	Sweden	2
	Dolichovespula saxonica	0.25	0.46	Dolichovespula adulterina	6.22	BOLD:AAM3988	Germany	8
	Dolichovespula sylvestris	1.52	3.46	Dolichovespula norwegica	6.71	BOLD:AAM3989	Germany	8
						BOLD:AAM3990	Italy	1
						BOLD:AAM3991	France	2
	Eumenes coarctatus	1.63	3.78	Eumenes pedunculatus	6.52	BOLD:AAN3538	Germany	10
						BOLD:AAN4043	Cyprus	4
						BOLD:ACE4606	France	2
							Italy	7
	Eumenes coronatus	0.32	0.77	Eumenes subpomiformis	5.71	BOLD:AAN3639	Germany	17
	Eumenes dubius	3.81	5.72	Eumenes sareptanus	6.23	BOLD:ACR5018	Cyprus	2
						BOLD:ACV9421	Spain	2
	Eumenes mediterraneus	0.00	0.00	Eumenes subpomiformis	7.90	BOLD:ACR5439	Cyprus	2
	Eumenes papillarius	0.00	0.00	Eumenes coronatus	5.88	BOLD:AAY8811	Germany	10
							Italy	1
	Eumenes pedunculatus	0.02	0.17	Eumenes coarctatus	6.52	BOLD:AAL4355	Germany	16
	Eumenes pomiformis	1.16	5.28	Eumenes subpomiformis	6.24	BOLD:AAN4044	Italy	8
						BOLD:ACV9324	Kyrgyzstan	1
	Eumenes sareptanus	0.16	0.46	Eumenes dubius	6.23	BOLD:ABA9380	Germany	4
							Hungary	3
							Italy	1
							Spain	1
	Eumenes subpomiformis	N/A	0.00	Eumenes coronatus	5.71	BOLD:ACG1686	France	1
	Euodynerus dantici	0.46	0.46	Euodynerus disconotatus	12.36	BOLD:AAM5315	France	2
	Euodynerus disconotatus	0.22	0.34	Euodynerus dantici	12.36	BOLD:ADD0366	Cyprus	3
	Euodynerus notatus	0.05	0.15	Euodynerus quadrifasciatus	5.39	BOLD:AAN0689	Germany	12
	Euodynerus quadrifasciatus	0.66	1.08	Euodynerus notatus	5.39	BOLD:ABY9039	France	2
							Germany	2
							Italy	2
	Gymnomerus laevipes	0.02	0.15	Pterocheilus phaleratus	14.83	BOLD:AAL3407	Germany	14
	Katamenes arbustorum	0.70	1.39	Eumenes dubius	13.90	BOLD:AAM6807	France	2
							Italy	2
	Leptochilus alpestris	6.28	9.45	Leptochilus regulus	11.91	BOLD:AAM9217	France	1
							Italy	1
						BOLD:AAM9218	France	2
	Leptochilus moustirsensis	N/A	0.00	Leptochilus tarsatus	12.45	BOLD:ACG1074	France	1
	Leptochilus regulus	0.62	0.62	Leptochilus alpestris	11.91	BOLD:AAM9219	France	2
	Leptochilus tarsatus	0.10	0.15	Leptochilus moustirsensis	12.45	BOLD:AAM4311	Italy	3

Family	Species	Mean	Max	Nearest species	NN distance	BIN	Country	Specimens
	Microdynerus exilis	N/A					Germany	4
	Microdynerus longicollis	N/A	0.00	Microdynerus parvulus	13.54	BOLD:AAN2278	Germany	1
	Microdynerus nugdunensis	N/A					Germany	1
	Microdynerus parvulus	0.06	0.16	Microdynerus timidus	11.74	BOLD:AAM3885	Germany	4
							Italy	1
	Microdynerus timidus	0.08	0.15	Microdynerus parvulus	11.74	BOLD:AAY8818	Germany	6
	Odynerus alpinus	0.32	0.63	Odynerus spinipes	8.11	BOLD:AAN1663	France	3
							Germany	1
	Odynerus dusmeticus	0.00	0.00	Odynerus melanocephalus	1.17	BOLD:ADE9903	Spain	2
	Odynerus melanocephalus	0.06	0.31	Odynerus dusmeticus	1.17	BOLD:AAW9811	France	1
							Germany	10
	Odynerus reniformis	0.88	1.40	Odynerus dusmeticus	14.85	BOLD:AAV3568	France	2
							Germany	2
	Odynerus spinipes	0.28	0.62	Odynerus alpinus	8.11	BOLD:AAK2980	Germany	6
							Poland	1
	Polistes albellus	0.00	0.00	Polistes bischoffi	2.66	BOLD:AAN3553	Germany	2
							Switzerland	1
	Polistes atrimandibularis	0.00	0.00	Polistes semenowi	1.70	BOLD:AAN4297	Italy	2
	Polistes austroccidentalis	0.72	0.77	Polistes atrimandibularis	2.18	BOLD:ACG1677	France	1
							Morocco	1
							Switzerland	1
	Polistes biglumis	0.72	1.08	Polistes bischoffi	2.49	BOLD:AAN3552	Germany	2
							Italy	1
	Polistes bischoffi	0.00	0.00	Polistes biglumis	2.49	BOLD:ACG2292	Croatia	1
							Switzerland	2
	Polistes dominula	2.63	4.43	Polistes nimpha	4.66	BOLD:AAA9495	France	1
							Germany	2
						BOLD:AAB7105	Germany	2
							Italy	1
	Polistes gallicus	0.10	0.15	Polistes biglumis	2.49	BOLD:AAN3302	Croatia	1
							Italy	1
							Spain	1
	Polistes nimpha	3.34	5.75	Polistes dominula	4.66	BOLD:AAL0103	Germany	3
						BOLD:ACC1661	Germany	3
	Polistes semenowi	0.20	0.31	Polistes atrimandibularis	1.70	BOLD:ACG1290	Italy	2
							Switzerland	1
	Pterocheilus phaleratus	0.17	0.46	Gymnomerus laevipes	14.83	BOLD:AAL2208	Germany	13
	Stenodynerus bluethgeni	0.34	0.65	Stenodynerus steckianus	8.26	BOLD:AAM4330	France	1
							Germany	9
							Italy	3
	Stenodynerus chevrieranus	0.95	1.42	Stenodynerus xanthomelas	15.09	BOLD:AAN0040	Germany	1

Family	Species	Mean	Max	Nearest species	NN distance	BIN	Country	Specimens
							Slovakia	1
							Switzerland	1
	Stenodynerus clypeopictus	N/A	0.00	Stenodynerus xanthomelas	17.27	BOLD:ADH6201	Slovakia	1
	Stenodynerus jurinei	0.28	0.62	Stenodynerus steckianus	8.63	BOLD:AAM3906	Germany	1
							Italy	4
	Stenodynerus picticrus	N/A	0.00	Stenodynerus steckianus	8.09	BOLD:ADG3096	Germany	1
	Stenodynerus punctifrons	0.08	0.15	Stenodynerus steckianus	9.12	BOLD:AAU2628	France	4
	Stenodynerus steckianus	0.31	0.31	Stenodynerus picticrus	8.09	BOLD:AAM4331	Italy	2
	Stenodynerus xanthomelas	0.07	0.17	Stenodynerus chevrieranus	15.09	BOLD:AAM3836	Germany	8
	Symmorphus allobrogus	0.50	0.98	Symmorphus connexus	12.49	BOLD:AAN0135	Germany	3
							Italy	2
	Symmorphus angustatus	N/A	0.00	Symmorphus crassicornis	1.70	BOLD:ACL6133	Germany	1
	Symmorphus bifasciatus	0.10	0.31	Symmorphus debilitatus	13.06	BOLD:AAN3541	Germany	6
	Symmorphus connexus	0.08	0.15	Symmorphus allobrogus	12.49	BOLD:AAN3693	Germany	3
							Italy	1
	Symmorphus crassicornis	0.00	0.00	Symmorphus angustatus	1.70	BOLD:AAN3694	Germany	5
	Symmorphus debilitatus	0.15	0.31	Symmorphus bifasciatus	13.06	BOLD:AAN3537	Germany	5
	Symmorphus gracilis	0.14	0.32	Symmorphus allobrogus	13.59	BOLD:AAN3695	Germany	6
							Italy	1
	Symmorphus murarius	0.17	0.46	Symmorphus debilitatus	14.87	BOLD:AAN3692	Germany	9
	Vespa crabro	0.27	1.08	Vespa velutina	13.94	BOLD:ABA8441	Germany	7
							Hungary	1
	Vespa orientalis	0.15	0.15	Vespa crabro	14.86	BOLD:ACR5399	Cyprus	2
	Vespa velutina	0.00	0.00	Vespa crabro	13.94	BOLD:AAQ3010	France	4
	Vespula austriaca	0.00	0.00	Vespula rufa	5.40	BOLD:AAN3441	Germany	5
	Vespula germanica	0.20	0.62	Vespula vulgaris	8.61	BOLD:AAG9055	Germany	11
							Iran	1
	Vespula rufa	0.17	0.46	Vespula austriaca	5.40	BOLD:AAG0744	Germany	6
							Italy	1
	Vespula vulgaris	0.36	0.62	Vespula germanica	8.61	BOLD:AAM2781	Austria	1
							Germany	16
