# Supplementary material for: Unexpected diversity in Central European Vespoidea (Hymenoptera, Mutillidae, Myrmosidae, Sapygidae, Scoliidae, Tiphiidae, Thynnidae, Vespidae), with description of two species of Smicromyrme Thomson, 1870
Source: Zookeys. 2021 Oct 14;1062:49–72. doi: 10.3897/zookeys.1062.70763 (PMC8530993; doi:10.3897/zookeys.1062.70763)
Supplement: Supplementary material 3 — Neighbor-joining tree resulting from the analysis of DNA barcode sequences [file zookeys-1062-049-s003.pdf]

BOLD TaxonID Tree

Title	:	Tree Result - DS-GBVEOCE
Date	:	28-Jun-2021
Data Type	:	Nucleotide
Distance Model	:	Kimura 2 Parameter
Marker	:	COI-5P
Colourization	:	Barcode Cluster (BIN)

Label	:	Sample ID
Label	:	Taxon
Label	:	Sex/Gender
Label	:	Country
Label	:	Province/State
Label	:	Barcode Cluster (BIN)

Sequence Count	:	868
Species count	:	134
Genus count	:	43
Family count	:	7
Unidentified	:	0

BIN Count	:	154

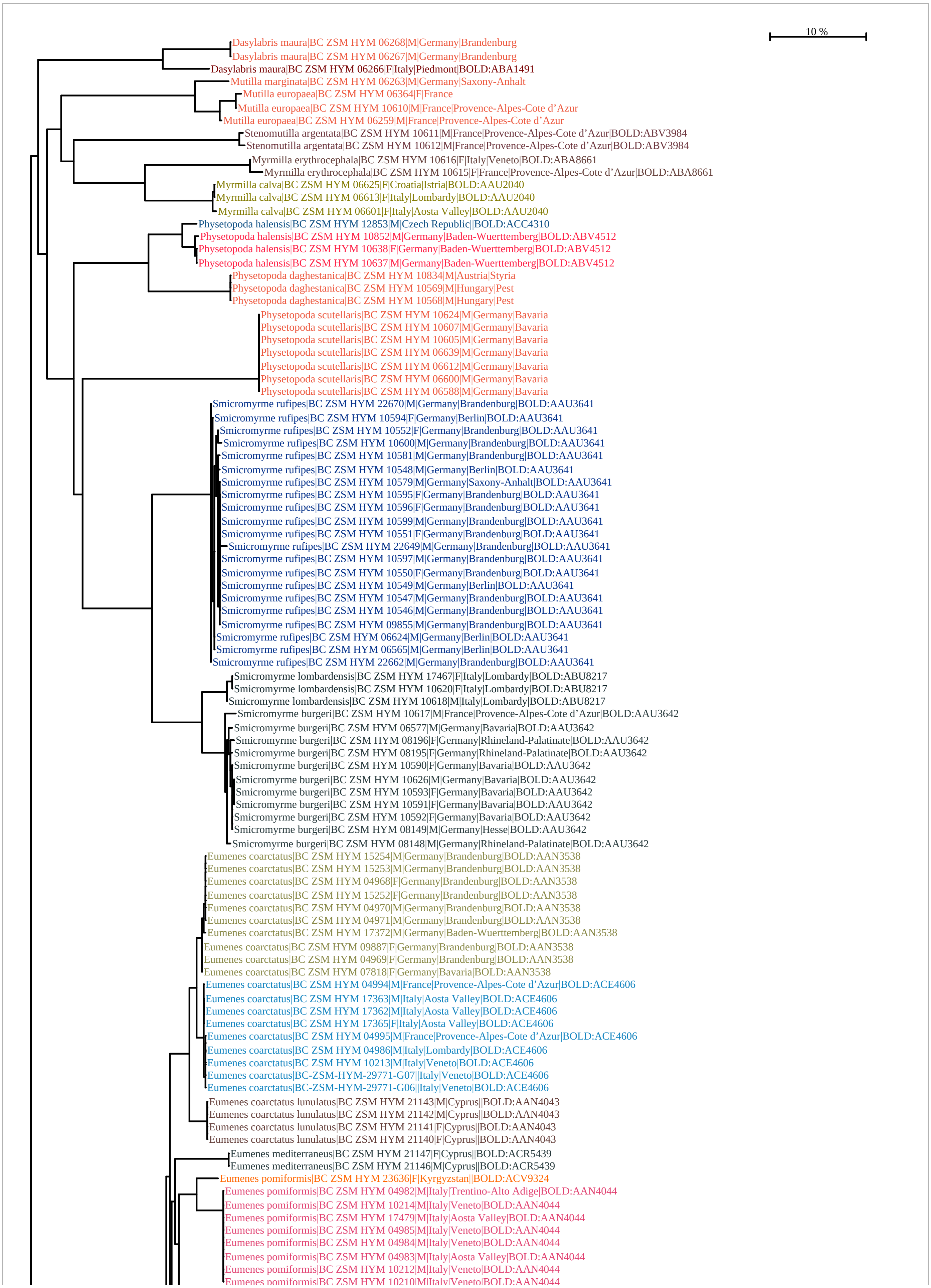
10 %
Dasylabris maura|BC ZSM HYM 06268|M|Germany|Brandenburg
Dasylabris maura|BC ZSM HYM 06267|M|Germany|Brandenburg
Dasylabris maura|BC ZSM HYM 06266|F|Italy|Piedmont|BOLD:ABA1491
Mutilla marginata|BC ZSM HYM 06263|M|Germany|Saxony-Anhalt
Mutilla europaea|BC ZSM HYM 06364|F|France
Mutilla europaea|BC ZSM HYM 10610|M|France|Provence-Alpes-Cote d'Azur
Mutilla europaea|BC ZSM HYM 06259|M|France|Provence-Alpes-Cote d'Azur
Stenomutilla argentata|BC ZSM HYM 10611|M|France|Provence-Alpes-Cote d'Azur|BOLD:ABV3984
Stenomutilla argentata|BC ZSM HYM 10612|M|France|Provence-Alpes-Cote d'Azur|BOLD:ABV3984
Myrmilla erythrocephala|BC ZSM HYM 10616|F|Italy|Veneto|BOLD:ABA8661
Myrmilla erythrocephala|BC ZSM HYM 10615|F|France|Provence-Alpes-Cote d'Azur|BOLD:ABA8661
Myrmilla calva|BC ZSM HYM 06625|F|Croatia|Istria|BOLD:AAU2040
Myrmilla calva|BC ZSM HYM 06613|F|Italy|Lombardy|BOLD:AAU2040
Myrmilla calva|BC ZSM HYM 06601|F|Italy|Aosta Valley|BOLD:AAU2040
Physetopoda halensis|BC ZSM HYM 12853|M|Czech Republic||BOLD:ACC4310
Physetopoda halensis|BC ZSM HYM 10852|M|Germany|Baden-Wuerttemberg|BOLD:ABV4512
Physetopoda halensis|BC ZSM HYM 10638|F|Germany|Baden-Wuerttemberg|BOLD:ABV4512
Physetopoda halensis|BC ZSM HYM 10637|M|Germany|Baden-Wuerttemberg|BOLD:ABV4512
Physetopoda daghestanica|BC ZSM HYM 10834|M|Austria|Styria
Physetopoda daghestanica|BC ZSM HYM 10569|M|Hungary|Pest
Physetopoda daghestanica|BC ZSM HYM 10568|M|Hungary|Pest
Physetopoda scutellaris|BC ZSM HYM 10624|M|Germany|Bavaria
Physetopoda scutellaris|BC ZSM HYM 10607|M|Germany|Bavaria
Physetopoda scutellaris|BC ZSM HYM 10605|M|Germany|Bavaria
Physetopoda scutellaris|BC ZSM HYM 06639|M|Germany|Bavaria
Physetopoda scutellaris|BC ZSM HYM 06612|M|Germany|Bavaria
Physetopoda scutellaris|BC ZSM HYM 06600|M|Germany|Bavaria
Physetopoda scutellaris|BC ZSM HYM 06588|M|Germany|Bavaria
Smicromyrme rufipes|BC ZSM HYM 22670|M|Germany|Brandenburg|BOLD:AAU3641
Smicromyrme rufipes|BC ZSM HYM 10594|F|Germany|Berlin|BOLD:AAU3641
Smicromyrme rufipes|BC ZSM HYM 10552|F|Germany|Brandenburg|BOLD:AAU3641
Smicromyrme rufipes|BC ZSM HYM 10600|M|Germany|Brandenburg|BOLD:AAU3641
Smicromyrme rufipes|BC ZSM HYM 10581|M|Germany|Brandenburg|BOLD:AAU3641
Smicromyrme rufipes|BC ZSM HYM 10548|M|Germany|Berlin|BOLD:AAU3641
Smicromyrme rufipes|BC ZSM HYM 10579|M|Germany|Saxony-Anhalt|BOLD:AAU3641
Smicromyrme rufipes|BC ZSM HYM 10595|F|Germany|Brandenburg|BOLD:AAU3641
Smicromyrme rufipes|BC ZSM HYM 10596|F|Germany|Brandenburg|BOLD:AAU3641
Smicromyrme rufipes|BC ZSM HYM 10599|M|Germany|Brandenburg|BOLD:AAU3641
Smicromyrme rufipes|BC ZSM HYM 10551|F|Germany|Brandenburg|BOLD:AAU3641
Smicromyrme rufipes|BC ZSM HYM 22649|M|Germany|Brandenburg|BOLD:AAU3641
Smicromyrme rufipes|BC ZSM HYM 10597|M|Germany|Brandenburg|BOLD:AAU3641
Smicromyrme rufipes|BC ZSM HYM 10550|F|Germany|Brandenburg|BOLD:AAU3641
Smicromyrme rufipes|BC ZSM HYM 10549|M|Germany|Berlin|BOLD:AAU3641
Smicromyrme rufipes|BC ZSM HYM 10547|M|Germany|Brandenburg|BOLD:AAU3641
Smicromyrme rufipes|BC ZSM HYM 10546|M|Germany|Brandenburg|BOLD:AAU3641
Smicromyrme rufipes|BC ZSM HYM 09855|M|Germany|Brandenburg|BOLD:AAU3641
Smicromyrme rufipes|BC ZSM HYM 06624|M|Germany|Berlin|BOLD:AAU3641
Smicromyrme rufipes|BC ZSM HYM 06565|M|Germany|Berlin|BOLD:AAU3641
Smicromyrme rufipes|BC ZSM HYM 22662|M|Germany|Brandenburg|BOLD:AAU3641
Smicromyrme lombardensis|BC ZSM HYM 17467|F|Italy|Lombardy|BOLD:ABU8217
Smicromyrme lombardensis|BC ZSM HYM 10620|F|Italy|Lombardy|BOLD:ABU8217
Smicromyrme lombardensis|BC ZSM HYM 10618|M|Italy|Lombardy|BOLD:ABU8217
Smicromyrme burgeri|BC ZSM HYM 10617|M|France|Provence-Alpes-Cote d'Azur|BOLD:AAU3642
Smicromyrme burgeri|BC ZSM HYM 06577|M|Germany|Bavaria|BOLD:AAU3642
Smicromyrme burgeri|BC ZSM HYM 08196|F|Germany|Rhineland-Palatinate|BOLD:AAU3642
Smicromyrme burgeri|BC ZSM HYM 08195|F|Germany|Rhineland-Palatinate|BOLD:AAU3642
Smicromyrme burgeri|BC ZSM HYM 10590|F|Germany|Bavaria|BOLD:AAU3642
Smicromyrme burgeri|BC ZSM HYM 10626|M|Germany|Bavaria|BOLD:AAU3642
Smicromyrme burgeri|BC ZSM HYM 10593|F|Germany|Bavaria|BOLD:AAU3642
Smicromyrme burgeri|BC ZSM HYM 10591|F|Germany|Bavaria|BOLD:AAU3642
Smicromyrme burgeri|BC ZSM HYM 10592|F|Germany|Bavaria|BOLD:AAU3642
Smicromyrme burgeri|BC ZSM HYM 08149|M|Germany|Hesse|BOLD:AAU3642
Smicromyrme burgeri|BC ZSM HYM 08148|M|Germany|Rhineland-Palatinate|BOLD:AAU3642
Eumenes coarctatus|BC ZSM HYM 15254|M|Germany|Brandenburg|BOLD:AAN3538
Eumenes coarctatus|BC ZSM HYM 15253|M|Germany|Brandenburg|BOLD:AAN3538
Eumenes coarctatus|BC ZSM HYM 04968|F|Germany|Brandenburg|BOLD:AAN3538
Eumenes coarctatus|BC ZSM HYM 15252|F|Germany|Brandenburg|BOLD:AAN3538
Eumenes coarctatus|BC ZSM HYM 04970|M|Germany|Brandenburg|BOLD:AAN3538
Eumenes coarctatus|BC ZSM HYM 04971|M|Germany|Brandenburg|BOLD:AAN3538
Eumenes coarctatus|BC ZSM HYM 17372|M|Germany|Baden-Wuerttemberg|BOLD:AAN3538
Eumenes coarctatus|BC ZSM HYM 09887|F|Germany|Brandenburg|BOLD:AAN3538
Eumenes coarctatus|BC ZSM HYM 04969|F|Germany|Brandenburg|BOLD:AAN3538
Eumenes coarctatus|BC ZSM HYM 07818|F|Germany|Bavaria|BOLD:AAN3538
Eumenes coarctatus|BC ZSM HYM 04994|M|France|Provence-Alpes-Cote d'Azur|BOLD:ACE4606
Eumenes coarctatus|BC ZSM HYM 17363|M|Italy|Aosta Valley|BOLD:ACE4606
Eumenes coarctatus|BC ZSM HYM 17362|M|Italy|Aosta Valley|BOLD:ACE4606
Eumenes coarctatus|BC ZSM HYM 17365|F|Italy|Aosta Valley|BOLD:ACE4606
Eumenes coarctatus|BC ZSM HYM 04995|M|France|Provence-Alpes-Cote d'Azur|BOLD:ACE4606
Eumenes coarctatus|BC ZSM HYM 04986|M|Italy|Lombardy|BOLD:ACE4606
Eumenes coarctatus|BC ZSM HYM 10213|M|Italy|Veneto|BOLD:ACE4606
Eumenes coarctatus|BC-ZSM-HYM-29771-G07||Italy|Veneto|BOLD:ACE4606
Eumenes coarctatus|BC-ZSM-HYM-29771-G06||Italy|Veneto|BOLD:ACE4606
Eumenes coarctatus lunulatus|BC ZSM HYM 21143|M|Cyprus||BOLD:AAN4043
Eumenes coarctatus lunulatus|BC ZSM HYM 21142|M|Cyprus||BOLD:AAN4043
Eumenes coarctatus lunulatus|BC ZSM HYM 21141|F|Cyprus||BOLD:AAN4043
Eumenes coarctatus lunulatus|BC ZSM HYM 21140|F|Cyprus||BOLD:AAN4043
Eumenes mediterraneus|BC ZSM HYM 21147|F|Cyprus||BOLD:ACR5439
Eumenes mediterraneus|BC ZSM HYM 21146|M|Cyprus||BOLD:ACR5439
Eumenes pomiformis|BC ZSM HYM 23636|F|Kyrgyzstan||BOLD:ACV9324
Eumenes pomiformis|BC ZSM HYM 04982|M|Italy|Trentino-Alto Adige|BOLD:AAN4044
Eumenes pomiformis|BC ZSM HYM 10214|M|Italy|Veneto|BOLD:AAN4044
Eumenes pomiformis|BC ZSM HYM 17479|M|Italy|Aosta Valley|BOLD:AAN4044
Eumenes pomiformis|BC ZSM HYM 04985|M|Italy|Veneto|BOLD:AAN4044
Eumenes pomiformis|BC ZSM HYM 04984|M|Italy|Veneto|BOLD:AAN4044
Eumenes pomiformis|BC ZSM HYM 04983|M|Italy|Aosta Valley|BOLD:AAN4044
Eumenes pomiformis|BC ZSM HYM 10212|M|Italy|Veneto|BOLD:AAN4044
Eumenes pomiformis|BC ZSM HYM 10210|M|Italy|Veneto|BOLD:AAN4044

Eumenes pomiformis|BC ZSM HYM 04983|M|Italy|Aosta Valley|BOLD:AAN4044
Eumenes pomiformis|BC ZSM HYM 10212|M|Italy|Veneto|BOLD:AAN4044
Eumenes pomiformis|BC ZSM HYM 10210|M|Italy|Veneto|BOLD:AAN4044
Eumenes subpomiformis|BC-ZSM-HYM-29771-G05||France||BOLD:ACG1686
Eumenes pedunculatus|BC ZSM HYM 00045|M|Germany|Bavaria|BOLD:AAL4355
Eumenes pedunculatus|BC ZSM HYM 17370|M|Germany|Baden-Wuerttemberg|BOLD:AAL4355
Eumenes pedunculatus|BC ZSM HYM 04978|F|Germany|Baden-Wuerttemberg|BOLD:AAL4355
Eumenes pedunculatus|BC ZSM HYM 04979|F|Germany|Baden-Wuerttemberg|BOLD:AAL4355
Eumenes pedunculatus|BC ZSM HYM 04980|F|Germany|Bavaria|BOLD:AAL4355
Eumenes pedunculatus|BC ZSM HYM 15255|M|Germany|Brandenburg|BOLD:AAL4355
Eumenes pedunculatus|BC ZSM HYM 15256|F|Germany|Saxony-Anhalt|BOLD:AAL4355
Eumenes pedunculatus|BC ZSM HYM 17368|M|Germany|Baden-Wuerttemberg|BOLD:AAL4355
Eumenes pedunculatus|BC ZSM HYM 17369|M|Germany|Baden-Wuerttemberg|BOLD:AAL4355
Eumenes pedunculatus|BC ZSM HYM 17371|M|Germany|Baden-Wuerttemberg|BOLD:AAL4355
Eumenes pedunculatus|BC ZSM HYM 13990|F|Germany|Brandenburg|BOLD:AAL4355
Eumenes pedunculatus|BC ZSM HYM 04977|F|Germany|Baden-Wuerttemberg|BOLD:AAL4355
Eumenes pedunculatus|BC ZSM HYM 04976|F|Germany|Bavaria|BOLD:AAL4355
Eumenes pedunculatus|BC ZSM HYM 09883|M|Germany|Brandenburg|BOLD:AAL4355
Eumenes pedunculatus|BC ZSM HYM 10215|M|Germany|Baden-Wuerttemberg|BOLD:AAL4355
Eumenes pedunculatus|BC ZSM HYM 08263|M|Germany|Baden-Wuerttemberg|BOLD:AAL4355
Eumenes coronatus|BC ZSM HYM 13005|F|Germany|Thuringia|BOLD:AAN3639
Eumenes coronatus|BC ZSM HYM 17366|F|Germany|Baden-Wuerttemberg|BOLD:AAN3639
Eumenes coronatus|BC ZSM HYM 13092|M|Germany|Thuringia|BOLD:AAN3639
Eumenes coronatus|BC ZSM HYM 17367|F|Germany|Baden-Wuerttemberg|BOLD:AAN3639
Eumenes coronatus|BC ZSM HYM 08206|M|Germany|Rhineland-Palatinate|BOLD:AAN3639
Eumenes coronatus|BC ZSM HYM 08205|F|Germany|Rhineland-Palatinate|BOLD:AAN3639
Eumenes coronatus|BC ZSM HYM 08204|F|Germany|Rhineland-Palatinate|BOLD:AAN3639
Eumenes coronatus|BC ZSM HYM 04972|M|Germany|Berlin|BOLD:AAN3639
Eumenes coronatus|BC ZSM HYM 13004|F|Germany|Thuringia|BOLD:AAN3639
Eumenes coronatus|BC ZSM HYM 13003|M|Germany|Thuringia|BOLD:AAN3639
Eumenes coronatus|BC ZSM HYM 13002|M|Germany|Thuringia|BOLD:AAN3639
Eumenes coronatus|BC ZSM HYM 04973|M|Germany|Brandenburg|BOLD:AAN3639
Eumenes coronatus|BC ZSM HYM 04974|M|Germany|Brandenburg|BOLD:AAN3639
Eumenes coronatus|BC ZSM HYM 04975|F|Germany|Bavaria|BOLD:AAN3639
Eumenes coronatus|BC ZSM HYM 08207|M|Germany|Rhineland-Palatinate|BOLD:AAN3639
Eumenes coronatus|BC ZSM HYM 15263|F|Germany|Saxony-Anhalt|BOLD:AAN3639
Eumenes coronatus|BC ZSM HYM 07819|F|Germany|Bavaria|BOLD:AAN3639
Eumenes papillarius|BC ZSM HYM 08216|F|Germany|Rhineland-Palatinate|BOLD:AAY8811
Eumenes papillarius|BC ZSM HYM 08217|F|Germany|Rhineland-Palatinate|BOLD:AAY8811
Eumenes papillarius|BC ZSM HYM 13093|M|Germany|Thuringia|BOLD:AAY8811
Eumenes papillarius|BC ZSM HYM 04981|M|Italy|Trentino-Alto Adige|BOLD:AAY8811
Eumenes papillarius|BC ZSM HYM 13095|F|Germany|Thuringia|BOLD:AAY8811
Eumenes papillarius|BC ZSM HYM 13094|M|Germany|Thuringia|BOLD:AAY8811
Eumenes papillarius|BC ZSM HYM 09886|M|Germany|Brandenburg|BOLD:AAY8811
Eumenes papillarius|BC ZSM HYM 09885|M|Germany|Brandenburg|BOLD:AAY8811
Eumenes papillarius|BC ZSM HYM 09884|M|Germany|Brandenburg|BOLD:AAY8811
Eumenes papillarius|BC ZSM HYM 08219|M|Germany|Rhineland-Palatinate|BOLD:AAY8811
Eumenes papillarius|BC ZSM HYM 08218|M|Germany|Rhineland-Palatinate|BOLD:AAY8811
Eumenes dubius|BC ZSM HYM 21145|F|Cyprus||BOLD:ACR5018
Eumenes dubius|BC ZSM HYM 21144|F|Cyprus||BOLD:ACR5018
Eumenes dubius|BC ZSM HYM 24031|F|Spain|Andalusia|BOLD:ACV9421
Eumenes dubius|BC ZSM HYM 24029|F|Spain||BOLD:ACV9421
Eumenes sareptanus|BC ZSM HYM 13127|M|Hungary||BOLD:ABA9380
Eumenes sareptanus|BC ZSM HYM 04996|M|Germany|Baden-Wuerttemberg|BOLD:ABA9380
Eumenes sareptanus|BC ZSM HYM 13128|F|Hungary||BOLD:ABA9380
Eumenes sareptanus|BC ZSM HYM 12909|F|Hungary||BOLD:ABA9380
Eumenes sareptanus|BC ZSM HYM 13057|M|Germany|Brandenburg|BOLD:ABA9380
Eumenes sareptanus|BC ZSM HYM 24028|F|Spain||BOLD:ABA9380
Eumenes sareptanus|BC ZSM HYM 13056|F|Germany|Brandenburg|BOLD:ABA9380
Eumenes sareptanus|BC ZSM HYM 13055|F|Germany|Brandenburg|BOLD:ABA9380
Eumenes sareptanus|BC ZSM HYM 10211|M|Italy|Veneto|BOLD:ABA9380
Delta unguiculatum|BC ZSM HYM 04803|F|Italy|Lombardy|BOLD:AAN1374
Katamenes arbustorum|BC ZSM HYM 04804|M|Italy|Lombardy|BOLD:AAM6807
Katamenes arbustorum|BC ZSM HYM 04805|F|Italy|Veneto|BOLD:AAM6807
Katamenes arbustorum|BC ZSM HYM 06630|M|France|Provence-Alpes-Cote d'Azur|BOLD:AAM6807
Katamenes arbustorum|BC ZSM HYM 06618|M|France|Provence-Alpes-Cote d'Azur|BOLD:AAM6807
Euodynerus disconotatus laniensis|GBOL18843|M|Cyprus||BOLD:ADD0366
Euodynerus disconotatus laniensis|GBOL18842|M|Cyprus||BOLD:ADD0366
Euodynerus disconotatus laniensis|GBOL18841|M|Cyprus||BOLD:ADD0366
Stenodynerus clypeopictus|BC-ZSM-HYM-23654-C07||Slovakia||BOLD:ADH6201
Stenodynerus chevrieranus|BC ZSM HYM 04835|M|Switzerland|Valais|BOLD:AAN0040
Stenodynerus chevrieranus|BC ZSM HYM 24663||Germany|Thuringia|BOLD:AAN0040
Stenodynerus chevrieranus|BC-ZSM-HYM-23654-G01||Slovakia||BOLD:AAN0040
Stenodynerus xanthomelas|BC ZSM HYM 04843|F|Germany|Bavaria|BOLD:AAM3836
Stenodynerus xanthomelas|BC ZSM HYM 04786|F|Germany|Bavaria|BOLD:AAM3836
Stenodynerus xanthomelas|BC ZSM HYM 17382|M|Germany|Baden-Wuerttemberg|BOLD:AAM3836
Stenodynerus xanthomelas|BC ZSM HYM 17380|F|Germany|Baden-Wuerttemberg|BOLD:AAM3836
Stenodynerus xanthomelas|BC ZSM HYM 17381|F|Germany|Baden-Wuerttemberg|BOLD:AAM3836
Stenodynerus xanthomelas|BC ZSM HYM 11897|F|Germany|Bavaria|BOLD:AAM3836
Stenodynerus xanthomelas|BC ZSM HYM 10221|F|Germany|Baden-Wuerttemberg|BOLD:AAM3836
Stenodynerus xanthomelas|BC ZSM HYM 07409|F|Germany|Bavaria|BOLD:AAM3836
Stenodynerus punctifrons|BC ZSM HYM 06619|M|France|Provence-Alpes-Cote d'Azur|BOLD:AAU2628
Stenodynerus punctifrons|BC ZSM HYM 06643|M|France|Provence-Alpes-Cote d'Azur|BOLD:AAU2628
Stenodynerus punctifrons|BC ZSM HYM 06631|M|France|Provence-Alpes-Cote d'Azur|BOLD:AAU2628
Stenodynerus punctifrons|BC ZSM HYM 06607|M|France|Provence-Alpes-Cote d'Azur|BOLD:AAU2628
Stenodynerus picticrus|BC-ZSM-HYM-27676-D06||Germany|Bavaria|BOLD:ADG3096
Stenodynerus steckianus|BC ZSM HYM 04842|M|Italy|Trentino-Alto Adige|BOLD:AAM4331
Stenodynerus steckianus|BC ZSM HYM 04840|F|Italy|Veneto|BOLD:AAM4331
Stenodynerus jurinei|BC-ZSM-HYM-27676-D05||Germany|Bavaria|BOLD:AAM3906
Stenodynerus jurinei|BC ZSM HYM 04838|F|Italy|Lombardy|BOLD:AAM3906
Stenodynerus jurinei|BC ZSM HYM 04839|F|Italy|Lombardy|BOLD:AAM3906
Stenodynerus jurinei|BC ZSM HYM 04836|F|Italy|Lombardy|BOLD:AAM3906
Stenodynerus jurinei|BC ZSM HYM 04837|M|Italy|Lombardy|BOLD:AAM3906
Stenodynerus bluethgeni|BC ZSM HYM 04831|M|France|Provence-Alpes-Cote d'Azur|BOLD:AAM4330
Stenodynerus bluethgeni|BC ZSM HYM 04834|F|Italy|Aosta Valley|BOLD:AAM4330
Stenodynerus bluethgeni|BC ZSM HYM 04832|F|Italy|Aosta Valley|BOLD:AAM4330
Stenodynerus bluethgeni|BC ZSM HYM 04841|F|Italy|Lombardy|BOLD:AAM4330
Stenodynerus bluethgeni|BC ZSM HYM 17385|F|Germany|Baden-Wuerttemberg|BOLD:AAM4330
Stenodynerus bluethgeni|BC ZSM HYM 10219|F|Germany|Baden-Wuerttemberg|BOLD:AAM4330
[illegible]

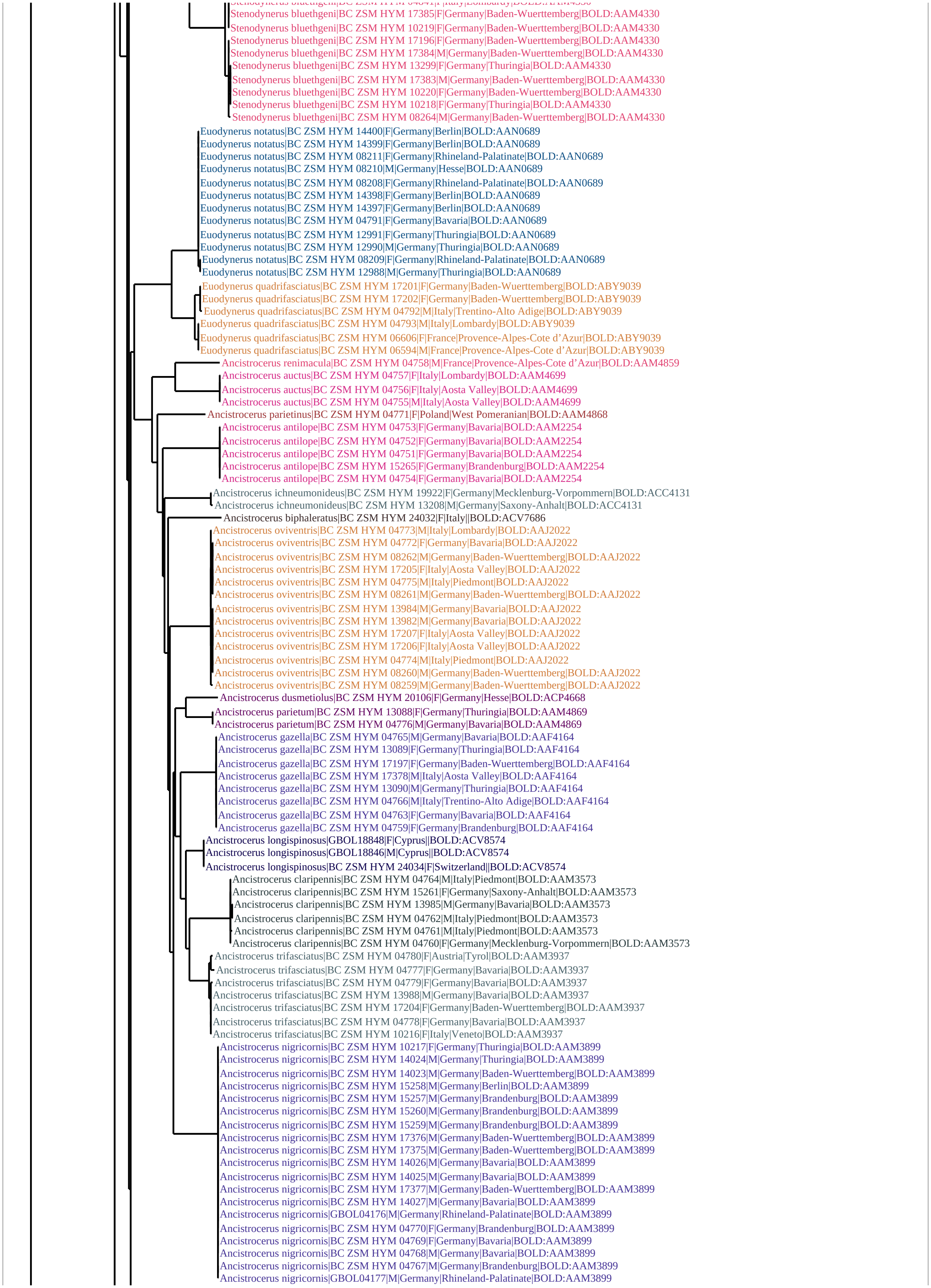
Stenodynerus bluethgeni|BC ZSM HYM 04841|F|Italy|Lombardy|BOLD:AAM4330
Stenodynerus bluethgeni|BC ZSM HYM 17385|F|Germany|Baden-Wuerttemberg|BOLD:AAM4330
Stenodynerus bluethgeni|BC ZSM HYM 10219|F|Germany|Baden-Wuerttemberg|BOLD:AAM4330
Stenodynerus bluethgeni|BC ZSM HYM 17196|F|Germany|Baden-Wuerttemberg|BOLD:AAM4330
Stenodynerus bluethgeni|BC ZSM HYM 17384|M|Germany|Baden-Wuerttemberg|BOLD:AAM4330
Stenodynerus bluethgeni|BC ZSM HYM 13299|F|Germany|Thuringia|BOLD:AAM4330
Stenodynerus bluethgeni|BC ZSM HYM 17383|M|Germany|Baden-Wuerttemberg|BOLD:AAM4330
Stenodynerus bluethgeni|BC ZSM HYM 10220|F|Germany|Baden-Wuerttemberg|BOLD:AAM4330
Stenodynerus bluethgeni|BC ZSM HYM 10218|F|Germany|Thuringia|BOLD:AAM4330
Stenodynerus bluethgeni|BC ZSM HYM 08264|M|Germany|Baden-Wuerttemberg|BOLD:AAM4330
Euodynerus notatus|BC ZSM HYM 14400|F|Germany|Berlin|BOLD:AAN0689
Euodynerus notatus|BC ZSM HYM 14399|F|Germany|Berlin|BOLD:AAN0689
Euodynerus notatus|BC ZSM HYM 08211|F|Germany|Rhineland-Palatinate|BOLD:AAN0689
Euodynerus notatus|BC ZSM HYM 08210|M|Germany|Hesse|BOLD:AAN0689
Euodynerus notatus|BC ZSM HYM 08208|F|Germany|Rhineland-Palatinate|BOLD:AAN0689
Euodynerus notatus|BC ZSM HYM 14398|F|Germany|Berlin|BOLD:AAN0689
Euodynerus notatus|BC ZSM HYM 14397|F|Germany|Berlin|BOLD:AAN0689
Euodynerus notatus|BC ZSM HYM 04791|F|Germany|Bavaria|BOLD:AAN0689
Euodynerus notatus|BC ZSM HYM 12991|F|Germany|Thuringia|BOLD:AAN0689
Euodynerus notatus|BC ZSM HYM 12990|M|Germany|Thuringia|BOLD:AAN0689
Euodynerus notatus|BC ZSM HYM 08209|F|Germany|Rhineland-Palatinate|BOLD:AAN0689
Euodynerus notatus|BC ZSM HYM 12988|M|Germany|Thuringia|BOLD:AAN0689
Euodynerus quadrifasciatus|BC ZSM HYM 17201|F|Germany|Baden-Wuerttemberg|BOLD:ABY9039
Euodynerus quadrifasciatus|BC ZSM HYM 17202|F|Germany|Baden-Wuerttemberg|BOLD:ABY9039
Euodynerus quadrifasciatus|BC ZSM HYM 04792|M|Italy|Trentino-Alto Adige|BOLD:ABY9039
Euodynerus quadrifasciatus|BC ZSM HYM 04793|M|Italy|Lombardy|BOLD:ABY9039
Euodynerus quadrifasciatus|BC ZSM HYM 06606|F|France|Provence-Alpes-Cote d'Azur|BOLD:ABY9039
Euodynerus quadrifasciatus|BC ZSM HYM 06594|M|France|Provence-Alpes-Cote d'Azur|BOLD:ABY9039
Ancistrocerus renimacula|BC ZSM HYM 04758|M|France|Provence-Alpes-Cote d'Azur|BOLD:AAM4859
Ancistrocerus auctus|BC ZSM HYM 04757|F|Italy|Lombardy|BOLD:AAM4699
Ancistrocerus auctus|BC ZSM HYM 04756|F|Italy|Aosta Valley|BOLD:AAM4699
Ancistrocerus auctus|BC ZSM HYM 04755|M|Italy|Aosta Valley|BOLD:AAM4699
Ancistrocerus parietinus|BC ZSM HYM 04771|F|Poland|West Pomeranian|BOLD:AAM4868
Ancistrocerus antilope|BC ZSM HYM 04753|F|Germany|Bavaria|BOLD:AAM2254
Ancistrocerus antilope|BC ZSM HYM 04752|F|Germany|Bavaria|BOLD:AAM2254
Ancistrocerus antilope|BC ZSM HYM 04751|F|Germany|Bavaria|BOLD:AAM2254
Ancistrocerus antilope|BC ZSM HYM 15265|F|Germany|Brandenburg|BOLD:AAM2254
Ancistrocerus antilope|BC ZSM HYM 04754|F|Germany|Bavaria|BOLD:AAM2254
Ancistrocerus ichneumonideus|BC ZSM HYM 19922|F|Germany|Mecklenburg-Vorpommern|BOLD:ACC4131
Ancistrocerus ichneumonideus|BC ZSM HYM 13208|M|Germany|Saxony-Anhalt|BOLD:ACC4131
Ancistrocerus biphaleratus|BC ZSM HYM 24032|F|Italy||BOLD:ACV7686
Ancistrocerus oviventris|BC ZSM HYM 04773|M|Italy|Lombardy|BOLD:AAJ2022
Ancistrocerus oviventris|BC ZSM HYM 04772|F|Germany|Bavaria|BOLD:AAJ2022
Ancistrocerus oviventris|BC ZSM HYM 08262|M|Germany|Baden-Wuerttemberg|BOLD:AAJ2022
Ancistrocerus oviventris|BC ZSM HYM 17205|F|Italy|Aosta Valley|BOLD:AAJ2022
Ancistrocerus oviventris|BC ZSM HYM 04775|M|Italy|Piedmont|BOLD:AAJ2022
Ancistrocerus oviventris|BC ZSM HYM 08261|M|Germany|Baden-Wuerttemberg|BOLD:AAJ2022
Ancistrocerus oviventris|BC ZSM HYM 13984|M|Germany|Bavaria|BOLD:AAJ2022
Ancistrocerus oviventris|BC ZSM HYM 13982|M|Germany|Bavaria|BOLD:AAJ2022
Ancistrocerus oviventris|BC ZSM HYM 17207|F|Italy|Aosta Valley|BOLD:AAJ2022
Ancistrocerus oviventris|BC ZSM HYM 17206|F|Italy|Aosta Valley|BOLD:AAJ2022
Ancistrocerus oviventris|BC ZSM HYM 04774|M|Italy|Piedmont|BOLD:AAJ2022
Ancistrocerus oviventris|BC ZSM HYM 08260|M|Germany|Baden-Wuerttemberg|BOLD:AAJ2022
Ancistrocerus oviventris|BC ZSM HYM 08259|M|Germany|Baden-Wuerttemberg|BOLD:AAJ2022
Ancistrocerus dusmetiolus|BC ZSM HYM 20106|F|Germany|Hesse|BOLD:ACP4668
Ancistrocerus parietum|BC ZSM HYM 13088|F|Germany|Thuringia|BOLD:AAM4869
Ancistrocerus parietum|BC ZSM HYM 04776|M|Germany|Bavaria|BOLD:AAM4869
Ancistrocerus gazella|BC ZSM HYM 04765|M|Germany|Bavaria|BOLD:AAF4164
Ancistrocerus gazella|BC ZSM HYM 13089|F|Germany|Thuringia|BOLD:AAF4164
Ancistrocerus gazella|BC ZSM HYM 17197|F|Germany|Baden-Wuerttemberg|BOLD:AAF4164
Ancistrocerus gazella|BC ZSM HYM 17378|M|Italy|Aosta Valley|BOLD:AAF4164
Ancistrocerus gazella|BC ZSM HYM 13090|M|Germany|Thuringia|BOLD:AAF4164
Ancistrocerus gazella|BC ZSM HYM 04766|M|Italy|Trentino-Alto Adige|BOLD:AAF4164
Ancistrocerus gazella|BC ZSM HYM 04763|F|Germany|Bavaria|BOLD:AAF4164
Ancistrocerus gazella|BC ZSM HYM 04759|F|Germany|Brandenburg|BOLD:AAF4164
Ancistrocerus longispinosus|GBOL18848|F|Cyprus||BOLD:ACV8574
Ancistrocerus longispinosus|GBOL18846|M|Cyprus||BOLD:ACV8574
Ancistrocerus longispinosus|BC ZSM HYM 24034|F|Switzerland||BOLD:ACV8574
Ancistrocerus claripennis|BC ZSM HYM 04764|M|Italy|Piedmont|BOLD:AAM3573
Ancistrocerus claripennis|BC ZSM HYM 15261|F|Germany|Saxony-Anhalt|BOLD:AAM3573
Ancistrocerus claripennis|BC ZSM HYM 13985|M|Germany|Bavaria|BOLD:AAM3573
Ancistrocerus claripennis|BC ZSM HYM 04762|M|Italy|Piedmont|BOLD:AAM3573
Ancistrocerus claripennis|BC ZSM HYM 04761|M|Italy|Piedmont|BOLD:AAM3573
Ancistrocerus claripennis|BC ZSM HYM 04760|F|Germany|Mecklenburg-Vorpommern|BOLD:AAM3573
Ancistrocerus trifasciatus|BC ZSM HYM 04780|F|Austria|Tyrol|BOLD:AAM3937
Ancistrocerus trifasciatus|BC ZSM HYM 04777|F|Germany|Bavaria|BOLD:AAM3937
Ancistrocerus trifasciatus|BC ZSM HYM 04779|F|Germany|Bavaria|BOLD:AAM3937
Ancistrocerus trifasciatus|BC ZSM HYM 13988|M|Germany|Bavaria|BOLD:AAM3937
Ancistrocerus trifasciatus|BC ZSM HYM 17204|F|Germany|Baden-Wuerttemberg|BOLD:AAM3937
Ancistrocerus trifasciatus|BC ZSM HYM 04778|F|Germany|Bavaria|BOLD:AAM3937
Ancistrocerus trifasciatus|BC ZSM HYM 10216|F|Italy|Veneto|BOLD:AAM3937
Ancistrocerus nigricornis|BC ZSM HYM 10217|F|Germany|Thuringia|BOLD:AAM3899
Ancistrocerus nigricornis|BC ZSM HYM 14024|M|Germany|Thuringia|BOLD:AAM3899
Ancistrocerus nigricornis|BC ZSM HYM 14023|M|Germany|Baden-Wuerttemberg|BOLD:AAM3899
Ancistrocerus nigricornis|BC ZSM HYM 15258|M|Germany|Berlin|BOLD:AAM3899
Ancistrocerus nigricornis|BC ZSM HYM 15257|M|Germany|Brandenburg|BOLD:AAM3899
Ancistrocerus nigricornis|BC ZSM HYM 15260|M|Germany|Brandenburg|BOLD:AAM3899
Ancistrocerus nigricornis|BC ZSM HYM 15259|M|Germany|Brandenburg|BOLD:AAM3899
Ancistrocerus nigricornis|BC ZSM HYM 17376|M|Germany|Baden-Wuerttemberg|BOLD:AAM3899
Ancistrocerus nigricornis|BC ZSM HYM 17375|M|Germany|Baden-Wuerttemberg|BOLD:AAM3899
Ancistrocerus nigricornis|BC ZSM HYM 14026|M|Germany|Bavaria|BOLD:AAM3899
Ancistrocerus nigricornis|BC ZSM HYM 14025|M|Germany|Bavaria|BOLD:AAM3899
Ancistrocerus nigricornis|BC ZSM HYM 17377|M|Germany|Baden-Wuerttemberg|BOLD:AAM3899
Ancistrocerus nigricornis|BC ZSM HYM 14027|M|Germany|Bavaria|BOLD:AAM3899
Ancistrocerus nigricornis|GBOL04176|M|Germany|Rhineland-Palatinate|BOLD:AAM3899
Ancistrocerus nigricornis|BC ZSM HYM 04770|F|Germany|Brandenburg|BOLD:AAM3899
Ancistrocerus nigricornis|BC ZSM HYM 04769|F|Germany|Bavaria|BOLD:AAM3899
Ancistrocerus nigricornis|BC ZSM HYM 04768|M|Germany|Bavaria|BOLD:AAM3899
Ancistrocerus nigricornis|BC ZSM HYM 04767|M|Germany|Brandenburg|BOLD:AAM3899
Ancistrocerus nigricornis|GBOL04177|M|Germany|Rhineland-Palatinate|BOLD:AAM3899

Ancistrocerus nigricornis|BC ZSM HYM 04768|M|Germany|Bavaria|BOLD:AAM3899
Ancistrocerus nigricornis|BC ZSM HYM 04767|M|Germany|Brandenburg|BOLD:AAM3899
Ancistrocerus nigricornis|GBOL04177|M|Germany|Rhineland-Palatinate|BOLD:AAM3899
Antepipona deflenda|GBOL18868|F|Cyprus||BOLD:ADL0396
Antepipona deflenda|GBOL18867|M|Cyprus||BOLD:ADL0396
Allodynerus rossii|BC ZSM HYM 04785|F|Germany|Bavaria|BOLD:AAM3835
Allodynerus rossii|BC ZSM HYM 04787|F|Germany|Bavaria|BOLD:AAM3835
Allodynerus rossii|BC ZSM HYM 04784|F|Germany|Bavaria|BOLD:AAM3835
Allodynerus delphinalis|BC ZSM HYM 17198|M|Italy|Aosta Valley|BOLD:AAM4849
Allodynerus delphinalis|BC ZSM HYM 24664||Germany|Saxony-Anhalt|BOLD:AAM4849
Allodynerus delphinalis|BC ZSM HYM 04783|F|Germany|Baden-Wuerttemberg|BOLD:AAM4849
Allodynerus delphinalis|BC ZSM HYM 04782|F|Germany|Bavaria|BOLD:AAM4849
Antepipona orbitalis|BC ZSM HYM 13135|F|Hungary||BOLD:ACC2791
Antepipona orbitalis|BC ZSM HYM 13134|M|Hungary||BOLD:ACC2791
Antepipona orbitalis|BC ZSM HYM 13133|M|Hungary||BOLD:ACC2791
Antepipona orbitalis|BC-ZSM-HYM-23654-E01||Hungary|Budapest|BOLD:ACC2791
Euodynerus dantici|BC ZSM HYM 04789|F|France|Provence-Alpes-Cote d'Azur|BOLD:AAM5315
Euodynerus dantici|BC ZSM HYM 04788|M|France|Provence-Alpes-Cote d'Azur|BOLD:AAM5315
Symmorphus angustatus|BC ZSM HYM 13300|F|Germany|Saxony-Anhalt|BOLD:ACL6133
Symmorphus crassicornis|BC ZSM HYM 04954|F|Germany|Bavaria|BOLD:AAN3694
Symmorphus crassicornis|BC ZSM HYM 10635|F|Germany|Bavaria|BOLD:AAN3694
Symmorphus crassicornis|BC ZSM HYM 04953|F|Germany|Bavaria|BOLD:AAN3694
Symmorphus crassicornis|BC ZSM HYM 04952|F|Germany|Bavaria|BOLD:AAN3694
Symmorphus crassicornis|BC ZSM HYM 04951|F|Germany|Bavaria|BOLD:AAN3694
Symmorphus murarius|BC ZSM HYM 14405|F|Germany|Berlin|BOLD:AAN3692
Symmorphus murarius|BC ZSM HYM 14403|F|Germany|Berlin|BOLD:AAN3692
Symmorphus murarius|BC ZSM HYM 14402|F|Germany|Berlin|BOLD:AAN3692
Symmorphus murarius|BC ZSM HYM 04966|F|Germany|Bavaria|BOLD:AAN3692
Symmorphus murarius|BC ZSM HYM 14404|F|Germany|Berlin|BOLD:AAN3692
Symmorphus murarius|BC ZSM HYM 14401|F|Germany|Berlin|BOLD:AAN3692
Symmorphus murarius|BC ZSM HYM 04967|F|Germany|Bavaria|BOLD:AAN3692
Symmorphus murarius|BC ZSM HYM 04965|F|Germany|Bavaria|BOLD:AAN3692
Symmorphus murarius|BC ZSM HYM 04964|F|Germany|Bavaria|BOLD:AAN3692
Symmorphus debilitatus|BC ZSM HYM 17199|F|Germany|Baden-Wuerttemberg|BOLD:AAN3537
Symmorphus debilitatus|BC ZSM HYM 04958|F|Germany|Bavaria|BOLD:AAN3537
Symmorphus debilitatus|BC ZSM HYM 04957|F|Germany|Bavaria|BOLD:AAN3537
Symmorphus debilitatus|BC ZSM HYM 04956|F|Germany|Bavaria|BOLD:AAN3537
Symmorphus debilitatus|BC ZSM HYM 04955|F|Germany|Bavaria|BOLD:AAN3537
Symmorphus connexus|BC ZSM HYM 19926|F|Italy|South Tyrol|BOLD:AAN3693
Symmorphus connexus|BC ZSM HYM 04950|F|Germany|Bavaria|BOLD:AAN3693
Symmorphus connexus|BC ZSM HYM 04949|F|Germany|Bavaria|BOLD:AAN3693
Symmorphus connexus|BC ZSM HYM 04948|F|Germany|Bavaria|BOLD:AAN3693
Symmorphus bifasciatus|BC ZSM HYM 04944|F|Germany|Bavaria|BOLD:AAN3541
Symmorphus bifasciatus|BC ZSM HYM 04945|F|Germany|Bavaria|BOLD:AAN3541
Symmorphus bifasciatus|BC ZSM HYM 10634|F|Germany|Bavaria|BOLD:AAN3541
Symmorphus bifasciatus|BC ZSM HYM 10633|F|Germany|Bavaria|BOLD:AAN3541
Symmorphus bifasciatus|BC ZSM HYM 04947|F|Germany|Bavaria|BOLD:AAN3541
Symmorphus bifasciatus|BC ZSM HYM 10632|F|Germany|Bavaria|BOLD:AAN3541
Symmorphus allobrogus|BC ZSM HYM 04941|F|Germany|Bavaria|BOLD:AAN0135
Symmorphus allobrogus|BC ZSM HYM 04942|F|Germany|Bavaria|BOLD:AAN0135
Symmorphus allobrogus|BC ZSM HYM 13977|F|Italy|Lombardy|BOLD:AAN0135
Symmorphus allobrogus|BC ZSM HYM 04943|F|Germany|Bavaria|BOLD:AAN0135
Symmorphus allobrogus|BC ZSM HYM 04845|M|Italy|Lombardy|BOLD:AAN0135
Symmorphus gracilis|BC ZSM HYM 04960|F|Germany|Bavaria|BOLD:AAN3695
Symmorphus gracilis|BC ZSM HYM 04961|F|Germany|Bavaria|BOLD:AAN3695
Symmorphus gracilis|BC ZSM HYM 04963|M|Italy|Trentino-Alto Adige|BOLD:AAN3695
Symmorphus gracilis|BC ZSM HYM 13975|F|Germany|Bavaria|BOLD:AAN3695
Symmorphus gracilis|BC ZSM HYM 13974|F|Germany|Bavaria|BOLD:AAN3695
Symmorphus gracilis|BC ZSM HYM 04959|F|Germany|Bavaria|BOLD:AAN3695
Symmorphus gracilis|BC ZSM HYM 14702|M|Germany|Brandenburg|BOLD:AAN3695
Leptochilus tarsatus|BC ZSM HYM 04812|M|Italy|Piedmont|BOLD:AAM4311
Leptochilus tarsatus|BC ZSM HYM 04813|M|Italy|Piedmont|BOLD:AAM4311
Leptochilus tarsatus|BC ZSM HYM 04811|M|Italy|Piedmont|BOLD:AAM4311
Leptochilus moustirsensis|BC ZSM HYM 16244|F|France|Provence-Alpes-Cote d'Azur|BOLD:ACG1074
Leptochilus regulus|BC ZSM HYM 04809|M|France|Provence-Alpes-Cote d'Azur|BOLD:AAM9219
Leptochilus regulus|BC ZSM HYM 04810|M|France|Provence-Alpes-Cote d'Azur|BOLD:AAM9219
Leptochilus alpestris|BC ZSM HYM 04826|M|France|Provence-Alpes-Cote d'Azur|BOLD:AAM9218
Leptochilus alpestris|BC ZSM HYM 07397|F|France|Provence-Alpes-Cote d'Azur|BOLD:AAM9218
Leptochilus alpestris|BC ZSM HYM 04808|M|Italy|Piedmont|BOLD:AAM9217
Leptochilus alpestris|BC ZSM HYM 07378|F|France|Provence-Alpes-Cote d'Azur|BOLD:AAM9217
Microdynerus longicollis|BC ZSM HYM 04821|F|Germany|Bavaria|BOLD:AAN2278
Microdynerus nugdunensis|BC ZSM HYM 04818|F|Germany|Rhineland-Palatinate
Microdynerus timidus|BC ZSM HYM 08231|M|Germany|Rhineland-Palatinate|BOLD:AAY8818
Microdynerus timidus|BC ZSM HYM 08229|F|Germany|Rhineland-Palatinate|BOLD:AAY8818
Microdynerus timidus|BC ZSM HYM 13285|F|Germany|Saxony-Anhalt|BOLD:AAY8818
Microdynerus timidus|BC ZSM HYM 13284|F|Germany|Saxony-Anhalt|BOLD:AAY8818
Microdynerus timidus|BC ZSM HYM 08230|M|Germany|Rhineland-Palatinate|BOLD:AAY8818
Microdynerus timidus|BC ZSM HYM 08228|F|Germany|Rhineland-Palatinate|BOLD:AAY8818
Microdynerus parvulus|BC ZSM HYM 04825|M|Germany|Bavaria|BOLD:AAM3885
Microdynerus parvulus|BC ZSM HYM 04824|F|Germany|Brandenburg|BOLD:AAM3885
Microdynerus parvulus|BC ZSM HYM 04823|F|Germany|Bavaria|BOLD:AAM3885
Microdynerus parvulus|BC ZSM HYM 04822|F|Germany|Berlin|BOLD:AAM3885
Microdynerus parvulus|BC ZSM HYM 04817|F|Italy|Aosta Valley|BOLD:AAM3885
Microdynerus exilis|BC ZSM HYM 08227|M|Germany|Rhineland-Palatinate
Microdynerus exilis|BC ZSM HYM 08226|M|Germany|Rhineland-Palatinate
Microdynerus exilis|BC ZSM HYM 08225|F|Germany|Rhineland-Palatinate
Microdynerus exilis|BC ZSM HYM 08224|F|Germany|Rhineland-Palatinate
Pterocheilus phaleratus|BC ZSM HYM 14090|M|Germany|Brandenburg|BOLD:AAL2208
Pterocheilus phaleratus|BC ZSM HYM 14088|M|Germany|Brandenburg|BOLD:AAL2208
Pterocheilus phaleratus|BC ZSM HYM 00046|M|Germany|Brandenburg|BOLD:AAL2208
Pterocheilus phaleratus|BC ZSM HYM 14698|M|Germany|Brandenburg|BOLD:AAL2208
Pterocheilus phaleratus|BC ZSM HYM 14699|M|Germany|Brandenburg|BOLD:AAL2208
Pterocheilus phaleratus|BC ZSM HYM 19923|F|Germany|Mecklenburg-Vorpommern|BOLD:AAL2208
Pterocheilus phaleratus|BC ZSM HYM 14089|M|Germany|Brandenburg|BOLD:AAL2208
Pterocheilus phaleratus|BC ZSM HYM 21077|F|Germany|Mecklenburg-Vorpommern|BOLD:AAL2208
Pterocheilus phaleratus|BC ZSM HYM 04830|M|Germany|Brandenburg|BOLD:AAL2208
Pterocheilus phaleratus|BC ZSM HYM 04829|M|Germany|Brandenburg|BOLD:AAL2208
Pterocheilus phaleratus|BC ZSM HYM 04828|F|Germany|Brandenburg|BOLD:AAL2208
Pterocheilus phaleratus|BC ZSM HYM 04827|F|Germany|Brandenburg|BOLD:AAL2208
Pterocheilus phaleratus|BC ZSM HYM 14697|M|Germany|Brandenburg|BOLD:AAL2208

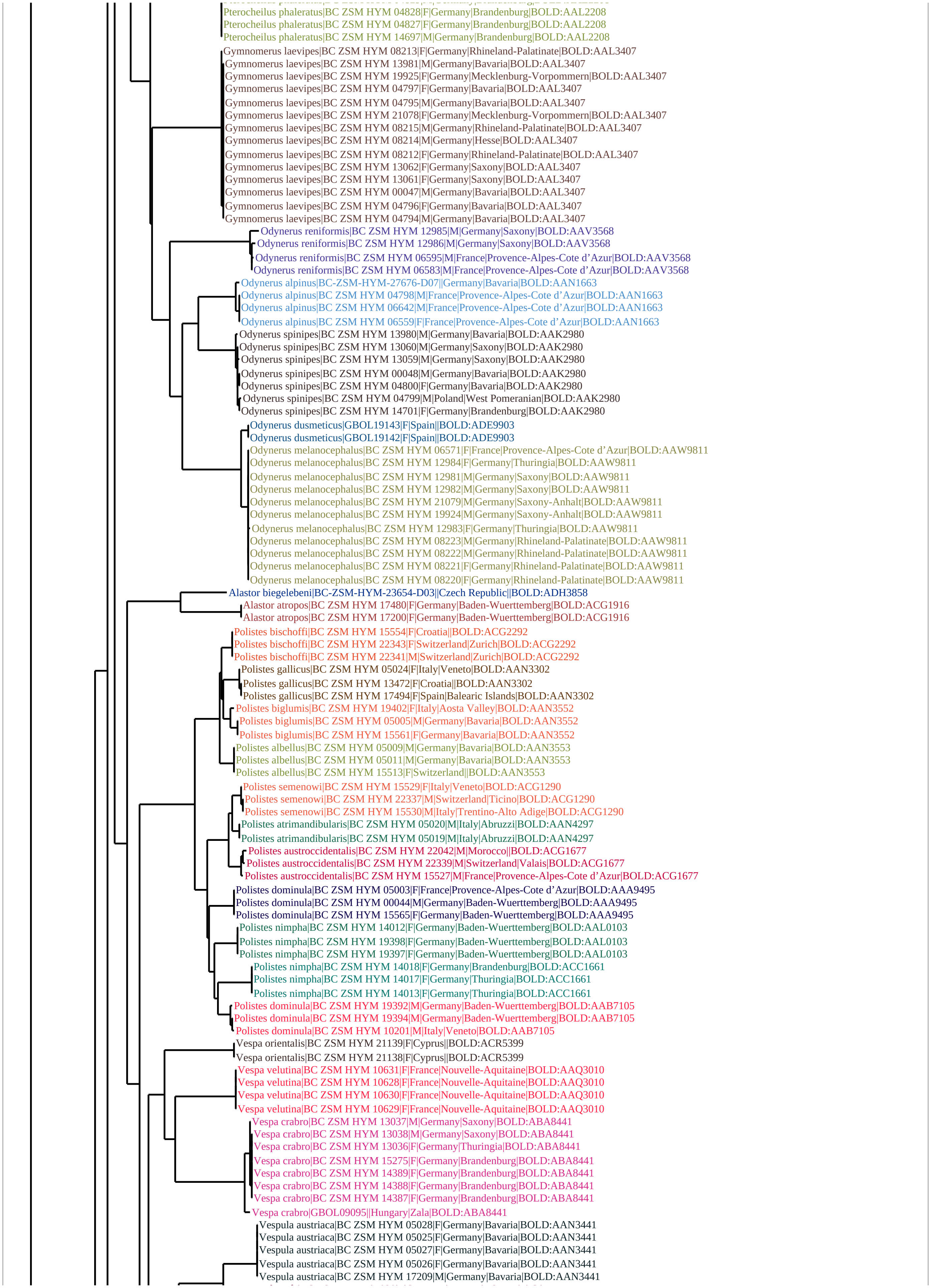
Pterocheilus phaleratus|BC ZSM HYM 04828|F|Germany|Brandenburg|BOLD:AAL2208
Pterocheilus phaleratus|BC ZSM HYM 04827|F|Germany|Brandenburg|BOLD:AAL2208
Pterocheilus phaleratus|BC ZSM HYM 14697|M|Germany|Brandenburg|BOLD:AAL2208
Gymnomerus laevipes|BC ZSM HYM 08213|F|Germany|Rhineland-Palatinate|BOLD:AAL3407
Gymnomerus laevipes|BC ZSM HYM 13981|M|Germany|Bavaria|BOLD:AAL3407
Gymnomerus laevipes|BC ZSM HYM 19925|F|Germany|Mecklenburg-Vorpommern|BOLD:AAL3407
Gymnomerus laevipes|BC ZSM HYM 04797|F|Germany|Bavaria|BOLD:AAL3407
Gymnomerus laevipes|BC ZSM HYM 04795|M|Germany|Bavaria|BOLD:AAL3407
Gymnomerus laevipes|BC ZSM HYM 21078|F|Germany|Mecklenburg-Vorpommern|BOLD:AAL3407
Gymnomerus laevipes|BC ZSM HYM 08215|M|Germany|Rhineland-Palatinate|BOLD:AAL3407
Gymnomerus laevipes|BC ZSM HYM 08214|M|Germany|Hesse|BOLD:AAL3407
Gymnomerus laevipes|BC ZSM HYM 08212|F|Germany|Rhineland-Palatinate|BOLD:AAL3407
Gymnomerus laevipes|BC ZSM HYM 13062|F|Germany|Saxony|BOLD:AAL3407
Gymnomerus laevipes|BC ZSM HYM 13061|F|Germany|Saxony|BOLD:AAL3407
Gymnomerus laevipes|BC ZSM HYM 00047|M|Germany|Bavaria|BOLD:AAL3407
Gymnomerus laevipes|BC ZSM HYM 04796|F|Germany|Bavaria|BOLD:AAL3407
Gymnomerus laevipes|BC ZSM HYM 04794|M|Germany|Bavaria|BOLD:AAL3407
Odynerus reniformis|BC ZSM HYM 12985|M|Germany|Saxony|BOLD:AAV3568
Odynerus reniformis|BC ZSM HYM 12986|M|Germany|Saxony|BOLD:AAV3568
Odynerus reniformis|BC ZSM HYM 06595|M|France|Provence-Alpes-Cote d'Azur|BOLD:AAV3568
Odynerus reniformis|BC ZSM HYM 06583|M|France|Provence-Alpes-Cote d'Azur|BOLD:AAV3568
Odynerus alpinus|BC-ZSM-HYM-27676-D07||Germany|Bavaria|BOLD:AAN1663
Odynerus alpinus|BC ZSM HYM 04798|M|France|Provence-Alpes-Cote d'Azur|BOLD:AAN1663
Odynerus alpinus|BC ZSM HYM 06642|M|France|Provence-Alpes-Cote d'Azur|BOLD:AAN1663
Odynerus alpinus|BC ZSM HYM 06559|F|France|Provence-Alpes-Cote d'Azur|BOLD:AAN1663
Odynerus spinipes|BC ZSM HYM 13980|M|Germany|Bavaria|BOLD:AAK2980
Odynerus spinipes|BC ZSM HYM 13060|M|Germany|Saxony|BOLD:AAK2980
Odynerus spinipes|BC ZSM HYM 13059|M|Germany|Saxony|BOLD:AAK2980
Odynerus spinipes|BC ZSM HYM 00048|M|Germany|Bavaria|BOLD:AAK2980
Odynerus spinipes|BC ZSM HYM 04800|F|Germany|Bavaria|BOLD:AAK2980
Odynerus spinipes|BC ZSM HYM 04799|M|Poland|West Pomeranian|BOLD:AAK2980
Odynerus spinipes|BC ZSM HYM 14701|F|Germany|Brandenburg|BOLD:AAK2980
Odynerus dusmeticus|GBOL19143|F|Spain||BOLD:ADE9903
Odynerus dusmeticus|GBOL19142|F|Spain||BOLD:ADE9903
Odynerus melanocephalus|BC ZSM HYM 06571|F|France|Provence-Alpes-Cote d'Azur|BOLD:AAW9811
Odynerus melanocephalus|BC ZSM HYM 12984|F|Germany|Thuringia|BOLD:AAW9811
Odynerus melanocephalus|BC ZSM HYM 12981|M|Germany|Saxony|BOLD:AAW9811
Odynerus melanocephalus|BC ZSM HYM 12982|M|Germany|Saxony|BOLD:AAW9811
Odynerus melanocephalus|BC ZSM HYM 21079|M|Germany|Saxony-Anhalt|BOLD:AAW9811
Odynerus melanocephalus|BC ZSM HYM 19924|M|Germany|Saxony-Anhalt|BOLD:AAW9811
Odynerus melanocephalus|BC ZSM HYM 12983|F|Germany|Thuringia|BOLD:AAW9811
Odynerus melanocephalus|BC ZSM HYM 08223|M|Germany|Rhineland-Palatinate|BOLD:AAW9811
Odynerus melanocephalus|BC ZSM HYM 08222|M|Germany|Rhineland-Palatinate|BOLD:AAW9811
Odynerus melanocephalus|BC ZSM HYM 08221|F|Germany|Rhineland-Palatinate|BOLD:AAW9811
Odynerus melanocephalus|BC ZSM HYM 08220|F|Germany|Rhineland-Palatinate|BOLD:AAW9811
Alastor biegelebeni|BC-ZSM-HYM-23654-D03||Czech Republic||BOLD:ADH3858
Alastor atropos|BC ZSM HYM 17480|F|Germany|Baden-Wuerttemberg|BOLD:ACG1916
Alastor atropos|BC ZSM HYM 17200|F|Germany|Baden-Wuerttemberg|BOLD:ACG1916
Polistes bischoffi|BC ZSM HYM 15554|F|Croatia||BOLD:ACG2292
Polistes bischoffi|BC ZSM HYM 22343|F|Switzerland|Zurich|BOLD:ACG2292
Polistes bischoffi|BC ZSM HYM 22341|M|Switzerland|Zurich|BOLD:ACG2292
Polistes gallicus|BC ZSM HYM 05024|F|Italy|Veneto|BOLD:AAN3302
Polistes gallicus|BC ZSM HYM 13472|F|Croatia||BOLD:AAN3302
Polistes gallicus|BC ZSM HYM 17494|F|Spain|Balearic Islands|BOLD:AAN3302
Polistes biglumis|BC ZSM HYM 19402|F|Italy|Aosta Valley|BOLD:AAN3552
Polistes biglumis|BC ZSM HYM 05005|M|Germany|Bavaria|BOLD:AAN3552
Polistes biglumis|BC ZSM HYM 15561|F|Germany|Bavaria|BOLD:AAN3552
Polistes albellus|BC ZSM HYM 05009|M|Germany|Bavaria|BOLD:AAN3553
Polistes albellus|BC ZSM HYM 05011|M|Germany|Bavaria|BOLD:AAN3553
Polistes albellus|BC ZSM HYM 15513|F|Switzerland||BOLD:AAN3553
Polistes semenowi|BC ZSM HYM 15529|F|Italy|Veneto|BOLD:ACG1290
Polistes semenowi|BC ZSM HYM 22337|M|Switzerland|Ticino|BOLD:ACG1290
Polistes semenowi|BC ZSM HYM 15530|M|Italy|Trentino-Alto Adige|BOLD:ACG1290
Polistes atrimandibularis|BC ZSM HYM 05020|M|Italy|Abruzzi|BOLD:AAN4297
Polistes atrimandibularis|BC ZSM HYM 05019|M|Italy|Abruzzi|BOLD:AAN4297
Polistes austroccidentalis|BC ZSM HYM 22042|M|Morocco||BOLD:ACG1677
Polistes austroccidentalis|BC ZSM HYM 22339|M|Switzerland|Valais|BOLD:ACG1677
Polistes austroccidentalis|BC ZSM HYM 15527|M|France|Provence-Alpes-Cote d'Azur|BOLD:ACG1677
Polistes dominula|BC ZSM HYM 05003|F|France|Provence-Alpes-Cote d'Azur|BOLD:AAA9495
Polistes dominula|BC ZSM HYM 00044|M|Germany|Baden-Wuerttemberg|BOLD:AAA9495
Polistes dominula|BC ZSM HYM 15565|F|Germany|Baden-Wuerttemberg|BOLD:AAA9495
Polistes nimpha|BC ZSM HYM 14012|F|Germany|Baden-Wuerttemberg|BOLD:AAL0103
Polistes nimpha|BC ZSM HYM 19398|F|Germany|Baden-Wuerttemberg|BOLD:AAL0103
Polistes nimpha|BC ZSM HYM 19397|F|Germany|Baden-Wuerttemberg|BOLD:AAL0103
Polistes nimpha|BC ZSM HYM 14018|F|Germany|Brandenburg|BOLD:ACC1661
Polistes nimpha|BC ZSM HYM 14017|F|Germany|Thuringia|BOLD:ACC1661
Polistes nimpha|BC ZSM HYM 14013|F|Germany|Thuringia|BOLD:ACC1661
Polistes dominula|BC ZSM HYM 19392|M|Germany|Baden-Wuerttemberg|BOLD:AAB7105
Polistes dominula|BC ZSM HYM 19394|M|Germany|Baden-Wuerttemberg|BOLD:AAB7105
Polistes dominula|BC ZSM HYM 10201|M|Italy|Veneto|BOLD:AAB7105
Vespa orientalis|BC ZSM HYM 21139|F|Cyprus||BOLD:ACR5399
Vespa orientalis|BC ZSM HYM 21138|F|Cyprus||BOLD:ACR5399
Vespa velutina|BC ZSM HYM 10631|F|France|Nouvelle-Aquitaine|BOLD:AAQ3010
Vespa velutina|BC ZSM HYM 10628|F|France|Nouvelle-Aquitaine|BOLD:AAQ3010
Vespa velutina|BC ZSM HYM 10630|F|France|Nouvelle-Aquitaine|BOLD:AAQ3010
Vespa velutina|BC ZSM HYM 10629|F|France|Nouvelle-Aquitaine|BOLD:AAQ3010
Vespa crabro|BC ZSM HYM 13037|M|Germany|Saxony|BOLD:ABA8441
Vespa crabro|BC ZSM HYM 13038|M|Germany|Saxony|BOLD:ABA8441
Vespa crabro|BC ZSM HYM 13036|F|Germany|Thuringia|BOLD:ABA8441
Vespa crabro|BC ZSM HYM 15275|F|Germany|Brandenburg|BOLD:ABA8441
Vespa crabro|BC ZSM HYM 14389|F|Germany|Brandenburg|BOLD:ABA8441
Vespa crabro|BC ZSM HYM 14388|F|Germany|Brandenburg|BOLD:ABA8441
Vespa crabro|BC ZSM HYM 14387|F|Germany|Brandenburg|BOLD:ABA8441
Vespa crabro|GBOL09095||Hungary|Zala|BOLD:ABA8441
Vespula austriaca|BC ZSM HYM 05028|F|Germany|Bavaria|BOLD:AAN3441
Vespula austriaca|BC ZSM HYM 05025|F|Germany|Bavaria|BOLD:AAN3441
Vespula austriaca|BC ZSM HYM 05027|F|Germany|Bavaria|BOLD:AAN3441
Vespula austriaca|BC ZSM HYM 05026|F|Germany|Bavaria|BOLD:AAN3441
Vespula austriaca|BC ZSM HYM 17209|M|Germany|Bavaria|BOLD:AAN3441

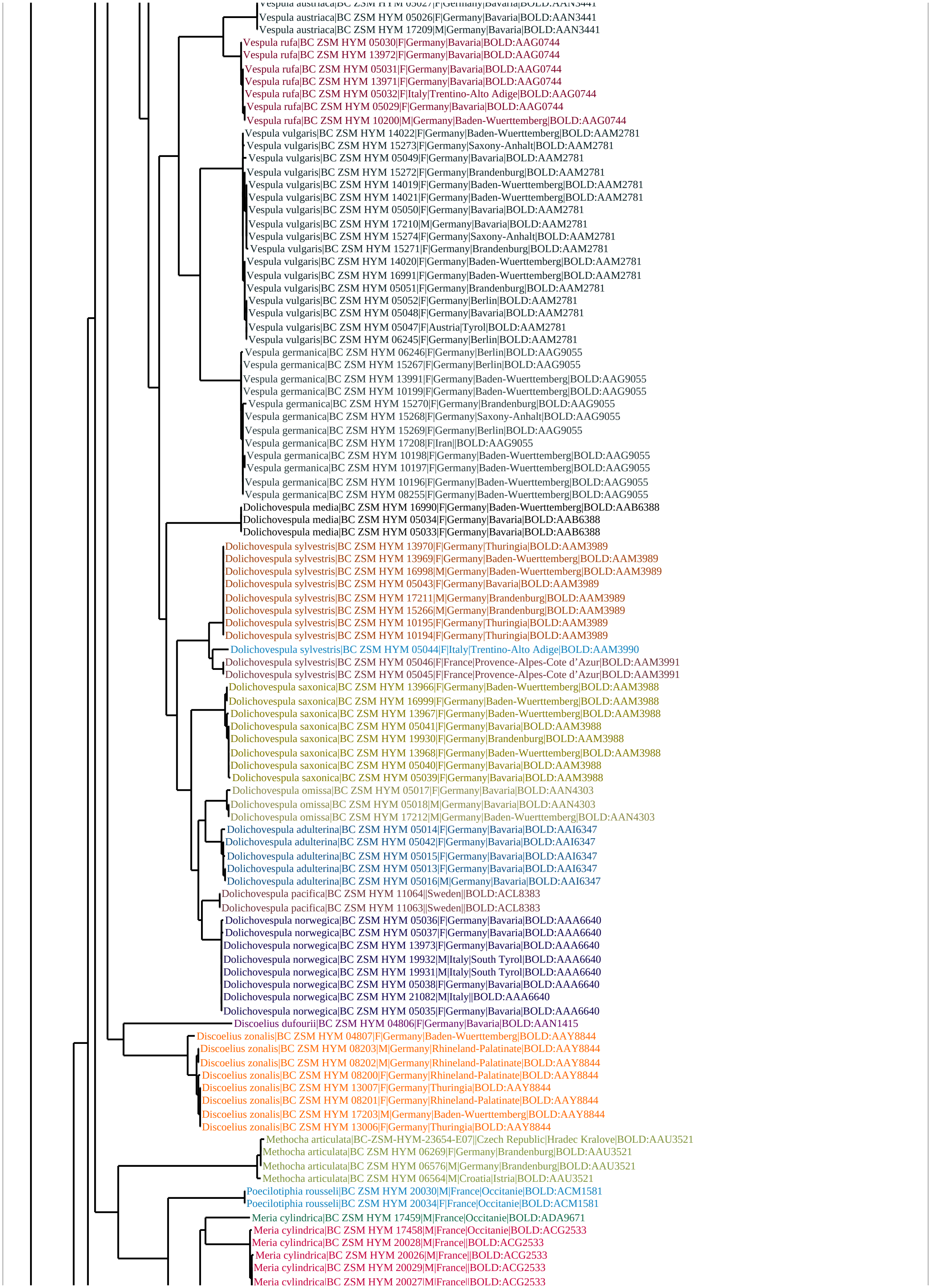
Vespula austriaca|BC ZSM HYM 05027|F|Germany|Bavaria|BOLD:AAN3441
Vespula austriaca|BC ZSM HYM 05026|F|Germany|Bavaria|BOLD:AAN3441
Vespula austriaca|BC ZSM HYM 17209|M|Germany|Bavaria|BOLD:AAN3441
Vespula rufa|BC ZSM HYM 05030|F|Germany|Bavaria|BOLD:AAG0744
Vespula rufa|BC ZSM HYM 13972|F|Germany|Bavaria|BOLD:AAG0744
Vespula rufa|BC ZSM HYM 05031|F|Germany|Bavaria|BOLD:AAG0744
Vespula rufa|BC ZSM HYM 13971|F|Germany|Bavaria|BOLD:AAG0744
Vespula rufa|BC ZSM HYM 05032|F|Italy|Trentino-Alto Adige|BOLD:AAG0744
Vespula rufa|BC ZSM HYM 05029|F|Germany|Bavaria|BOLD:AAG0744
Vespula rufa|BC ZSM HYM 10200|M|Germany|Baden-Wuerttemberg|BOLD:AAG0744
Vespula vulgaris|BC ZSM HYM 14022|F|Germany|Baden-Wuerttemberg|BOLD:AAM2781
Vespula vulgaris|BC ZSM HYM 15273|F|Germany|Saxony-Anhalt|BOLD:AAM2781
Vespula vulgaris|BC ZSM HYM 05049|F|Germany|Bavaria|BOLD:AAM2781
Vespula vulgaris|BC ZSM HYM 15272|F|Germany|Brandenburg|BOLD:AAM2781
Vespula vulgaris|BC ZSM HYM 14019|F|Germany|Baden-Wuerttemberg|BOLD:AAM2781
Vespula vulgaris|BC ZSM HYM 14021|F|Germany|Baden-Wuerttemberg|BOLD:AAM2781
Vespula vulgaris|BC ZSM HYM 05050|F|Germany|Bavaria|BOLD:AAM2781
Vespula vulgaris|BC ZSM HYM 17210|M|Germany|Bavaria|BOLD:AAM2781
Vespula vulgaris|BC ZSM HYM 15274|F|Germany|Saxony-Anhalt|BOLD:AAM2781
Vespula vulgaris|BC ZSM HYM 15271|F|Germany|Brandenburg|BOLD:AAM2781
Vespula vulgaris|BC ZSM HYM 14020|F|Germany|Baden-Wuerttemberg|BOLD:AAM2781
Vespula vulgaris|BC ZSM HYM 16991|F|Germany|Baden-Wuerttemberg|BOLD:AAM2781
Vespula vulgaris|BC ZSM HYM 05051|F|Germany|Brandenburg|BOLD:AAM2781
Vespula vulgaris|BC ZSM HYM 05052|F|Germany|Berlin|BOLD:AAM2781
Vespula vulgaris|BC ZSM HYM 05048|F|Germany|Bavaria|BOLD:AAM2781
Vespula vulgaris|BC ZSM HYM 05047|F|Austria|Tyrol|BOLD:AAM2781
Vespula vulgaris|BC ZSM HYM 06245|F|Germany|Berlin|BOLD:AAM2781
Vespula germanica|BC ZSM HYM 06246|F|Germany|Berlin|BOLD:AAG9055
Vespula germanica|BC ZSM HYM 15267|F|Germany|Berlin|BOLD:AAG9055
Vespula germanica|BC ZSM HYM 13991|F|Germany|Baden-Wuerttemberg|BOLD:AAG9055
Vespula germanica|BC ZSM HYM 10199|F|Germany|Baden-Wuerttemberg|BOLD:AAG9055
Vespula germanica|BC ZSM HYM 15270|F|Germany|Brandenburg|BOLD:AAG9055
Vespula germanica|BC ZSM HYM 15268|F|Germany|Saxony-Anhalt|BOLD:AAG9055
Vespula germanica|BC ZSM HYM 15269|F|Germany|Berlin|BOLD:AAG9055
Vespula germanica|BC ZSM HYM 17208|F|Iran||BOLD:AAG9055
Vespula germanica|BC ZSM HYM 10198|F|Germany|Baden-Wuerttemberg|BOLD:AAG9055
Vespula germanica|BC ZSM HYM 10197|F|Germany|Baden-Wuerttemberg|BOLD:AAG9055
Vespula germanica|BC ZSM HYM 10196|F|Germany|Baden-Wuerttemberg|BOLD:AAG9055
Vespula germanica|BC ZSM HYM 08255|F|Germany|Baden-Wuerttemberg|BOLD:AAG9055
Dolichovespula media|BC ZSM HYM 16990|F|Germany|Baden-Wuerttemberg|BOLD:AAB6388
Dolichovespula media|BC ZSM HYM 05034|F|Germany|Bavaria|BOLD:AAB6388
Dolichovespula media|BC ZSM HYM 05033|F|Germany|Bavaria|BOLD:AAB6388
Dolichovespula sylvestris|BC ZSM HYM 13970|F|Germany|Thuringia|BOLD:AAM3989
Dolichovespula sylvestris|BC ZSM HYM 13969|F|Germany|Baden-Wuerttemberg|BOLD:AAM3989
Dolichovespula sylvestris|BC ZSM HYM 16998|M|Germany|Baden-Wuerttemberg|BOLD:AAM3989
Dolichovespula sylvestris|BC ZSM HYM 05043|F|Germany|Bavaria|BOLD:AAM3989
Dolichovespula sylvestris|BC ZSM HYM 17211|M|Germany|Brandenburg|BOLD:AAM3989
Dolichovespula sylvestris|BC ZSM HYM 15266|M|Germany|Brandenburg|BOLD:AAM3989
Dolichovespula sylvestris|BC ZSM HYM 10195|F|Germany|Thuringia|BOLD:AAM3989
Dolichovespula sylvestris|BC ZSM HYM 10194|F|Germany|Thuringia|BOLD:AAM3989
Dolichovespula sylvestris|BC ZSM HYM 05044|F|Italy|Trentino-Alto Adige|BOLD:AAM3990
Dolichovespula sylvestris|BC ZSM HYM 05046|F|France|Provence-Alpes-Cote d'Azur|BOLD:AAM3991
Dolichovespula sylvestris|BC ZSM HYM 05045|F|France|Provence-Alpes-Cote d'Azur|BOLD:AAM3991
Dolichovespula saxonica|BC ZSM HYM 13966|F|Germany|Baden-Wuerttemberg|BOLD:AAM3988
Dolichovespula saxonica|BC ZSM HYM 16999|F|Germany|Baden-Wuerttemberg|BOLD:AAM3988
Dolichovespula saxonica|BC ZSM HYM 13967|F|Germany|Baden-Wuerttemberg|BOLD:AAM3988
Dolichovespula saxonica|BC ZSM HYM 05041|F|Germany|Bavaria|BOLD:AAM3988
Dolichovespula saxonica|BC ZSM HYM 19930|F|Germany|Brandenburg|BOLD:AAM3988
Dolichovespula saxonica|BC ZSM HYM 13968|F|Germany|Baden-Wuerttemberg|BOLD:AAM3988
Dolichovespula saxonica|BC ZSM HYM 05040|F|Germany|Bavaria|BOLD:AAM3988
Dolichovespula saxonica|BC ZSM HYM 05039|F|Germany|Bavaria|BOLD:AAM3988
Dolichovespula omissa|BC ZSM HYM 05017|F|Germany|Bavaria|BOLD:AAN4303
Dolichovespula omissa|BC ZSM HYM 05018|M|Germany|Bavaria|BOLD:AAN4303
Dolichovespula omissa|BC ZSM HYM 17212|M|Germany|Baden-Wuerttemberg|BOLD:AAN4303
Dolichovespula adulterina|BC ZSM HYM 05014|F|Germany|Bavaria|BOLD:AAI6347
Dolichovespula adulterina|BC ZSM HYM 05042|F|Germany|Bavaria|BOLD:AAI6347
Dolichovespula adulterina|BC ZSM HYM 05015|F|Germany|Bavaria|BOLD:AAI6347
Dolichovespula adulterina|BC ZSM HYM 05013|F|Germany|Bavaria|BOLD:AAI6347
Dolichovespula adulterina|BC ZSM HYM 05016|M|Germany|Bavaria|BOLD:AAI6347
Dolichovespula pacifica|BC ZSM HYM 11064||Sweden||BOLD:ACL8383
Dolichovespula pacifica|BC ZSM HYM 11063||Sweden||BOLD:ACL8383
Dolichovespula norwegica|BC ZSM HYM 05036|F|Germany|Bavaria|BOLD:AAA6640
Dolichovespula norwegica|BC ZSM HYM 05037|F|Germany|Bavaria|BOLD:AAA6640
Dolichovespula norwegica|BC ZSM HYM 13973|F|Germany|Bavaria|BOLD:AAA6640
Dolichovespula norwegica|BC ZSM HYM 19932|M|Italy|South Tyrol|BOLD:AAA6640
Dolichovespula norwegica|BC ZSM HYM 19931|M|Italy|South Tyrol|BOLD:AAA6640
Dolichovespula norwegica|BC ZSM HYM 05038|F|Germany|Bavaria|BOLD:AAA6640
Dolichovespula norwegica|BC ZSM HYM 21082|M|Italy||BOLD:AAA6640
Dolichovespula norwegica|BC ZSM HYM 05035|F|Germany|Bavaria|BOLD:AAA6640
Discoelius dufourii|BC ZSM HYM 04806|F|Germany|Bavaria|BOLD:AAN1415
Discoelius zonalis|BC ZSM HYM 04807|F|Germany|Baden-Wuerttemberg|BOLD:AAY8844
Discoelius zonalis|BC ZSM HYM 08203|M|Germany|Rhineland-Palatinate|BOLD:AAY8844
Discoelius zonalis|BC ZSM HYM 08202|M|Germany|Rhineland-Palatinate|BOLD:AAY8844
Discoelius zonalis|BC ZSM HYM 08200|F|Germany|Rhineland-Palatinate|BOLD:AAY8844
Discoelius zonalis|BC ZSM HYM 13007|F|Germany|Thuringia|BOLD:AAY8844
Discoelius zonalis|BC ZSM HYM 08201|F|Germany|Rhineland-Palatinate|BOLD:AAY8844
Discoelius zonalis|BC ZSM HYM 17203|M|Germany|Baden-Wuerttemberg|BOLD:AAY8844
Discoelius zonalis|BC ZSM HYM 13006|F|Germany|Thuringia|BOLD:AAY8844
Methocha articulata|BC-ZSM-HYM-23654-E07||Czech Republic|Hradec Kralove|BOLD:AAU3521
Methocha articulata|BC ZSM HYM 06269|F|Germany|Brandenburg|BOLD:AAU3521
Methocha articulata|BC ZSM HYM 06576|M|Germany|Brandenburg|BOLD:AAU3521
Methocha articulata|BC ZSM HYM 06564|M|Croatia|Istria|BOLD:AAU3521
Poecilotiphia rousseli|BC ZSM HYM 20030|M|France|Occitanie|BOLD:ACM1581
Poecilotiphia rousseli|BC ZSM HYM 20034|F|France|Occitanie|BOLD:ACM1581
Meria cylindrica|BC ZSM HYM 17459|M|France|Occitanie|BOLD:ADA9671
Meria cylindrica|BC ZSM HYM 17458|M|France|Occitanie|BOLD:ACG2533
Meria cylindrica|BC ZSM HYM 20028|M|France||BOLD:ACG2533
Meria cylindrica|BC ZSM HYM 20026|M|France||BOLD:ACG2533
Meria cylindrica|BC ZSM HYM 20029|M|France||BOLD:ACG2533
Meria cylindrica|BC ZSM HYM 20027|M|France||BOLD:ACG2533

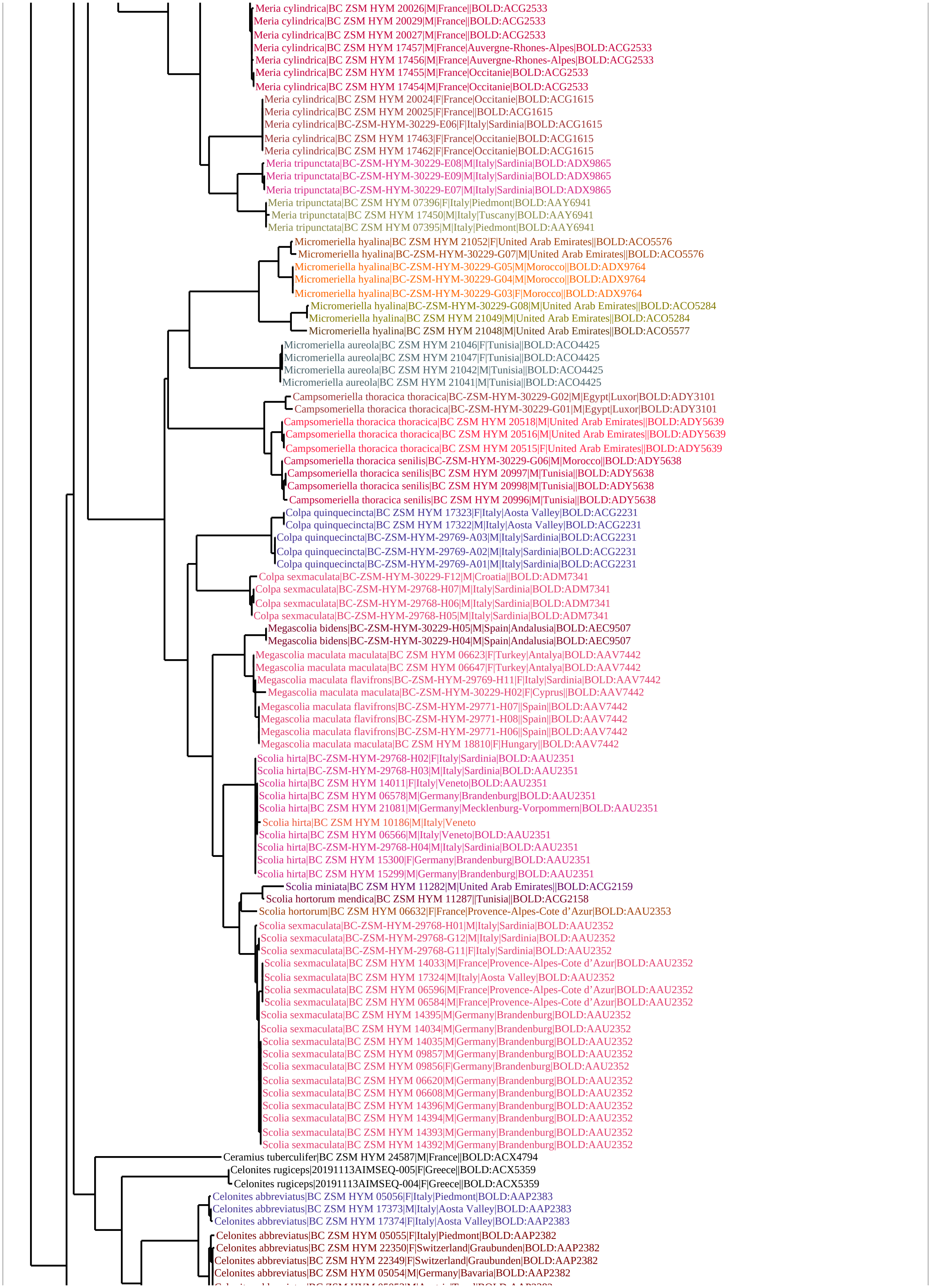
Meria cylindrica|BC ZSM HYM 20026|M|France||BOLD:ACG2533
Meria cylindrica|BC ZSM HYM 20029|M|France||BOLD:ACG2533
Meria cylindrica|BC ZSM HYM 20027|M|France||BOLD:ACG2533
Meria cylindrica|BC ZSM HYM 17457|M|France|Auvergne-Rhones-Alpes|BOLD:ACG2533
Meria cylindrica|BC ZSM HYM 17456|M|France|Auvergne-Rhones-Alpes|BOLD:ACG2533
Meria cylindrica|BC ZSM HYM 17455|M|France|Occitanie|BOLD:ACG2533
Meria cylindrica|BC ZSM HYM 17454|M|France|Occitanie|BOLD:ACG2533
Meria cylindrica|BC ZSM HYM 20024|F|France|Occitanie|BOLD:ACG1615
Meria cylindrica|BC ZSM HYM 20025|F|France||BOLD:ACG1615
Meria cylindrica|BC-ZSM-HYM-30229-E06|F|Italy|Sardinia|BOLD:ACG1615
Meria cylindrica|BC ZSM HYM 17463|F|France|Occitanie|BOLD:ACG1615
Meria cylindrica|BC ZSM HYM 17462|F|France|Occitanie|BOLD:ACG1615
Meria tripunctata|BC-ZSM-HYM-30229-E08|M|Italy|Sardinia|BOLD:ADX9865
Meria tripunctata|BC-ZSM-HYM-30229-E09|M|Italy|Sardinia|BOLD:ADX9865
Meria tripunctata|BC-ZSM-HYM-30229-E07|M|Italy|Sardinia|BOLD:ADX9865
Meria tripunctata|BC ZSM HYM 07396|F|Italy|Piedmont|BOLD:AAY6941
Meria tripunctata|BC ZSM HYM 17450|M|Italy|Tuscany|BOLD:AAY6941
Meria tripunctata|BC ZSM HYM 07395|M|Italy|Piedmont|BOLD:AAY6941
Micromeriella hyalina|BC ZSM HYM 21052|F|United Arab Emirates||BOLD:ACO5576
Micromeriella hyalina|BC-ZSM-HYM-30229-G07|M|United Arab Emirates||BOLD:ACO5576
Micromeriella hyalina|BC-ZSM-HYM-30229-G05|M|Morocco||BOLD:ADX9764
Micromeriella hyalina|BC-ZSM-HYM-30229-G04|M|Morocco||BOLD:ADX9764
Micromeriella hyalina|BC-ZSM-HYM-30229-G03|F|Morocco||BOLD:ADX9764
Micromeriella hyalina|BC-ZSM-HYM-30229-G08|M|United Arab Emirates||BOLD:ACO5284
Micromeriella hyalina|BC ZSM HYM 21049|M|United Arab Emirates||BOLD:ACO5284
Micromeriella hyalina|BC ZSM HYM 21048|M|United Arab Emirates||BOLD:ACO5577
Micromeriella aureola|BC ZSM HYM 21046|F|Tunisia||BOLD:ACO4425
Micromeriella aureola|BC ZSM HYM 21047|F|Tunisia||BOLD:ACO4425
Micromeriella aureola|BC ZSM HYM 21042|M|Tunisia||BOLD:ACO4425
Micromeriella aureola|BC ZSM HYM 21041|M|Tunisia||BOLD:ACO4425
Campsomeriella thoracica thoracica|BC-ZSM-HYM-30229-G02|M|Egypt|Luxor|BOLD:ADY3101
Campsomeriella thoracica thoracica|BC-ZSM-HYM-30229-G01|M|Egypt|Luxor|BOLD:ADY3101
Campsomeriella thoracica thoracica|BC ZSM HYM 20518|M|United Arab Emirates||BOLD:ADY5639
Campsomeriella thoracica thoracica|BC ZSM HYM 20516|M|United Arab Emirates||BOLD:ADY5639
Campsomeriella thoracica thoracica|BC ZSM HYM 20515|F|United Arab Emirates||BOLD:ADY5639
Campsomeriella thoracica senilis|BC-ZSM-HYM-30229-G06|M|Morocco||BOLD:ADY5638
Campsomeriella thoracica senilis|BC ZSM HYM 20997|M|Tunisia||BOLD:ADY5638
Campsomeriella thoracica senilis|BC ZSM HYM 20998|M|Tunisia||BOLD:ADY5638
Campsomeriella thoracica senilis|BC ZSM HYM 20996|M|Tunisia||BOLD:ADY5638
Colpa quinquecincta|BC ZSM HYM 17323|F|Italy|Aosta Valley|BOLD:ACG2231
Colpa quinquecincta|BC ZSM HYM 17322|M|Italy|Aosta Valley|BOLD:ACG2231
Colpa quinquecincta|BC-ZSM-HYM-29769-A03|M|Italy|Sardinia|BOLD:ACG2231
Colpa quinquecincta|BC-ZSM-HYM-29769-A02|M|Italy|Sardinia|BOLD:ACG2231
Colpa quinquecincta|BC-ZSM-HYM-29769-A01|M|Italy|Sardinia|BOLD:ACG2231
Colpa sexmaculata|BC-ZSM-HYM-30229-F12|M|Croatia||BOLD:ADM7341
Colpa sexmaculata|BC-ZSM-HYM-29768-H07|M|Italy|Sardinia|BOLD:ADM7341
Colpa sexmaculata|BC-ZSM-HYM-29768-H06|M|Italy|Sardinia|BOLD:ADM7341
Colpa sexmaculata|BC-ZSM-HYM-29768-H05|M|Italy|Sardinia|BOLD:ADM7341
Megascolia bidens|BC-ZSM-HYM-30229-H05|M|Spain|Andalusia|BOLD:AEC9507
Megascolia bidens|BC-ZSM-HYM-30229-H04|M|Spain|Andalusia|BOLD:AEC9507
Megascolia maculata maculata|BC ZSM HYM 06623|F|Turkey|Antalya|BOLD:AAV7442
Megascolia maculata maculata|BC ZSM HYM 06647|F|Turkey|Antalya|BOLD:AAV7442
Megascolia maculata flavifrons|BC-ZSM-HYM-29769-H11|F|Italy|Sardinia|BOLD:AAV7442
Megascolia maculata maculata|BC-ZSM-HYM-30229-H02|F|Cyprus||BOLD:AAV7442
Megascolia maculata flavifrons|BC-ZSM-HYM-29771-H07||Spain||BOLD:AAV7442
Megascolia maculata flavifrons|BC-ZSM-HYM-29771-H08||Spain||BOLD:AAV7442
Megascolia maculata flavifrons|BC-ZSM-HYM-29771-H06||Spain||BOLD:AAV7442
Megascolia maculata maculata|BC ZSM HYM 18810|F|Hungary||BOLD:AAV7442
Scolia hirta|BC-ZSM-HYM-29768-H02|F|Italy|Sardinia|BOLD:AAU2351
Scolia hirta|BC-ZSM-HYM-29768-H03|M|Italy|Sardinia|BOLD:AAU2351
Scolia hirta|BC ZSM HYM 14011|F|Italy|Veneto|BOLD:AAU2351
Scolia hirta|BC ZSM HYM 06578|M|Germany|Brandenburg|BOLD:AAU2351
Scolia hirta|BC ZSM HYM 21081|M|Germany|Mecklenburg-Vorpommern|BOLD:AAU2351
Scolia hirta|BC ZSM HYM 10186|M|Italy|Veneto
Scolia hirta|BC ZSM HYM 06566|M|Italy|Veneto|BOLD:AAU2351
Scolia hirta|BC-ZSM-HYM-29768-H04|M|Italy|Sardinia|BOLD:AAU2351
Scolia hirta|BC ZSM HYM 15300|F|Germany|Brandenburg|BOLD:AAU2351
Scolia hirta|BC ZSM HYM 15299|M|Germany|Brandenburg|BOLD:AAU2351
Scolia miniata|BC ZSM HYM 11282|M|United Arab Emirates||BOLD:ACG2159
Scolia hortorum mendica|BC ZSM HYM 11287||Tunisia||BOLD:ACG2158
Scolia hortorum|BC ZSM HYM 06632|F|France|Provence-Alpes-Cote d'Azur|BOLD:AAU2353
Scolia sexmaculata|BC-ZSM-HYM-29768-H01|M|Italy|Sardinia|BOLD:AAU2352
Scolia sexmaculata|BC-ZSM-HYM-29768-G12|M|Italy|Sardinia|BOLD:AAU2352
Scolia sexmaculata|BC-ZSM-HYM-29768-G11|F|Italy|Sardinia|BOLD:AAU2352
Scolia sexmaculata|BC ZSM HYM 14033|M|France|Provence-Alpes-Cote d'Azur|BOLD:AAU2352
Scolia sexmaculata|BC ZSM HYM 17324|M|Italy|Aosta Valley|BOLD:AAU2352
Scolia sexmaculata|BC ZSM HYM 06596|M|France|Provence-Alpes-Cote d'Azur|BOLD:AAU2352
Scolia sexmaculata|BC ZSM HYM 06584|M|France|Provence-Alpes-Cote d'Azur|BOLD:AAU2352
Scolia sexmaculata|BC ZSM HYM 14395|M|Germany|Brandenburg|BOLD:AAU2352
Scolia sexmaculata|BC ZSM HYM 14034|M|Germany|Brandenburg|BOLD:AAU2352
Scolia sexmaculata|BC ZSM HYM 14035|M|Germany|Brandenburg|BOLD:AAU2352
Scolia sexmaculata|BC ZSM HYM 09857|M|Germany|Brandenburg|BOLD:AAU2352
Scolia sexmaculata|BC ZSM HYM 09856|F|Germany|Brandenburg|BOLD:AAU2352
Scolia sexmaculata|BC ZSM HYM 06620|M|Germany|Brandenburg|BOLD:AAU2352
Scolia sexmaculata|BC ZSM HYM 06608|M|Germany|Brandenburg|BOLD:AAU2352
Scolia sexmaculata|BC ZSM HYM 14396|M|Germany|Brandenburg|BOLD:AAU2352
Scolia sexmaculata|BC ZSM HYM 14394|M|Germany|Brandenburg|BOLD:AAU2352
Scolia sexmaculata|BC ZSM HYM 14393|M|Germany|Brandenburg|BOLD:AAU2352
Scolia sexmaculata|BC ZSM HYM 14392|M|Germany|Brandenburg|BOLD:AAU2352
Ceramius tuberculifer|BC ZSM HYM 24587|M|France||BOLD:ACX4794
Celonites rugiceps|20191113AIMSEQ-005|F|Greece||BOLD:ACX5359
Celonites rugiceps|20191113AIMSEQ-004|F|Greece||BOLD:ACX5359
Celonites abbreviatus|BC ZSM HYM 05056|F|Italy|Piedmont|BOLD:AAP2383
Celonites abbreviatus|BC ZSM HYM 17373|M|Italy|Aosta Valley|BOLD:AAP2383
Celonites abbreviatus|BC ZSM HYM 17374|F|Italy|Aosta Valley|BOLD:AAP2383
Celonites abbreviatus|BC ZSM HYM 05055|F|Italy|Piedmont|BOLD:AAP2382
Celonites abbreviatus|BC ZSM HYM 22350|F|Switzerland|Graubunden|BOLD:AAP2382
Celonites abbreviatus|BC ZSM HYM 22349|F|Switzerland|Graubunden|BOLD:AAP2382
Celonites abbreviatus|BC ZSM HYM 05054|M|Germany|Bavaria|BOLD:AAP2382
[illegible]

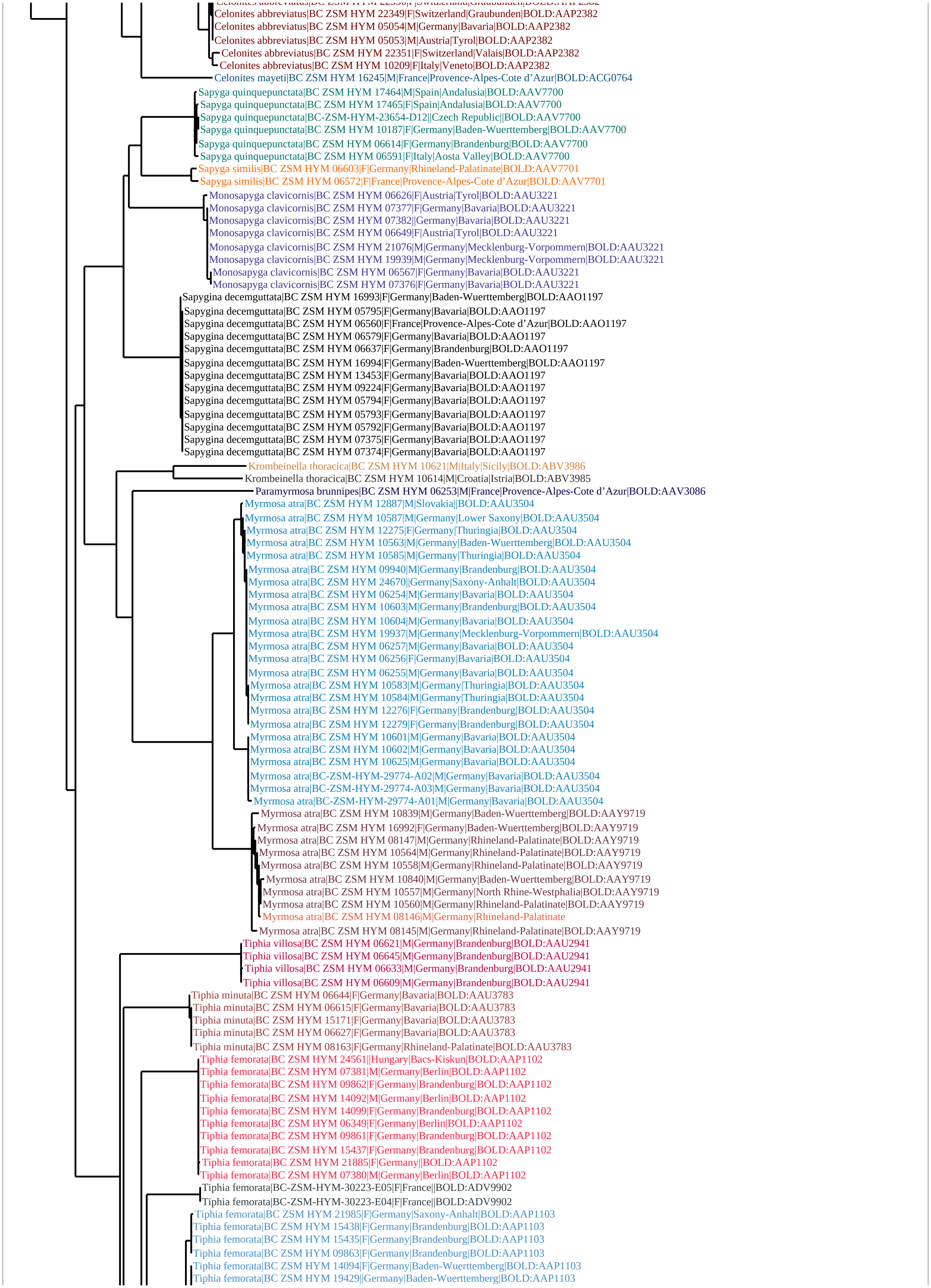
Celonites abbreviatus|BC ZSM HYM 22349|F|Switzerland|Graubunden|BOLD:AAP2382
Celonites abbreviatus|BC ZSM HYM 05054|M|Germany|Bavaria|BOLD:AAP2382
Celonites abbreviatus|BC ZSM HYM 05053|M|Austria|Tyrol|BOLD:AAP2382
Celonites abbreviatus|BC ZSM HYM 22351|F|Switzerland|Valais|BOLD:AAP2382
Celonites abbreviatus|BC ZSM HYM 10209|F|Italy|Veneto|BOLD:AAP2382
Celonites mayeti|BC ZSM HYM 16245|M|France|Provence-Alpes-Cote d'Azur|BOLD:ACG0764
Sapyga quinquepunctata|BC ZSM HYM 17464|M|Spain|Andalusia|BOLD:AAV7700
Sapyga quinquepunctata|BC ZSM HYM 17465|F|Spain|Andalusia|BOLD:AAV7700
Sapyga quinquepunctata|BC-ZSM-HYM-23654-D12||Czech Republic||BOLD:AAV7700
Sapyga quinquepunctata|BC ZSM HYM 10187|F|Germany|Baden-Wuerttemberg|BOLD:AAV7700
Sapyga quinquepunctata|BC ZSM HYM 06614|F|Germany|Brandenburg|BOLD:AAV7700
Sapyga quinquepunctata|BC ZSM HYM 06591|F|Italy|Aosta Valley|BOLD:AAV7700
Sapyga similis|BC ZSM HYM 06603|F|Germany|Rhineland-Palatinate|BOLD:AAV7701
Sapyga similis|BC ZSM HYM 06572|F|France|Provence-Alpes-Cote d'Azur|BOLD:AAV7701
Monosapyga clavicornis|BC ZSM HYM 06626|F|Austria|Tyrol|BOLD:AAU3221
Monosapyga clavicornis|BC ZSM HYM 07377|F|Germany|Bavaria|BOLD:AAU3221
Monosapyga clavicornis|BC ZSM HYM 07382||Germany|Bavaria|BOLD:AAU3221
Monosapyga clavicornis|BC ZSM HYM 06649|F|Austria|Tyrol|BOLD:AAU3221
Monosapyga clavicornis|BC ZSM HYM 21076|M|Germany|Mecklenburg-Vorpommern|BOLD:AAU3221
Monosapyga clavicornis|BC ZSM HYM 19939|M|Germany|Mecklenburg-Vorpommern|BOLD:AAU3221
Monosapyga clavicornis|BC ZSM HYM 06567|F|Germany|Bavaria|BOLD:AAU3221
Monosapyga clavicornis|BC ZSM HYM 07376|F|Germany|Bavaria|BOLD:AAU3221
Sapygina decemguttata|BC ZSM HYM 16993|F|Germany|Baden-Wuerttemberg|BOLD:AAO1197
Sapygina decemguttata|BC ZSM HYM 05795|F|Germany|Bavaria|BOLD:AAO1197
Sapygina decemguttata|BC ZSM HYM 06560|F|France|Provence-Alpes-Cote d'Azur|BOLD:AAO1197
Sapygina decemguttata|BC ZSM HYM 06579|F|Germany|Bavaria|BOLD:AAO1197
Sapygina decemguttata|BC ZSM HYM 06637|F|Germany|Brandenburg|BOLD:AAO1197
Sapygina decemguttata|BC ZSM HYM 16994|F|Germany|Baden-Wuerttemberg|BOLD:AAO1197
Sapygina decemguttata|BC ZSM HYM 13453|F|Germany|Bavaria|BOLD:AAO1197
Sapygina decemguttata|BC ZSM HYM 09224|F|Germany|Bavaria|BOLD:AAO1197
Sapygina decemguttata|BC ZSM HYM 05794|F|Germany|Bavaria|BOLD:AAO1197
Sapygina decemguttata|BC ZSM HYM 05793|F|Germany|Bavaria|BOLD:AAO1197
Sapygina decemguttata|BC ZSM HYM 05792|F|Germany|Bavaria|BOLD:AAO1197
Sapygina decemguttata|BC ZSM HYM 07375|F|Germany|Bavaria|BOLD:AAO1197
Sapygina decemguttata|BC ZSM HYM 07374|F|Germany|Bavaria|BOLD:AAO1197
Krombeinella thoracica|BC ZSM HYM 10621|M|Italy|Sicily|BOLD:ABV3986
Krombeinella thoracica|BC ZSM HYM 10614|M|Croatia|Istria|BOLD:ABV3985
Paramyrmosa brunnipes|BC ZSM HYM 06253|M|France|Provence-Alpes-Cote d'Azur|BOLD:AAV3086
Myrmosa atra|BC ZSM HYM 12887|M|Slovakia||BOLD:AAU3504
Myrmosa atra|BC ZSM HYM 10587|M|Germany|Lower Saxony|BOLD:AAU3504
Myrmosa atra|BC ZSM HYM 12275|F|Germany|Thuringia|BOLD:AAU3504
Myrmosa atra|BC ZSM HYM 10563|M|Germany|Baden-Wuerttemberg|BOLD:AAU3504
Myrmosa atra|BC ZSM HYM 10585|M|Germany|Thuringia|BOLD:AAU3504
Myrmosa atra|BC ZSM HYM 09940|M|Germany|Brandenburg|BOLD:AAU3504
Myrmosa atra|BC ZSM HYM 24670||Germany|Saxony-Anhalt|BOLD:AAU3504
Myrmosa atra|BC ZSM HYM 06254|M|Germany|Bavaria|BOLD:AAU3504
Myrmosa atra|BC ZSM HYM 10603|M|Germany|Brandenburg|BOLD:AAU3504
Myrmosa atra|BC ZSM HYM 10604|M|Germany|Bavaria|BOLD:AAU3504
Myrmosa atra|BC ZSM HYM 19937|M|Germany|Mecklenburg-Vorpommern|BOLD:AAU3504
Myrmosa atra|BC ZSM HYM 06257|M|Germany|Bavaria|BOLD:AAU3504
Myrmosa atra|BC ZSM HYM 06256|F|Germany|Bavaria|BOLD:AAU3504
Myrmosa atra|BC ZSM HYM 06255|M|Germany|Bavaria|BOLD:AAU3504
Myrmosa atra|BC ZSM HYM 10583|M|Germany|Thuringia|BOLD:AAU3504
Myrmosa atra|BC ZSM HYM 10584|M|Germany|Thuringia|BOLD:AAU3504
Myrmosa atra|BC ZSM HYM 12276|F|Germany|Brandenburg|BOLD:AAU3504
Myrmosa atra|BC ZSM HYM 12279|F|Germany|Brandenburg|BOLD:AAU3504
Myrmosa atra|BC ZSM HYM 10601|M|Germany|Bavaria|BOLD:AAU3504
Myrmosa atra|BC ZSM HYM 10602|M|Germany|Bavaria|BOLD:AAU3504
Myrmosa atra|BC ZSM HYM 10625|M|Germany|Bavaria|BOLD:AAU3504
Myrmosa atra|BC-ZSM-HYM-29774-A02|M|Germany|Bavaria|BOLD:AAU3504
Myrmosa atra|BC-ZSM-HYM-29774-A03|M|Germany|Bavaria|BOLD:AAU3504
Myrmosa atra|BC-ZSM-HYM-29774-A01|M|Germany|Bavaria|BOLD:AAU3504
Myrmosa atra|BC ZSM HYM 10839|M|Germany|Baden-Wuerttemberg|BOLD:AAY9719
Myrmosa atra|BC ZSM HYM 16992|F|Germany|Baden-Wuerttemberg|BOLD:AAY9719
Myrmosa atra|BC ZSM HYM 08147|M|Germany|Rhineland-Palatinate|BOLD:AAY9719
Myrmosa atra|BC ZSM HYM 10564|M|Germany|Rhineland-Palatinate|BOLD:AAY9719
Myrmosa atra|BC ZSM HYM 10558|M|Germany|Rhineland-Palatinate|BOLD:AAY9719
Myrmosa atra|BC ZSM HYM 10840|M|Germany|Baden-Wuerttemberg|BOLD:AAY9719
Myrmosa atra|BC ZSM HYM 10557|M|Germany|North Rhine-Westphalia|BOLD:AAY9719
Myrmosa atra|BC ZSM HYM 10560|M|Germany|Rhineland-Palatinate|BOLD:AAY9719
Myrmosa atra|BC ZSM HYM 08146|M|Germany|Rhineland-Palatinate
Myrmosa atra|BC ZSM HYM 08145|M|Germany|Rhineland-Palatinate|BOLD:AAY9719
Tiphia villosa|BC ZSM HYM 06621|M|Germany|Brandenburg|BOLD:AAU2941
Tiphia villosa|BC ZSM HYM 06645|M|Germany|Brandenburg|BOLD:AAU2941
Tiphia villosa|BC ZSM HYM 06633|M|Germany|Brandenburg|BOLD:AAU2941
Tiphia villosa|BC ZSM HYM 06609|M|Germany|Brandenburg|BOLD:AAU2941
Tiphia minuta|BC ZSM HYM 06644|F|Germany|Bavaria|BOLD:AAU3783
Tiphia minuta|BC ZSM HYM 06615|F|Germany|Bavaria|BOLD:AAU3783
Tiphia minuta|BC ZSM HYM 15171|F|Germany|Bavaria|BOLD:AAU3783
Tiphia minuta|BC ZSM HYM 06627|F|Germany|Bavaria|BOLD:AAU3783
Tiphia minuta|BC ZSM HYM 08163|F|Germany|Rhineland-Palatinate|BOLD:AAU3783
Tiphia femorata|BC ZSM HYM 24561||Hungary|Bacs-Kiskun|BOLD:AAP1102
Tiphia femorata|BC ZSM HYM 07381|M|Germany|Berlin|BOLD:AAP1102
Tiphia femorata|BC ZSM HYM 09862|F|Germany|Brandenburg|BOLD:AAP1102
Tiphia femorata|BC ZSM HYM 14092|M|Germany|Berlin|BOLD:AAP1102
Tiphia femorata|BC ZSM HYM 14099|F|Germany|Brandenburg|BOLD:AAP1102
Tiphia femorata|BC ZSM HYM 06349|F|Germany|Berlin|BOLD:AAP1102
Tiphia femorata|BC ZSM HYM 09861|F|Germany|Brandenburg|BOLD:AAP1102
Tiphia femorata|BC ZSM HYM 15437|F|Germany|Brandenburg|BOLD:AAP1102
Tiphia femorata|BC ZSM HYM 21885|F|Germany||BOLD:AAP1102
Tiphia femorata|BC ZSM HYM 07380|M|Germany|Berlin|BOLD:AAP1102
Tiphia femorata|BC-ZSM-HYM-30223-E05|F|France||BOLD:ADV9902
Tiphia femorata|BC-ZSM-HYM-30223-E04|F|France||BOLD:ADV9902
Tiphia femorata|BC ZSM HYM 21985|F|Germany|Saxony-Anhalt|BOLD:AAP1103
Tiphia femorata|BC ZSM HYM 15438|F|Germany|Brandenburg|BOLD:AAP1103
Tiphia femorata|BC ZSM HYM 15435|F|Germany|Brandenburg|BOLD:AAP1103
Tiphia femorata|BC ZSM HYM 09863|F|Germany|Brandenburg|BOLD:AAP1103
Tiphia femorata|BC ZSM HYM 14094|F|Germany|Baden-Wuerttemberg|BOLD:AAP1103
Tiphia femorata|BC ZSM HYM 19429||Germany|Baden-Wuerttemberg|BOLD:AAP1103

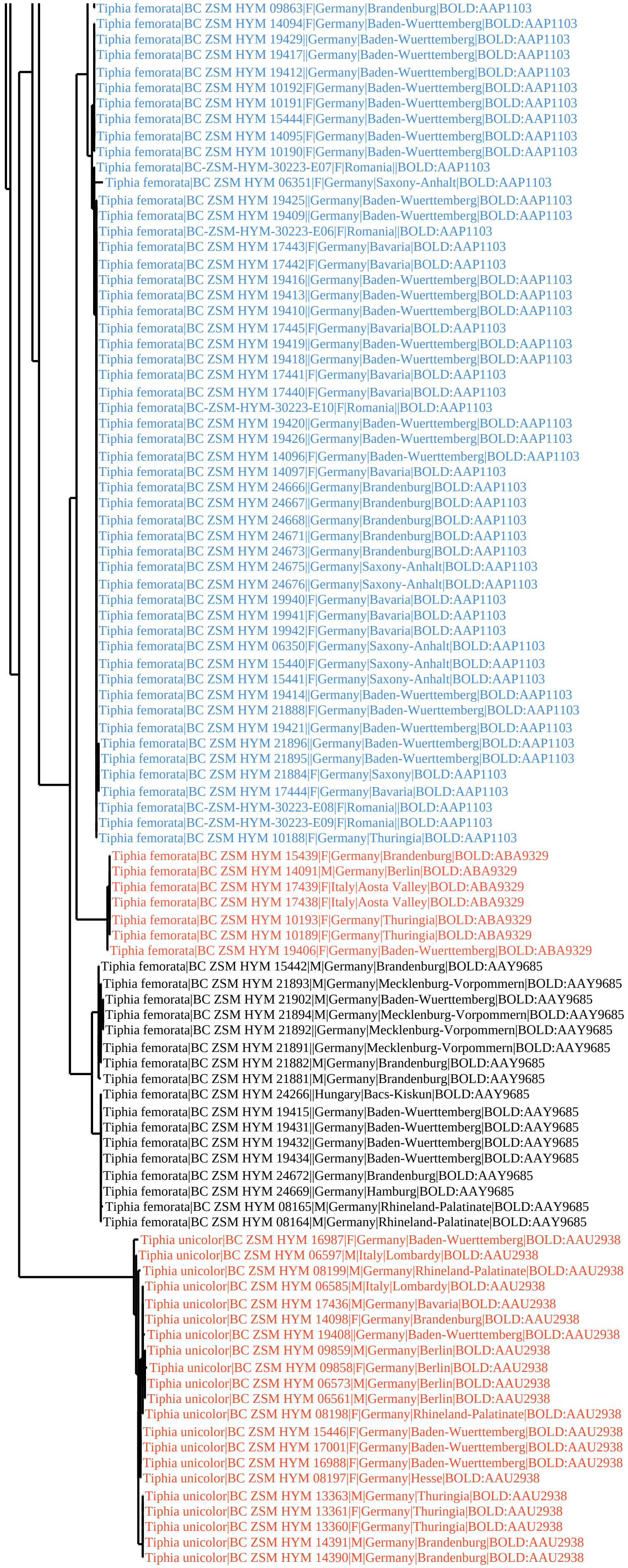
Tiphia femorata|BC ZSM HYM 09863|F|Germany|Brandenburg|BOLD:AAP1103
Tiphia femorata|BC ZSM HYM 14094|F|Germany|Baden-Wuerttemberg|BOLD:AAP1103
Tiphia femorata|BC ZSM HYM 19429||Germany|Baden-Wuerttemberg|BOLD:AAP1103
Tiphia femorata|BC ZSM HYM 19417||Germany|Baden-Wuerttemberg|BOLD:AAP1103
Tiphia femorata|BC ZSM HYM 19412||Germany|Baden-Wuerttemberg|BOLD:AAP1103
Tiphia femorata|BC ZSM HYM 10192|F|Germany|Baden-Wuerttemberg|BOLD:AAP1103
Tiphia femorata|BC ZSM HYM 10191|F|Germany|Baden-Wuerttemberg|BOLD:AAP1103
Tiphia femorata|BC ZSM HYM 15444|F|Germany|Baden-Wuerttemberg|BOLD:AAP1103
Tiphia femorata|BC ZSM HYM 14095|F|Germany|Baden-Wuerttemberg|BOLD:AAP1103
Tiphia femorata|BC ZSM HYM 10190|F|Germany|Baden-Wuerttemberg|BOLD:AAP1103
Tiphia femorata|BC-ZSM-HYM-30223-E07|F|Romania||BOLD:AAP1103
Tiphia femorata|BC ZSM HYM 06351|F|Germany|Saxony-Anhalt|BOLD:AAP1103
Tiphia femorata|BC ZSM HYM 19425||Germany|Baden-Wuerttemberg|BOLD:AAP1103
Tiphia femorata|BC ZSM HYM 19409||Germany|Baden-Wuerttemberg|BOLD:AAP1103
Tiphia femorata|BC-ZSM-HYM-30223-E06|F|Romania||BOLD:AAP1103
Tiphia femorata|BC ZSM HYM 17443|F|Germany|Bavaria|BOLD:AAP1103
Tiphia femorata|BC ZSM HYM 17442|F|Germany|Bavaria|BOLD:AAP1103
Tiphia femorata|BC ZSM HYM 19416||Germany|Baden-Wuerttemberg|BOLD:AAP1103
Tiphia femorata|BC ZSM HYM 19413||Germany|Baden-Wuerttemberg|BOLD:AAP1103
Tiphia femorata|BC ZSM HYM 19410||Germany|Baden-Wuerttemberg|BOLD:AAP1103
Tiphia femorata|BC ZSM HYM 17445|F|Germany|Bavaria|BOLD:AAP1103
Tiphia femorata|BC ZSM HYM 19419||Germany|Baden-Wuerttemberg|BOLD:AAP1103
Tiphia femorata|BC ZSM HYM 19418||Germany|Baden-Wuerttemberg|BOLD:AAP1103
Tiphia femorata|BC ZSM HYM 17441|F|Germany|Bavaria|BOLD:AAP1103
Tiphia femorata|BC ZSM HYM 17440|F|Germany|Bavaria|BOLD:AAP1103
Tiphia femorata|BC-ZSM-HYM-30223-E10|F|Romania||BOLD:AAP1103
Tiphia femorata|BC ZSM HYM 19420||Germany|Baden-Wuerttemberg|BOLD:AAP1103
Tiphia femorata|BC ZSM HYM 19426||Germany|Baden-Wuerttemberg|BOLD:AAP1103
Tiphia femorata|BC ZSM HYM 14096|F|Germany|Baden-Wuerttemberg|BOLD:AAP1103
Tiphia femorata|BC ZSM HYM 14097|F|Germany|Bavaria|BOLD:AAP1103
Tiphia femorata|BC ZSM HYM 24666||Germany|Brandenburg|BOLD:AAP1103
Tiphia femorata|BC ZSM HYM 24667||Germany|Brandenburg|BOLD:AAP1103
Tiphia femorata|BC ZSM HYM 24668||Germany|Brandenburg|BOLD:AAP1103
Tiphia femorata|BC ZSM HYM 24671||Germany|Brandenburg|BOLD:AAP1103
Tiphia femorata|BC ZSM HYM 24673||Germany|Brandenburg|BOLD:AAP1103
Tiphia femorata|BC ZSM HYM 24675||Germany|Saxony-Anhalt|BOLD:AAP1103
Tiphia femorata|BC ZSM HYM 24676||Germany|Saxony-Anhalt|BOLD:AAP1103
Tiphia femorata|BC ZSM HYM 19940|F|Germany|Bavaria|BOLD:AAP1103
Tiphia femorata|BC ZSM HYM 19941|F|Germany|Bavaria|BOLD:AAP1103
Tiphia femorata|BC ZSM HYM 19942|F|Germany|Bavaria|BOLD:AAP1103
Tiphia femorata|BC ZSM HYM 06350|F|Germany|Saxony-Anhalt|BOLD:AAP1103
Tiphia femorata|BC ZSM HYM 15440|F|Germany|Saxony-Anhalt|BOLD:AAP1103
Tiphia femorata|BC ZSM HYM 15441|F|Germany|Saxony-Anhalt|BOLD:AAP1103
Tiphia femorata|BC ZSM HYM 19414||Germany|Baden-Wuerttemberg|BOLD:AAP1103
Tiphia femorata|BC ZSM HYM 21888|F|Germany|Baden-Wuerttemberg|BOLD:AAP1103
Tiphia femorata|BC ZSM HYM 19421||Germany|Baden-Wuerttemberg|BOLD:AAP1103
Tiphia femorata|BC ZSM HYM 21896||Germany|Baden-Wuerttemberg|BOLD:AAP1103
Tiphia femorata|BC ZSM HYM 21895||Germany|Baden-Wuerttemberg|BOLD:AAP1103
Tiphia femorata|BC ZSM HYM 21884|F|Germany|Saxony|BOLD:AAP1103
Tiphia femorata|BC ZSM HYM 17444|F|Germany|Bavaria|BOLD:AAP1103
Tiphia femorata|BC-ZSM-HYM-30223-E08|F|Romania||BOLD:AAP1103
Tiphia femorata|BC-ZSM-HYM-30223-E09|F|Romania||BOLD:AAP1103
Tiphia femorata|BC ZSM HYM 10188|F|Germany|Thuringia|BOLD:AAP1103
Tiphia femorata|BC ZSM HYM 15439|F|Germany|Brandenburg|BOLD:ABA9329
Tiphia femorata|BC ZSM HYM 14091|M|Germany|Berlin|BOLD:ABA9329
Tiphia femorata|BC ZSM HYM 17439|F|Italy|Aosta Valley|BOLD:ABA9329
Tiphia femorata|BC ZSM HYM 17438|F|Italy|Aosta Valley|BOLD:ABA9329
Tiphia femorata|BC ZSM HYM 10193|F|Germany|Thuringia|BOLD:ABA9329
Tiphia femorata|BC ZSM HYM 10189|F|Germany|Thuringia|BOLD:ABA9329
Tiphia femorata|BC ZSM HYM 19406|F|Germany|Baden-Wuerttemberg|BOLD:ABA9329
Tiphia femorata|BC ZSM HYM 15442|M|Germany|Brandenburg|BOLD:AAY9685
Tiphia femorata|BC ZSM HYM 21893|M|Germany|Mecklenburg-Vorpommern|BOLD:AAY9685
Tiphia femorata|BC ZSM HYM 21902|M|Germany|Baden-Wuerttemberg|BOLD:AAY9685
Tiphia femorata|BC ZSM HYM 21894|M|Germany|Mecklenburg-Vorpommern|BOLD:AAY9685
Tiphia femorata|BC ZSM HYM 21892||Germany|Mecklenburg-Vorpommern|BOLD:AAY9685
Tiphia femorata|BC ZSM HYM 21891||Germany|Mecklenburg-Vorpommern|BOLD:AAY9685
Tiphia femorata|BC ZSM HYM 21882|M|Germany|Brandenburg|BOLD:AAY9685
Tiphia femorata|BC ZSM HYM 21881|M|Germany|Brandenburg|BOLD:AAY9685
Tiphia femorata|BC ZSM HYM 24266||Hungary|Bacs-Kiskun|BOLD:AAY9685
Tiphia femorata|BC ZSM HYM 19415||Germany|Baden-Wuerttemberg|BOLD:AAY9685
Tiphia femorata|BC ZSM HYM 19431||Germany|Baden-Wuerttemberg|BOLD:AAY9685
Tiphia femorata|BC ZSM HYM 19432||Germany|Baden-Wuerttemberg|BOLD:AAY9685
Tiphia femorata|BC ZSM HYM 19434||Germany|Baden-Wuerttemberg|BOLD:AAY9685
Tiphia femorata|BC ZSM HYM 24672||Germany|Brandenburg|BOLD:AAY9685
Tiphia femorata|BC ZSM HYM 24669||Germany|Hamburg|BOLD:AAY9685
Tiphia femorata|BC ZSM HYM 08165|M|Germany|Rhineland-Palatinate|BOLD:AAY9685
Tiphia femorata|BC ZSM HYM 08164|M|Germany|Rhineland-Palatinate|BOLD:AAY9685
Tiphia unicolor|BC ZSM HYM 16987|F|Germany|Baden-Wuerttemberg|BOLD:AAU2938
Tiphia unicolor|BC ZSM HYM 06597|M|Italy|Lombardy|BOLD:AAU2938
Tiphia unicolor|BC ZSM HYM 08199|M|Germany|Rhineland-Palatinate|BOLD:AAU2938
Tiphia unicolor|BC ZSM HYM 06585|M|Italy|Lombardy|BOLD:AAU2938
Tiphia unicolor|BC ZSM HYM 17436|M|Germany|Bavaria|BOLD:AAU2938
Tiphia unicolor|BC ZSM HYM 14098|F|Germany|Brandenburg|BOLD:AAU2938
Tiphia unicolor|BC ZSM HYM 19408||Germany|Baden-Wuerttemberg|BOLD:AAU2938
Tiphia unicolor|BC ZSM HYM 09859|M|Germany|Berlin|BOLD:AAU2938
Tiphia unicolor|BC ZSM HYM 09858|F|Germany|Berlin|BOLD:AAU2938
Tiphia unicolor|BC ZSM HYM 06573|M|Germany|Berlin|BOLD:AAU2938
Tiphia unicolor|BC ZSM HYM 06561|M|Germany|Berlin|BOLD:AAU2938
Tiphia unicolor|BC ZSM HYM 08198|F|Germany|Rhineland-Palatinate|BOLD:AAU2938
Tiphia unicolor|BC ZSM HYM 15446|F|Germany|Baden-Wuerttemberg|BOLD:AAU2938
Tiphia unicolor|BC ZSM HYM 17001|F|Germany|Baden-Wuerttemberg|BOLD:AAU2938
Tiphia unicolor|BC ZSM HYM 16988|F|Germany|Baden-Wuerttemberg|BOLD:AAU2938
Tiphia unicolor|BC ZSM HYM 08197|F|Germany|Hesse|BOLD:AAU2938
Tiphia unicolor|BC ZSM HYM 13363|M|Germany|Thuringia|BOLD:AAU2938
Tiphia unicolor|BC ZSM HYM 13361|F|Germany|Thuringia|BOLD:AAU2938
Tiphia unicolor|BC ZSM HYM 13360|F|Germany|Thuringia|BOLD:AAU2938
Tiphia unicolor|BC ZSM HYM 14391|M|Germany|Brandenburg|BOLD:AAU2938
Tiphia unicolor|BC ZSM HYM 14390|M|Germany|Brandenburg|BOLD:AAU2938
